# Fathers’ experiences of childcare and feeding: A photo-elicitation study in a low resource setting in urban Addis Ababa, Ethiopia

**DOI:** 10.1371/journal.pone.0288487

**Published:** 2023-07-21

**Authors:** Hanna Y. Berhane, Dagmawit Tewahido, Workagegnhu Tarekegn, Jill Trenholm

**Affiliations:** 1 Nutrition and behavioral science department, Addis Continental Institute of Public Health, Addis Ababa, Ethiopia; 2 Department of Women’s and Children Health, International Maternal and Child Health, Uppsala University, Uppsala, Sweden; University of Pavia: Universita degli Studi di Pavia, ITALY

## Abstract

Children’s health and wellbeing studies focus mainly on mothers’ roles while very little is known about the experiences/challenges that fathers face in fulfilling their responsibilities. Therefore, this study aims to explore the fathers’ lived experiences of childcare and feeding in an urban low-income setting. This qualitative study was conducted in Addis Ababa, Ethiopia. Photo-elicitation was used to facilitate the in-depth interviews with fathers of children below the age of five years. All interviews were audio-recorded, transcribed and translated verbatim, followed by a thematic analysis approach. The overarching theme of this study was “Fatherhood as an enduring identity”, which comprised of three sub-themes: 1) Blessings of fatherhood, 2) Adjusting to fathering roles, and 3) Struggles/demands of fatherhood in a low-resource setting. Fathers expressed that having children or becoming parents was a blessing. They expressed their love, devotion, and attachment to their children. Some used the term "my second chance in life" underscoring the importance. Although fathers strived relentlessly to spend time and care for their children, they faced challenges such as internal struggles adjusting to and fatherhood whilst maintaining a sense of their former self. As well, providing for their families amidst added pressures imposed by the external environment, such as poor housing conditions, a lack of employment opportunities, the then COVID-19 pandemic, further increased their stressors. Most fathers were engaged in child care and feeding, suggesting that like mothers, fathers should be viewed as potential agents for implementing nutrition interventions in this setting. However, if interventions are to be successful, they need to incorporate components that boost fathers’ livelihoods and general wellbeing.

## Introduction

Nearly half of all children below the age of 5 years in low- and middle-income (LMICs) are at risk of reaching their full developmental potential due to nutritional limitations [1]. Children who fail to reach their developmental potential, are more likely to have poor health, nutritional issues, and learning disabilities, resulting in lower earnings and lower social status as adults [1, 2]. One of the underlying factors that contributes to child malnutrition is inadequate caregiving. It impacts feeding practices, the physical and social environment in which the child interacts, and the emotional support that is crucial in creating a healthy and safe environment for children to thrive [2–4].

Programs or initiatives that aim to improve the nutritional status of children almost exclusively focus on mothers as the key figure [5, 6]. Despite her often low status and lack of economic independence, it has been particularly her decision-making power and autonomy that has been the primary focus when it comes to child feeding and nutrition. Studies indicate that higher levels of women’s status such as higher decision-making, autonomy, ownership, mobility, leadership, and education are associated with positive child nutrition and health outcomes [7–9]. A recent study, a pooled analysis from 9 Sub-Saharan African (SSA) countries, also found that children who had mothers in the highest empowerment quintile had better cognitive development, better diet diversity, and lower risk of stunting [10]. A woman’s caregiving responsibilities have traditionally been more hands-on, while the fathers were mainly responsible for providing financial resources for their children [11, 12]. Although this is changing; in low-income settings, the role of fathers concerning child health and nutritional outcomes is often overlooked, with only a few studies touching upon it superficially, focusing solely on socioeconomic status [13, 14].

Urban challenges such as men’s unemployment and high living costs are limiting women’s former ability to devote all their time to childcare, forcing men to assume more responsibility for the care of the children [15]. In Sub-Saharan African (SSA) countries, urban population growth is primarily attributed to migration from rural to urban areas. This, in turn, contributes to high unemployment, lack of basic infrastructure, such as sanitation and housing, and food insecurity [16, 17]. Furthermore, with rapid urbanization, childcare is becoming less of a communal responsibility, resulting in families having to accommodate this shift by requiring fathers to take on more care responsibilities [15, 18]. In order to create contextually relevant policy recommendations concerning how to increase/support fathers’ involvement and further engage them in efforts that aim to improve children’s nutritional status and promote healthy diets, it is essential to understand their actual experiences. Thus, this study aimed to explore fathers’ experience/challenges in childcare and feeding in the urban context of a low-resource setting in Addis Ababa.

### Theoretical concepts

The theoretical frame that inspired this research, as well as served as the proposed analytical lens, is the concept of caring as described by Fisher and Tronto (1990). In their seminal paper, Towards a Feminist Theory of Caring, they make a distinction between caring about, taking care of, caregiving, and care-receiving thereby widening the concept of care to more than just hands-on caregiving associated with mothers. The theory provides structure in looking at modes of care. As well, we employed the ideas of Social Practice Theory [19] and the construct of masculinity [20] which added a useful dimension; seeing food/eating and caring as examples of social practices that are performed whilst interrelating in the domestic sphere amongst gendered subjectivities. Social practice theory accounts for the performative and dynamic character of daily life, encompassing what is done as well as what is said in much the same way that masculinity is constructed through performativity. Individuals perform care practices to which they attach specific meaning or value using the specific resources they have at their disposal. These practices are dynamic and therefore susceptible to change. The purpose of this study was to understand fathers’ experiences around childcare and feeding in a rapidly changing context. This evolved from earlier research that demonstrated the struggles of urban mothers trying to balance their multiple roles whilst providing adequate care and nutrition for their children [21].

## Materials and methods

A qualitative exploratory design was followed, using in-depth interview methodology enhanced by photo elicitation. The study was conducted in Addis Ababa, the capital of Ethiopia. The city is one of the fastest-growing cities in Africa and the home to almost a third of the urban population in Ethiopia [22].

Lideta sub-city, which is one of the eleven sub-cities/districts, was selected as a study setting. In selecting the area, three criteria were taken into account: it is an inner district, representing one of the fastest-expanding areas, and has the highest population density. Lideta is further divided into 9 sub-districts called Woredas, each of which has its administrative structure, leadership, and health centers.

To understand the father’s experience through their utterances, we conducted in-depth interviews augmented by photos, with 10 fathers who had children under the age of five years. The fathers spoke to the photos they took, adding a visual dimension that enhanced the richness and flow of the interview. Fathers with children aged 0–59 months who had lived in the Lideta sub-city catchment area for at least 6 months were invited to participate. As part of the recruitment process, efforts were made to diversify fathers by type of employment, education level, age, and community involvement. This was accomplished with assistance from urban health extension workers, who had a list of all households and their profiles in their respective catchment areas. Participants in this study ranged in age from 28 to 45 years of age; the mean age was 34. Except for one single father, all the others were married and had more than one child. In response to how many individuals live together as part of their family, the size ranged from 3 to 6. All of the fathers were involved in income-generating activities, ranging from government employment to street vendors to daily laborers and unspecified private work.

During the first meeting, the study participants provided informed consent after receiving detailed information on the study objectives and their role in the study. Furthermore, the fathers were provided with a digital camera, given a 45–60 minutes orientation on its use, instructed as to what types of images they would capture for the discussion/interview, and other information on ethical concerns. Following that, participants were given 5–7 days to take the pictures before scheduling the interviews. The interviews departed from the participants’ visual images and were guided by selected broad open-ended questions concerning (i) the significance of images; (ii) daily activities; (iii) family roles.

Experienced members of the research team skilled in conducting in-depth interviews facilitated the interviews. The focus of the interview was the participants’ interpretation of their chosen photos and the goal was to probe deeper where necessary into the areas that address the aim of the study. This technique of starting a conversation using a scene from the participant’s daily life as a stimulus allows for a more nuanced and rich depiction of the participant’s context and has been previously used in research with youth and marginalized populations where little is known [23]. This technique has also been adapted in public health research where the context is vital to understanding public health phenomena [24]. The participant’s voice is essential in revealing the meanings attributed to the images, thereby restoring the power of self-representation to the participant in the implicit power imbalance between researcher and researched [23].

Considering the COVID-19 pandemic was ongoing, extra precautions were taken to ensure the safety of both the researchers and interviewees. Interviews were conducted in large halls at our office where there was adequate ventilation. In a few instances where the fathers were not available to come to the office, interviews were conducted privately outdoors at the health centre compounds. In both instances, the interviewee and the researchers wore facemasks.

All interviews were carried out in Amharic and transcribed verbatim from the audio recordings. English translations were used to complete the analysis. The Ethiopian team members were proficient in both English and Amharic language, which made verification of transcripts possible. The data were then uploaded onto a password-protected laptop which only authorized members of the research team were allowed access.

The resultant transcripts were read and re-read by the four authors and analyzed through the aforementioned theoretical lenses, using thematic analysis [25]. This involved multiple readings by the diverse members of the research team in search of salient themes that responded to the research question. The team then met to refine and define the themes further, using a mapping technique as described by Braun and Clark (2006), until consensus was achieved.

Trustworthiness was strengthened through close collaborations with the local research partners, who served as cultural brokers. Researcher reflexivity was facilitated through the recording of field notes, adjusting the plan as necessary, and giving rigorous attention to the process of iterative translation so that the transcripts reflected the original voices as much as possible.

### Ethical considerations

The study was approved by the ethical review committee of Addis Continental Institute of Public Health reference number: ACIPH/IRB/003/2020. Additional permission letters were also obtained from the Lideta sub-city and Woreda health offices. Written informed consent was obtained from participants after they were informed of the study objectives, procedures, risks, and benefits. In addition, all transcripts were anonymized and the photos were used exclusively for research purposes, facilitating the interview.

## Results

The findings of this study were summarized under one overarching theme: Fatherhood as an enduring identity. Participants explained in detail their experiences as well as the gradual process of adapting to fatherhood, emphasizing that fatherhood has its ups and downs, but regardless they felt highly responsible and indicated that their roles required necessary sacrifices. These experiences are presented in three sub-themes: 1) Blessings of fatherhood, 2) Adjusting to fathering roles, and 3) Struggles/demands of fatherhood in a low-resource setting.

### Blessings of fatherhood

Fathers described fatherhood as a blessing, a joy with purpose. For some fathers, this feeling of being so essential to another living dependent was demonstrated by their intense attachment to their children; they worried about what would happen if they were not with them or if they had to go away for a short period.

*Sometimes*, *I think*, *what if I have to go away for work*? *I also think I am human*, *something might happen to me*. *These thoughts repeatedly bother me*. *-F2*

For others, their children were described in terms of “my wealth” or “my second chance in life”. They expressed their gratitude for being blessed with having children and how that experience had changed their lives. Even though they were content with their decision to become fathers, they also acknowledged the mixed feelings of anxiety and excitement that come with becoming parents.

*Being a father is great. It is something you dedicate your entire life to. Life before and after you have children is not the same; it is a source of anxiety and excitement… my reason for living currently. -F5*

*For me*, *I believe that being a father is a blessing*. *It is a huge blessing*. *I think that if I were not married and if I did not have my children*, *my life would have been ruined*. *-F3*

The fathers expressed the love they have for their children, saying “Fatherly love is different”. They showed their love and commitment to their children in different ways beyond just providing the basics. The fathers were aware of the need of spending the right amount of time with their children as well as encouraging their children’s growth by interacting with and stimulating them through play. Furthermore, these men recognized the need of assisting their wives by performing more hands-on caregiving activities such as bathing, dressing, toilet training, and assisting their children with schoolwork and other educational activities.

*When we were thinking about having a baby in the first place*, *it was to share whatever type of responsibility comes with it*. *I would dress him (the child)*, *change his clothes*, *and give him potty when he needs*. *I do not see how fatherly love is shown without doing any of those things*.*—*F8

Other fathers, showed their devotion by stepping up and becoming a role model for their children, reasoning that one must live an exemplary life. Fathers were said to prioritize their children’s needs and wants over their own. In some instances, the dads sacrificed their comfort and need to guarantee that their children’s desires were met. Moreover, they expressed that when you become a father, you are taking on the duty of caring for another life, and it is your responsibility to do whatever it takes to provide a better life for your children, a better life than your own.

*Once he has children*, *he must see to it that they are raised right*. *He must be able to give his children a better chance than the one he had*. *-F1*

*Fatherhood*, *as I said before*, *requires a lot*. *A father does not want comfort for himself*, *he wants it for his children-F3*

### Adjusting to fathering roles

According to these fathers, becoming a father requires an adjustment to their lifestyle. The transition from an independent, carefree individual to a family man with responsibilities beyond themselves was often accompanied with feelings of stress and anxiety. Their general perception was that each man/father should carry this responsibility alone. This idea is embodied in cultural and self-imposed expectations that males are tough, emotionally strong, and ought to conceal their vulnerabilities including feelings. This father refers to his obligations as a man:

*A man must be strong*. *He must be able to bear responsibilities…despite the responsibility he shoulders*, *he must also be emotionally strong-F1*

For some fathers, their families and children were motivators to push themselves to work harder because they wanted to provide the best for them. They did, however, indicate that they struggled with balancing family-work time as well as getting personal time. Their relentless efforts on their own to provide for their families could leave them feeling alone at times, as they sacrificed their personal time and social life, making their family/children their sole focus.

*Father’s behavior is not just what you see in pictures since it requires many sacrifice*s. *-F1*

*Being a father is difficult*. *You make a lot of sacrifices*. *Especially in our society*, *fatherhood is difficult*. *In our society*. *You have to make time for your children*. *I do not have a social life outside of my family*… *they (the children) are my friends*. *-F5*

The adjustment at times was said to necessitate sacrificing personal demands, where the dads were required to prioritize the family’s needs and particularly their children’s, over their own; managing in a context of those who have and have not sufficient resources.

*I may have only one shoe and I will be just fine*. *But kids*, *naturally*, *even want new shoes if they see the neighbor’s kid wearing new shoes*. *So*, *one needs to prioritize his kids when it comes to things like this*. *It requires doing what your kids want in order to make them happy*. *Your kids’ happiness will in turn make you happy*. *-F2*

### Struggles/demands of fatherhood specific to the low-resource setting, exacerbated by the COVID pandemic

This theme captures how the fathers in this resource-limited setting struggled to alleviate the financial and material hardships of their families. In particular, the fathers mentioned their poor housing conditions, crowding, and the inequalities in the city, as contributing factors to their stress.

Fathers aired out their frustration in different ways as this participant indicates:

*Some fathers deflect their problems onto their children*… *when people ask them why they do that*, *they answer that their kids are demanding things they can’t afford*. *Sometimes you can just see that they are deflecting their struggles onto their children*. *We live in a neighborhood filled with Kebele houses*, *and the houses are closely packed; while across the street*, *there are families that live lavishly*.*–F2*

An additional challenge is that fathers expressed how they often lacked the opportunity to find stable employment opportunities that can help to provide for their families. As a result, they experienced a constant struggle to ensure their families get access to necessities such as housing, cloth, food, and medical care. This father expressed the inequalities in the city by comparing his worry about how to survive versus other families spending carefree.

*I had a difficult time contemplating how we would be able to survive*. *I waited in line to buy things worth 300 Birr while someone in front of me was buying things worth over 10*,*000 Birr*. *There is a wide gap between the lifestyles of different people*. *For someone with a family*… *life is very difficult at that point*. *The economic challenges were severe; one used to move around freely to work but could not do so at that time*. *If one wanted to buy clothes*, *he could not*.*–F3*

These challenges were said to be further exacerbated by the COVID-19 pandemic. For some fathers, the pandemic resulted in the loss of their jobs while for others they were able to keep their jobs, but still found it difficult to work additional jobs to supplement their income. The loss of income was said to have forced families to tap into their already limited savings and survive on less, cutting down on expenses, particularly limiting consumption.

This father tried to remain optimistic by saying although the pandemic came with a lot of loss, especially in terms of working opportunities some alternative forms were also created.

*Because of COVID-19*, *I have gotten a 25% advantage and a 75% disadvantage*. *Because previously I had opportunities to work in multiple places to supplement my family’s income but now*, *I only have this job (a new job disinfecting client’s office*, *and home) so this is not a promotion*. *-F10*

This father’s experience exemplifies how COVID-19 has impacted individuals’ livelihoods directly through loss of jobs; forcing families to make difficult choices between earning and possibly spreading the virus to the family.

*It has affected me in many ways*. *I was working in construction and that stopped because of COVID*. *That is how I was affected but I thank God that I am healthy*. *We also heard the Ministry of Health recommending physical distancing*. *That was difficult to implement given our housing situation therefore my wife had to even decide to no longer wash clothes for people*. *We did not have anything to eat because of that*. *It has disrupted a lot of things*. *-F8*

In addition to the economic impact, Covid-19 had also affected the father’s social life. Due to fear of the unknown especially at the beginning of the pandemic, families were restricting social contact which left them feeling isolated and fearful.

*We were hurt psychologically*, *very much hurt at first since we didn’t know much about the disease*, *and we were very worried/scared…we did not connect or meet with people we isolated strictly*. *Second*, *we were highly affected economically*. *-F9*

The pandemic was also cited to have positive effects as the restrictions and lockdown enabled families to spend more time together. Fathers mentioned that it increased communication with their partners as well as their children and allowed them to bond with their children.

*Economically*, *it has impacted us a great deal*. *There are no jobs*, *you spend most of your days at home*. *We eat whatever we have at the house*. *Then progressively you use up your savings*. *However*, *the time since the pandemic has helped me spend time with my daughter*, *and that has strengthened our bond and connection because we are both at home 24 hours a day*. *-F2*

## Discussion

The results of this study highlight the difficulties involved in reconciling childcare with personal experiences, work/family expectations, and contextual obstacles. We found that fatherhood was described as an enduring identity that comes with its ups and downs; while the transition to parenthood was largely joyful, it also involved changes that required fathers to adjust their identities, relationship, time commitments, and their expectations. Further, contextual hurdles related to rapid urbanization and the current pandemic and all that entailed seemed to amplify their constrained situations.

This study found that fathers’ role in childcare is more than just a set of skills or personal attributes, but rather an integrated process with multiple dimensions. This is in line with Fisher and Tronto’s care theory [26]. According to this theory, care is a process, with four dimensions: caring about, taking care of, caregiving, and care-receiving [26]. The first two, ‘caring about’ and ‘taking care of’ involve more intangible aspects of care such as paying attention, love, devotion, and attachment which require more mental and emotional connection work. Throughout the first theme, Blessings of fatherhood, we observed fathers having a strong attachment to their children which they were quite explicit about, contrary to earlier findings. According to a multicountry study from four African countries, fathers in all sites had little or no direct involvement in newborn care, and their primary role was as a financier of the family [27]. Receiving care from both parents is critical for child growth and is strongly associated with positive child developmental outcomes [28, 29]; however, it is often overlooked or assigned as the responsibility of mothers [27, 30].

The fathers in our study were quite involved in childcare and worked relentlessly to fulfill their children’s emotional as well as physical needs; this might be a result of the gradual change in gendered roles that other Ethiopian studies have also reported. Studies conducted in southern and northern parts of Ethiopia both reported that fathers’ perceptions of childcare and feeding practices are changing gradually, attitudes toward childcare have shifted to a shared one where fathers became more involved in the care of sick children while also taking on the burden of financial earning [31, 32]. A study from Cameroon [15], also revealed a similar shift in childcare responsibilities where men have taken on more childcare responsibilities as a result of urban changes requiring women to engage in income-earning activities. Furthermore, it also highlighted the fact that fathers are increasingly conscious of the fact that investing in the proper upbringing of their children leads to greater returns for themselves and their country [15].

Caregiving is the third dimension captured in our findings; it is comprised of concrete actions that demand time and resources. Regarding this dimension, our findings show the fathers took part in cooking, feeding, playing and interacting with their children along with several other activities to fulfill the needs of their children. Although the study participants were keen on taking on more responsibility, the realities were that they were under pressure to seek a balance between other competing demands such as paid work. In alignment with this, a recent systematic review that explored the experiences of first-time fathers [33] revealed that despite their excitement to take on additional responsibility, they were constantly worried about whether they were doing things right, balancing providing for their families financially and fearing they would miss out on time with their children and family. Contemporary fathers are increasingly involved in childcare and domestic responsibilities beyond financial support; despite yielding positive child outcomes, this evolving new role and responsibility is a stressful one [34–36].

In urban low-income settings, resources are scarce and financial constraints are a constant challenge. For some men this entailed working longer hours or away from home, to support their families, sacrificing the time they spend with their children. For others, it brought a shift in their roles whereby fathers took on the primary caretaking role while mothers worked outside the home to provide for their families. In similar urban settings in Africa, studies demonstrated that urban complexities and economic pressures have contributed to a shift in gender roles in the household with more fathers and husbands becoming active in childcare [15]. Although there is limited evidence in this context, studies from higher-income countries have attested to this shift, however, they note that without adequate system-level support, fathers’ time spent in childcare is highly variable depending on the employment characteristics of both parents, including who earns more and whether they have flexible work hours [37, 38].

Further intensifying their experience, fathers in our study expressed that males are expected to be strong. In this culture and across several societies masculinity or what it means to be a man is represented by certain traits such as strength, independence, and ability to provide for one’s family; while the contrary, being compassionate or showing emotion is a sign of weakness. In line with this, an earlier review of fathering texts highlights that there is a complex negotiation that men face in relation to balancing fathering identities and masculinity; fathers are depicted as financial providers and caregiving is often portrayed as feminine [39]. Connell’s hegemonic masculinity discourse has dominated the research for decades, indicating a very specific male construct [20], however, recent findings are showing that with evolving social, and economic environments/contexts other constructs around fatherhood and what it means to be a masculine parent figure, both self- and societal idealized, is very slowly is shifting [15, 40].

This shift demonstrates how different contexts and resource availability affects the roles and responsibilities of fathers. Despite their willingness to adapt, they are fearful that their new roles may be considered weak and vulnerable. Supporting fathers in their changing roles is in part, the responsibility of an evolving society and its institutions. Furthermore, while care receiving, the fourth dimension, was not explicitly discussed, the father’s need for care was frequently implied. Therefore, to make the transition smoother; potential interventions that support fathers could be in the form of mentorship programs or fathers’ groups where they could exchange ideas and share their struggles [41, 42]. Having a support mechanism in place, or developing father-friendly resources could be useful strategies that would support the father role transition. In accordance with this, studies have featured some potential alternatives to engage fathers and support them in their transition to a more participatory role in childcare, including using internet-based strategies to deliver tailored messages, supporting early engagement for fathers to take a more active role in their child’s life starting with pregnancy [43, 44].

Rapid changes in lifestyle and economics in these expanding urban areas have also resulted in changes in family structures and social dynamics whereby all adults in the household are required to work for longer hours leaving no one available to support/ assist in childcare within the collective. Moreover, studies from urban low-income settings have revealed that with deteriorating traditional support by grandparents or extended family members; parents are struggling to find affordable, accessible, trustworthy, and quality childcare alternatives, especially among low-income earners [45, 46]. It is therefore inevitable that any effort to improve child nutrition and growth by increasing father involvement will prove unfruitful without taking the entire context into account.

Policies related to health, poverty alleviation, and gender equality have to adequately consider ways to promote men’s involvement and be inclusive. Gender transformative programs which are programs that create opportunities for individuals to challenge gender norms and that engage men and women jointly as agents of change are essential. Drawing experiences from programs focusing to improve sexual and reproductive health and rights, this approach features a multipronged component of education, motivation, and opportunity which yielded positive outcomes in relation to behavioral changes [47]. This approach, if started early in a child’s life could yield a multi-fold return on investment by providing boys with the skills and knowledge required to take on new roles in households and allowing girls to recognize their potential.

### Reflexivity or strengths and limitations

Using photo-elicitation to prompt discussion with fathers was a key strength of the current study. It allowed fathers to control the narrative of the discussion. Having the images of their children while sharing their reflections/perceptions during the interview may have helped/allowed fathers to stay in that mental space to have richer conversations about the community, their experiences, and feelings. A testament to this is the depth of information we received from the fathers. Fathers discussed matters they felt were important to them and often revealed their vulnerabilities, which could be because images, as opposed to traditional oral interviews, are powerful prompts, contributing to unveiling the interviewees’ social worlds and critical assessments. Through the inclusion of pictures, the fathers were able to show their context permitting the researchers’ ability to grasp their realities, thus enabling richer interpretations of the actors involved and broader analysis. It is somewhat of a contradiction to deny vulnerability and then to express it. However, this could be that, in the process of orientation, taking pictures, and selecting the images, the researcher and interviewer spent more time than in a traditional interview; this supported the connection the fathers felt to unburden themselves and enhanced rapport building.

Taking multiple photographs and the option to take pictures on different days provided opportunities to see various scenarios which may more accurately reflect the father’s wider engagement with this child. The number of images could at times pose a challenge in sorting and selecting images that best represented the fathers. Despite our best effort to minimize bias, we cannot fully rule out the potential for social desirability bias, if the fathers believe that the right style of fathering is one of engagement, then this engagement could be overstated. If fathers expected they might receive some assistance from the study they might exaggerate their poor living situation. That said the photos served as a way to verify the living situations the fathers depicted.

This study illuminates’ fathers’ roles in childcare and feeding in low-income urban settings; it demonstrated how fathers are taking on more responsibility beyond financial support and have a desire to be more involved in hands-on caregiving. The findings also underscore the personal joys and challenges of the changing roles of fathers and the adaptability imposed by rapid urbanization in this setting. Fathers are a vital resource and should be viewed as potential agents to include in implementing child growth and nutrition interventions. However, if such interventions are to be successful, they need to incorporate an understanding of the components required to boost fathers’ livelihoods and general well-being.

## Supporting information

S1 File Supplement table: Interview guide. (DOCX)

S1 Data (PDF)
