# Supplementary material for: Fathers’ experiences of childcare and feeding: A photo-elicitation study in a low resource setting in urban Addis Ababa, Ethiopia
Source: PLoS One. 2023 Jul 21;18(7):e0288487. doi: 10.1371/journal.pone.0288487 (PMC10361465; doi:10.1371/journal.pone.0288487)
Supplement: S1 File — (DOCX) [file pone.0288487.s001.docx]

***Supplement table: Interview guide***

| Question 1 | Tell me the story of this photograph.  Probes:   - *What stands out for you in this photo?* - *What else is happening in the picture?* - *How does this relate to your life and life within your community?* |
| --- | --- |
| Question 2 | What does it mean to be a man in your community?  Probes:   - *What about fatherhood?* |
| ***Question 3*** | How do you see your role in the care and feeding of your children?  ***Probes:***   - *Why does this issue exist?* - *Have you encountered any challenges in fulfilling your role as a father? Do opportunities exist?* - *What can we (or others) do about it?* - *What would you hope for this scene to look like in the future?* |
| ***Question 4*** | Is there anything you would like to add about what we have discussed? |
| ***Question 5*** | Looking at all these photographs together, what story do they tell about your role as a father in the care and feeding of your children? |
|  | In the absence of a photograph, for whatever reason, the interviewer can  simply start with a broad open-ended question like “Tell me about how  you see your role as a father” or “Can you say something about your role  in the care and feeding of your children.” One can continue by using the follow-up questions listed above. Additional probes and follow up  questions could be created in line with the aim of study if new ideas are emerging. |
|  | ***Thank you!*** |
