# Supplementary material for: Fathers’ experiences of childcare and feeding: A photo-elicitation study in a low resource setting in urban Addis Ababa, Ethiopia
Source: PLoS One. 2023 Jul 21;18(7):e0288487. doi: 10.1371/journal.pone.0288487 (PMC10361465; doi:10.1371/journal.pone.0288487)
Supplement: S1 Data — (PDF) [file pone.0288487.s002.pdf]

Father 1

Interviewer: Thank you for coming and agreeing to take part in our study. My name is ________ and I am a member of the research team. I will now show you the pictures and we will discuss.

I: You have selected this picture first so please tell me what you see on this picture. What aspect of this picture stands out to you?

Respondent: As you can see, they are sharing a meal and feeding each other. The oldest one is feeding his younger sister and teaching her how they can dine together. I always encourage him to teach her *(younger sister)* so in response to that, the older brother took the meal from me and started feeding his younger sister.

I: How old is the eldest child?

R: He is 4 years old.

I: How about the youngest one?

R: She is 1 year and 8 months old.

I: What else do you see in this picture? With respect to what they are eating?

R: They are eating vegetables. Kale specifically. I always try to provide a balanced diet, depending on what I can afford.

I: Could you please elaborate on what you mean by a balanced diet?

R: What I mean by that is, if they have a certain meal for lunch, they will have a different type of meal for dinner. Depending on how much I earn and what I can afford, I always try to provide balanced meals for them.

I: How would you relate what you see in this picture to your lifestyle and that of the community or society?

R: How it relates to my lifestyle is, as you can see, I live for my kids. Other members of the society, especially those with families to take care of, probably strive to provide for their families. I do not believe it will be harmful if the society adopts such behaviors.

I: Shall we proceed to the other pictures?

R: Yes.

I: What do you see in this picture?

R: In this picture, I was in bed. My kids were already awake, and they were trying to wake me up by playing with me. They were waking me up.

I: How about this one?

R: I was at work during the day and I always come home with milk for the children, at the end of the day. As I walk in, *(the youngest daughter)*, she was taking the milk from me. That is what the picture shows. She then takes the milk to her mother so that she can boil it, cool it, and then give it to both children.

I: What do you think is the importance of milk for children's diet?

R: Milk is important for their health. Secondly, it will make their teeth stronger. Milk is extremely important. First of all, breastmilk, at least for six months, without any additional food, is enough for children. Because of the extreme importance of breastmilk for children, my fear is if my daughter might not be getting adequate breastmilk, the milk I buy for her might be able to supplement for her needs. So, at the end of each day, I bring home milk for both children.

I: What does this picture show?

R: This picture shows how important exercise is for our health. I always tell him how important exercise is, so he is willingly do it and I was encouraging him. He *(the eldest son)* asked if he could exercise now and I agreed. Because he is a child, I did not want him to get hurt, in case he falls, so I placed a mattress for him to practice on. Progressively, he will practice outside. I show him, at a slow pace, and create a safe environment for him to learn.

I: What does this picture show?

R: This picture shows the kids playing. They were playing holding two dolls. When I am around, I never restrict them from playing together. I help them role-play and I am the one that buys them their toys. They might see other toys that the neighbor's have and ask me to buy them similar ones. I buy these dolls for them and allow them to play with it.

I: Thank you. If you have anything else to add or elaborate on, on the five pictures we just saw, please do.

R: What I want to add is, a father's behavior is not just what you see on pictures since it requires many sacrifices. Parents make several sacrifices for their children. They are obligated to make those sacrifices. A father, I mean. Therefore, I want to add a few things. Firstly, regarding family planning, child spacing is important, for the sake of the children's health and safety. Secondly, we *(parents)* should strive, within the limits of our financial ability, to provide balanced diets for our children.

I: Okay, thank you. Could you state a few examples of what you mean by balanced diet? Which food groups can be included in the category of balanced diet?

R: Eggs, vegetable, vegetables, eggs, fruits, these are a few.

I: In the society you live in, how do you describe masculinity? How do you describe the behaviors of a man?

R: Man, meaning… *(pause)* A man must be strong. He must be able to bear responsibilities. Despite the responsibilities he shoulders, a man should stay strong emotionally as well. He must carry out his duties adequately and be hardworking, in whatever he does to earn his income, and provide for his family.

I: You have described masculinity. Now can you describe fatherhood? What should he *(a father)* be for his children and for his family?

R: Fatherhood implies similar behavior as being a man. A father must work hard and strive to raise his children. Once he has children, he must see to it that they are raised right. He must be able to give his children a better chance than the one he had. A lot has improved nowadays so a father must aim to

educate, feed and raise his children in a better situation. If he educates them and raises them right, then they will have a better chance of achieving great things, given that God gives us a long and healthy life.

I: Please feel free to elaborate or add on what you have already said on fatherhood.

R: I think I have said enough. Fatherhood can not be just described in words since there are various aspects of it that are difficult to explain.

I: Can you describe your role in the feeding and caregiving of children?

R: My role includes taking care of the children's personal hygiene. All the materials they need to take care of their bodily hygiene is placed separately including the soap and tub they use *(to wash their body)*. Once they take a bath, they will change into properly cleaned clothes. In addition, I make an effort, within the limit of my financial capability, to provide them with a balanced and healthy diet. My eldest child has now started school and I follow up on his progress daily. I ask what he has done that day and what he has learned. He tells me what they did and learned in school. I ask if he has any homework and if so, I help him read and do the homework. I showing him some of the alphabet letters and help him study. Wasn't there a picture that showed him studying?

I: You told me earlier that you help him study so tell me what catches your attention in this picture?

R: I sit down and he *(the eldest child)* stands up. I have put the Alphabetical Chart on the wall. The reason why I have made him stand is so he can pay close attention to every letter on the chart as he points to it and reads. The first day, I have stood up with him and helped him study each letter. But on the second day, he did it by himself while I followed and helped him memorize each letter. I tell him to point at a letter, ask him if he has seen the letter he is pointing at attentively, he answers yes, I have seen it attentively, I tell him to read it out loud, he does, and we go on like that so that he knows the location of each letter.

I: You have informed me of your role in the children's feeding. Can you tell me what challenges you have faced and if there are good aspects?

R: What I have noticed is that, the concept of child feeding is relatively new to me. I had little understanding until recently. The health center has started providing counselling service regarding this issue. Since then, I have come to understand a few things. I made no effort previously. The reason behind is, when we talk about balanced children's diet, there are financial constraints. It depends on each person's financial capability. For instance, people who have better financial status can afford to provide a balanced diet for themselves and for their children's wellbeing. Coming back to myself, even though I have low socio-economic status, within my limits, I feel the need to raise my children better than how I was raised. I had an entirely different childhood and I want to break the cycle and improve on how I raise my children. For this reason, I have a firm belief that I should provide balanced diet for my children, within my financial capacity.

I: What do you think can be done in the future?

R: What do you mean by that?

I: In addition to what you are already doing, what do you think can be done in the future?

R: In the future, I have to educate my children. I have to be able to shape them into capable citizens for the future.

I: You have told me previously that fatherhood is not only portrayed in pictures and that it is way beyond that. What do these pictures show regarding your role as a father?

R: At this particular age, children, as long as they are not exposed to any harmful things, should have the freedom to play with each other. I am trying to do everything that I believe is right, according to my understanding. I don't have much to say beyond this.

I: What can these pictures show regarding your responsibilities in your children's feeding and caregiving? *(Brief pause here)* Do you understand the question?

R: No.

I: What can these pictures show regarding your responsibilities, as a father, in your children's feeding and caregiving?

R: These pictures show that I do everything within my capacity. Furthermore, these pictures show what fathers teach their children, what they do for them, and what they must do for them. This is what the pictures show.

I: Thank you. As you know, since the COVID-19 pandemic, various changes in our country are evident. What changes have you noticed within your family as a result of the COVID-19 pandemic?

R: Since the corona virus pandemic, there have been changes in my family. We didn't choose the pictures that show this but there are various changes. When I come home from work, I wash in the back before I enter the house and greet my children. I always wear facemask wherever I go. My son wears a facemask when he goes to school and so does my wife when she leaves the house. We have alcohol hand-rub in our household. I also use alcohol hand-rub once I wash and dry off my hands.

I: What changes have you noticed beyond your household? You have told me of the precautionary steps that you take. But what changes have you noticed on your social life?

R: It has brought about various challenges on my social life.

I: For example?

R: For example, previously, I used to work as a street vendor. However, I was not able to continue in that line of work since the pandemic since there is a fear of contracting the virus by receiving contaminated bills. This was a challenge I faced early on in the pandemic. However, now there is a general reduction in being careful and taking precautionary measures within the society. Since then, I decided to be as careful as I can and proceed with my work in order to provide adequately for my family. Rather than dying of starvation, I should take the risk and go on with my work. I work to the best of my abilities to feed my family. Regarding the precautionary steps, a lot has changed as compared to how it was at the initial phase of the pandemic. There are people who do not wear facemasks at all, holding it in their hands, and some who do not wear them properly, only covering their chins. Some even get into taxis[1] without wearing their

[1] Taxi: A taxi, in this case is to mean a 12-passenger minibus that is commonly referred to as just 'taxi' in Ethiopia. They commonly give transportation services In Addis Ababa.

masks and that makes me fearful and suspicious. I can't get off the taxi because lack of transportation is a huge challenge so I don't have many options. So, I still use the taxi, because I don't have a choice. Aside from that, I don't have any contact with people who do not wear their facemasks.

I: How about economically? What changes are there within your household?

R: Economically, there are various strains that I am facing. There is a big difference between what we used to earn previously as compared to now. There is a big difference. Now, the means of earning money are limited. People are not buying from street vendors as much as they used to. They now go into supermarkets and buy in bulk. People don't buy as they used to previously.

I: Is this something that came about since the pandemic?

R: Yes.

I: So, this has impacted your economical status?

R: Yes, very very much.

I: How much of a difference is there between how you used to provide for your family previously, before corona, and how things are now? With regards to your economy?

R: As we have seen in the pictures earlier, I used to buy my children milk on a daily basis. Previously, I bought milk for my children everyday. However, now I only buy milk every other day or once in three days. Secondly, since the market has declined and my capital has diminished, I have run out of stock of what I used to sell. I sold what was remaining piece by piece, over time and couldn't afford to replace it since I used up the money I earned, to restock our supplies at home, like "Shiro"[2] and "Teff"[3]. I used the money for our needs and subsequently I don't have the capital to restock the supplies, meaning that my business is failing. This further impacts my economical status.

I: What kind of impact does this have on your family?

R: The impact it has on my family is what I told you earlier. Before, the market was stable and I had adequate income. So, things were better. Now, my income has decreased by 50%.

I: I believe that we have exhaustively discussed on these five questions. If you want to add on any of your answers, if you want to speak further on any issue, if you want to discuss on any of the points, or if you want to point out matters that weren't raised in our discussion, please use this moment to do so.

R: Okay. What I want to add on our overall discussion is firstly, regarding corona virus, many schools may have been built for expansion and many cleaning materials may have been prepared but we still need to take additional precautions. Children just want to play, be it on the swings of together, and they may not be as careful as we want them to be, especially younger children in 1st to 4th grades, so they need close supervision. Those in high school are almost adults and can take care of themselves. Secondly, the society's carefulness and prevention measures towards the corona virus is extremely poor. There is no way of telling when, how, and where you might contract the infection. As long as this continues to be a

[2] Shiro:
[3] Teff:

global issue, poses a threat to our health, and bring about disastrous impact on Ethiopia, compared to other developed countries, all of us, all Ethiopians, should be extremely careful.

I: Thank you very much for taking the time to be a part of this interview.

Father 2

Interviewer: We will now begin our discussion.

Thank you for giving us your consent to participate in this interview. We have chosen a few photos and we will have our discussion based on them (*selected photos*).

The photo we see here is the first photo we have selected. Please explain this photo for me, in your own words. What do you see?

Respondent: This photo was taken by my son, it is a photo of my wife, kid and myself. This shows, often times, when we are about to eat, my daughter has difficulties eating. Most of the time, she only eats when I get home from work. I feed her playfully, and as you can see in the photo, in my left hand, I have the photo box and I tell her that I am going to give that to her if she eats properly and I feed her. On the other hand, her mother tries to motivate her by saying “who is going to finish first” because she (*daughter*) always has difficulties eating. It is always a challenge feeding her. As you can see, she is very thin. Most times, in the evening, as soon as I get home from work, I sit her (*daughter*) down on the sofa and try to feed her whatever we have at home. As you can see in the photo, it is a competition between her and her mom to see who is going to finish first. And we are doing this so that she eats her dinner. This is around 5pm since we usually take a walk after eating dinner. This is what I have seen in this photo.

I: Okay. What else? Is there anything that you want to add?

R: This means, most of the time, she wakes up in the morning. I usually leave for work before she is awake. Coincidentally, when I get home from work, I ask my wife if she has eaten and she tells me that she has not eaten her dinner by giving “this and that” excuses. Kids usually like competition, especially when seeing other kids. Even while playing, with toys, they will only play attentively when it is with other kids. They will not value it when they are alone. I, within my capability, am served with what we have on hand and I am feeding her as I eat myself. It is like I am trying to lightheartedly feed her. Sometimes, I follow her as she moves around and I feed her.

I: Why do you think she is willing to eat when you feed her? Is it because you do it playfully or because she misses you while you are away at work?

R: Yes, primarily she misses me. For instance, in the morning, if she wakes up before I leave for work, she goes with me until we reach the main road before she returns home. She comes out with me; she sometimes asks me to buy her some things. When I have money, I buy them for her, when I don’t have money, I just trick her and she returns home. I don’t have one fixed job and I mostly do various types of jobs in different organizations taking logistic roles. I used to be a civil servant. Things are somehow different because of the seasonal political conflict. I am not severely bothered by this since things that come with the season also disappear with the season. By doing whatever is available, thank goodness, I always manage to earn enough to take home. Whenever I leave early in the morning and come home at night, she (*daughter*) waits for me eagerly. Sometimes she even calls me during the day, using my wife’s phone, we talk and she tells me to bring lots of things, since they always want something. So, depending on my capability, I bring home whatever I can afford and we eat that together. She misses me, since she spends the entire day with her mother. If you ask her “who do you want to carry you” or “who do you want to feed you”, she will come running to me. I think this is because she spends every day with her mother. If she spent every day with me and her mother wasn’t at home, then I think things would be the

other way around. That is the angle I saw it from. It is because she misses me, she doesn't get a lot of my time, so this happens during the evenings.

I: Good. Why did you choose this picture to bring to us? How did you come to choose it?

R: The reason I chose this photo is because the study focuses on the involvement of fathers in their children's nutrition, eating habit, and food provision. Balanced nutrition has its own rules and guidelines but it still needs to take place with whatever is available, depending on people's affording capacity. Its not always about having meat products and fatty foods. People, with whatever they have at hand, be it boiling a potato and eating it with "mitmita"[1] and call it a day. People should consider it as a blessing since there are others who can't afford to do the same. Life in Ethiopia has taught me a lot. I have traveled to various places within in the country, except to Hawassa and Jimma, and because of that, I have seen the way people in different areas live. As a civil servant, I had the opportunity to have a better standard of living, better house and riches, but I was only exposed to people living below my own standard of living. I have seen people who can't even afford to eat once in 24 hours and who developed different health issues because of that. I, thank God, am happy with what I currently have. I put this together with the objectives of the study, in association with nutrition & eating practices and fathers' involvement & how much time & attention they give to their children. I give what little time I have. Often times, she (*daughter*) won't be awake when I leave or come back home for work. She sometimes believes that I have gone out on a field trip, when she (*daughter*) calls me during the day. So, in accordance with the study, I chose this picture.

I: How can you relate this picture with the society or other people in general? You have already touched up on a few points. To help us understand the points better, please tell me how you can associate this with your personal life or the society's way of living?

R: The way I relate this to the society, the one that I live in, is like this. There are houses on the right and left as well as in the front and back. The ones on the front and back have a better standard of living. The ones on the right and left have a similar standard of living as me. One of them (*the house on the right/left*) in particular, are doing better nowadays, thank God, but were having a rough time previously. So, comparing myself from those surrounding me, I am not at the bottom, nor on the top level of living. I am in the medium level because I am not among those who can't afford to eat or among those who can eat whatever they want, in excess. I, within my capacity, can feed both myself and my family. I don't have anything in excess, nor am I extremely limited. I am in the middle. So, looking around into the society, I am extremely grateful to God because there are others who don't even have what I have.

I: When I look at this picture, I can see how close you are with your children and your wife and I can tell that you have a very happy family. I am saying this because you told me earlier that you see joy in the picture. Do many fathers in your society have similar lives as you? What does their relationship with their children look like? Tell me what you think in this regard.

R: To your surprise, one of my neighbors, God rest his soul, never wanted his wife to wash clothes. He wanted to carry out all the responsibilities and duties of his wife. He was a highly ranked official, an Inspector in the Addis Ababa Police Commission. I have learned a valuable lesson from him. If you are not happy with what you have, then you can never be happy with what you don't already have. He is a big

[1] Mitmita: Mitmita is a powdered seasoning mix used in Ethiopia. It is orange-red in color and contains ground African bird's eye chili peppers, Ethiopian cardamom, cloves, and salt.

role model for me. His children are now happy and successful. One has become a doctor, one successfully lives her life with her own family, and the other one has opened up a store and works there. His *(neighbor)* way of living has taught me a lot of lessons. To look at things from another perspective, I also have various friends whose lives I observe from a distance. When I look at the sacrifices they make for their children, I know that I have a long way to go. Even though I have a long way to go, I don't stress myself too much by comparing myself and others and what we do for our kids. The question I ask myself is what makes my daughter happy? If buying her bananas makes her happy but I couldn't afford 1kg of bananas, then I will buy her ½kg of bananas. Depending on how much I can afford. If one banana makes her happy then I would buy her that 1 banana even if it is 10 birr each. This is what I think. My wife is a homemaker. She is not employed. People repeatedly ask me how I support her. But I believe that being a homemaker and raising children is a job by itself. What people don't understand or comprehend is that you cannot pay someone to raise your kids. Maybe you can understand. I see that there are many challenges that come with it. Washing clothes, cleaning the house, keeping your kids healthy and so on. I am always grateful to God because of how healthy my children are, thanks to my wife. My eldest son used to have repeated attacks of tonsillitis when he was a child and he had surgery for it at Black Lion Hospital. Since then, he is very healthy. When she *(daughter)* gets sick, unless it is severe, we never go to a health facility. She takes Holy Water and "Emnet[2]". So, when I look at this and the society, primarily, I have learned a lot from my neighbor. He used to make "Wot[3]", "Doro Wot[4]". I can't make "Doro Wot" and making "Injera[5]" but these are the only things that I can't do. I can do everything else.

I: Do you help out in your home?

R: I do. I hope you don't think I am exaggerating but, in the society, I grew up in, I grew up in Tigray, I grew up fetching water, taking grains to the mill for grinding, making dough, and so on for my mother. Maybe it's because I grew up going to church which instilled the fear of God in me, to this day. So personally, even when I have absolutely no money, I am always happy.

I: Okay. We have raised very good points. As we have other following photos, we shall discuss on the points that we raised so far for each photo and proceed to the next questions. The following photo you have chosen is this one. What do you see in this one?

R: This picture, in short, shows that if she *(daughter)* doesn't sleep with me, she wont fall asleep. So, here we are lying down and watching TV. As you see, we have a mattress down on the floor, in the salon and that is where me and my son sleep. She sleeps in the bedroom with her mom, we have a bed there. I take her to the bed after she falls asleep. If she sleeps with her mother, she wont fall asleep. She sometimes stays up until midnight and 1am.

I: That means you are the one who puts her to bed?

---

[2] Emnet: Holy ash

[3] Wot: Wot is an Ethiopian and Eritrean stew that may be prepared with chicken, beef, lamb, a variety of vegetables, spice mixtures such as berbere, and niter kibbeh, a seasoned clarified butter.

[4] Doro Wot: Doro wot is made from chicken and sometimes hard-boiled eggs is the most popular traditional food in Ethiopia, often eaten as part of a group who share a communal bowl and basket of injera.

[5] Injera: Injera is a sour fermented flatbread with a slightly spongy texture, traditionally made out of teff flour in Ethiopian and Eritrean cuisine.

R: When she comes to me, I wrap her in a hug and we watch TV, children's shows. I narrate the stories to her and she falls asleep within 10-15 minutes. Amazingly, when I ask her to change her into her pajamas, she refuses because she doesn't want to fall asleep so I usually change it for her after she falls asleep. She keeps saying you are going to make me fall asleep and leave me. Thinking about this worries me sometimes. I think, what if I have to go away for work? I also think, I am human, something might happen to me. These thoughts repeatedly bother me.

I: You worry that she might get hurt?

R: Yes, I worry that she might get hurt because I have seen that happen to my eldest son. My eldest son, when he was born, I was the one who took care of him and my wife, there was no one else around. Until he became 1 years old, I was the one that mostly took care of him. Later, due to some coincidence, I found a job in Jigjiga. Once I left, he repeatedly got sick, he used to cry a lot, crying over the phone too. He even asked his mother if I was dead. We were so confused. The job I had was a good job but it was not worth losing my kids, so I quit and came back home. After I came home, we weren't separated for about a week. We used to go to and from everywhere together. So, what I experienced then is why I worry now. Sometimes, when I go out early in the morning and get home late at night, I worry that she might be hurt.

I: Lets proceed.

R: Okay. This is what worries us currently, the thought of what is going to happen to her *(daughter)* later in life. As long as I am here, thank God, we are not facing that many challenges.

I: You have told us the correlation with your own life. How would you describe this photo from the society's angle?

R: I have told you earlier what families do for their children. In the area we live in, there are no children under the age of 8 or 9, it is only my daughter. So, I can't tell what the interaction within the families is like between parents and their children. I don't always see them around.

I: What I meant to say is, do the children have a deep attachment with their fathers? Are fathers concerned about their kids eating habits, sleeping patters and so on?

R: It is like I have stated earlier. There are no small kids where I live. Previously, there was one small girl, my neighbor's granddaughter, the neighbor I told you about earlier. She had an intellectual disability. She is almost the same age as my daughter, maybe 2-3 months difference. If I call my daughter right now and you listen to how well she has learned to speak, you wouldn't believe that she is only 3 years old. But the girl I told you about can't even say mama and baba yet. Often times, they *(his daughter and the neighbor's granddaughter)* sleep together, in the bedroom. My daughter wakes up after 10 minutes or so but the girl sleeps for hours. My wife leaves the girls together so that they sleep. The girl lives in a different area and besides her, there aren't any other small kids.

I: You have already expressed your worries, about what will happen if you leave for a job, how difficult it will be on your kids and so on. How do you think other fathers in the society think about these issues? In association with this, you have started telling us about what you think might happen if you are not around for your children so what are your thoughts on that?

R: I have a friend, during a financial transaction, he was accused of cheque fraud and was sentenced to 3 months in prison. His child was immediately confused. He *(the friend's child)*, even after speaking to his

dad on the phone, would never eat, unless he visited him in person at the prison. 3 months felt like 3 years to the child. He lost 7-8kgs within those months. So, I asked him *(friend)* why this happened? He told me that he used to play with his son until the child was exhausted enough to fall asleep. That was what the child was missing during those 3 months. During the day, the kid passed the time by playing but at night, he goes to the sofa and asks his mother about the whereabouts of his father. To some extent, for about a month, I helped out by going to their house and spending the night there to play with the kid. But I wasn't able to fill the gap of his father. I think kids also identify people by their scents. He lost 7-8kgs during those 3 months. There were no consequences on his wellbeing, but he changed a lot physically. I take him *(friend's son)* to visit his father at the prison every week. He *(friend's son)* doesn't seem bothered a lot, except that he misses playing with his father. Otherwise, he *(friend's son)* seems fine. From that angle, is why I feel like my daughter would suffer a lot if I went somewhere. There is nothing I value more than my daughter so just as I have done previously, I will leave the job and come back to her so that she doesn't suffer. If I pass away, however, how things would look for her in the future is difficult to imagine. God protect us from that.

I: You have told us about the experience of your friend and yourself and the deep attachment you have with your children. From your perspective, do you think other members of the society have similar experience regarding their relationship with their children? Or do you think it's different?

R: It depends. Occasionally I see some fathers deflect their problems onto their children. They insult them, beat them up, and scream at them. And when people ask them why they do that, they answer that their kids demand of them, things that they can't afford. Sometimes you can just see that they are deflecting their struggles onto their children. Kids immediately behave when you even make minor effort. In our neighborhood, there are Kebele houses, and the houses are closely packed. The houses across from us are people who are well off. They are millionaires who own shops, cars, and so on and they could afford to put up Wi-Fi or 55-inch TVs in each of their kids' individual bedrooms. On the other hand, if my kid demands that I buy a 34-inch TV to replace the 14-inch TV that we have now, I can't do that. When this happens, I protract my anger onto him *(son)* by screaming. Some might even say, its not enough that I feed you, now you want to be picky about TVs! I, however, don't believe this happens because these people hate their children. It is their way of living that makes them like this. Before I got married, in 1997 E.C., I used to live with my sister. We used to live around 4 kilo, around the big buildings when you go to Kidiste Mariam. This guy, he worked in European Union/United Nations, and when his kids got angry, they would just get up and break their TV. As soon as they break it, he would just bring another one. This always amazed me. I would think, if this kid breaks the TV and his dad would just buy another one, maybe tomorrow if he tries to hurt his dad, what is the father going to do? So, the guy should have tried to teach his kid a lesson from the start of the minor misbehavior.

I: To summarize what you have already told us, you have told us about your friend's and your own experiences, and that it differs from person to person when it comes to other members of the society.

R: Correct.

I: It differs based on the situation and the way people are raised.

R: Correct.

I: In order to use our time efficiently and to not waste your time as well, since we also have other questions, lets go on with our discussion on the pictures. The third photo that we have chosen is this one. What stands out for you in this picture?

R: So here, she *(daughter)* won't let anybody else unbraid her hair except for me. Not even her mother. She *(the mother)* braids it for her *(daughter)* but she will never let her unbraid it for her. She says that it hurts when her mother does it. So first I gave her a notebook and a pencil so that she can draw, the child beside her is the neighbor's kid and I am telling her to draw something and show it to him. While she is distracted with this, I am unbraiding her hair. She is never willing otherwise. She doesn't let anyone touch her hair.

I: Earlier you told us that she misses you since she spends the entire day with her mother and you leave for work. That is why she prefers that you feed her, that she falls asleep when she is with you and that she only lets you unbraid her hair. So, what kind of feelings does this spark in you? Or how does this influence your responsibility and role in taking care of her *(daughter)*?

R: This makes me very happy. I maybe angry or sad during the day when I am away from home but as soon as I get home, believe it or not, I will forget everything. Everything. When I listen to her talk, it makes me forget all things. Then I sleep. Someone told me, "you should not regret what you did at 7:00 when its 7:10". I was told this around 10 years ago. Sometimes, when you are angry at someone and you keep thinking about it over and over again, you just waste the rest of your time. During the day, many things can happen, your work might not go as planned and things might go wrong, and you might end up with nothing to take home to your kids. There was a time I didn't even have money for transportation when I came here around CMC. For transportation. I then went to the train ticket office and begged the ticketer for a ticket and used that to go to Tor Hailoch. What I believed when I initially came was that I will be successful. But I wasn't angry. Why? The ticketer even asked me if I travelled additional distance after taking the train and I told her no, I only have a short walking-distance to go after that. She asked if she could give me money for a Taxi. Her kindness made me forget about what had happened. She only gave me a 4-birr ticket for free but I completely forgot everything that happened. In that moment, the lesson I took was if you let things go, you will end up with something even better.

I: How about in the society, I keep asking you this question so that we won't forget, this photo shows us your perspective only. In relation with the society or with other people around you, what can you say about this photo?

R: *(Pause)*

I: For instance, you are unbraiding her *(daughter)* hair in this picture. Other fathers, in taking care of their daughter's hair or in any other way, how would you describe their involvement in this?

R: Again, it depends on each person's personality. Some say "if her mother is around, why are you doing it?". Some have this way of thinking because braiding hair, washing clothes, doing hair, are female roles. If I knew how to braid hair, then I would happily braid her hair. I don't know how to braid hair yet so I comb it for her. If she wants it tied in a ponytail, then I do that for her. But if it's braiding, then I can't do it. She even says it is painful when her mother combs her hair so I do it slowly while I distract her by playing with her. When you go to the community, I don't know how to describe it.

I: it's alright. Let's go to the next photo. The photo we chose next is this one. What do you see in this photo?

R: In this picture, I am trying to teach her how to eat by herself. Instead of us feeding her. What we did was, her brother is sitting beside her on the other hand and he has his own plate as well. Her mother and I are also there. By the way, we usually all eat together on a large common plate. But here, I thought it is about time for her to start eating by herself so we all took individual plates and tried to get her to eat by saying who is going to finish first. We made it a competition so she hurried up and finished eating. So here, she is almost finished eating and she is watching TV. She is almost done. She is picky sometimes, she likes potatoes, vegetables and tomatoes. The reason I did this was thinking about the future. If her mother or I aren't around and her brother gave her food but couldn't feed her, then she should be able to feed herself. Now in the competitive spirit, she is happy thinking that she is winning, once she ate. We talked about it earlier so her mother and I are intentionally trying to lose to her.

I: So that she eats by herself?

R: So that she eats by herself.

I: Let me take you back on your previous point when you said that you all usually eat together in your household. Can you please elaborate on that? Why do you do that? Are there benefits to it? Why did you choose it?

R: It is just so that we eat while enjoying each other's company. There is no particular reason. *(pause).* I don't know.

I: It is just something you frequent?

R: It is once I came to Addis Ababa that I ate on individualized plates. Where I grew up, even at weddings or parties, you eat together on a large plate. That is how it is served. Everyone is equal, rich or poor, they all have equal contribution. From what I observed, there is around 40% wastage when you eat on individualized plates. Apart from a few people who only take out the amount that they can properly finish, most people fill up their plates way beyond what they can eat. The amount wasted is excessive, even at memorials. It may be easy to serve and accommodate people but when you take into consideration the amount of wastage, it is not advantageous. When it comes to our home, there is no additional wastage even if we eat on separate plates but the main reason why we eat together on the larger plate is so that she *(daughter)* eats with a competitive spirit. By competing with us.

I: So that you spend time together?

R: When we eat together, she properly washes her hands before she starts, she sits, and she starts to eat competitively. If I want to make her eat, or if her mother wants to make her eat, and for example if we have "Misir Wot[6]" and "Shiro Wot[7]", and she sees her mother eating from both, then she will do exactly what her mother did and eat from both. If we want her to eat well. If her mother only eats "Shiro", then

[6] Misir Wot: Lentil Stew

[7]

she will do just that. So, these are the things that we have done so that she eats properly and can later be able to feed herself.

I: When you said feed herself, I am reminded of a question. What things have you done so that she will be able to feed herself? Are the things you did just so she can be able to eat by herself or what additional things have you done to help her do things herself?

R: The other thing I do so that she can do things by herself is I buy her different toys for her to play with. The toys I buy her are made for girls and they contain pots, spoons, forks, and so on. In addition, while her mother is washing clothes, we give her a smaller wash basin so that she can wash her own stuff. When her mom is about to make coffee and she prepares the materials she needs, she *(daughter)* does the same with her toys and follows her mother. So, it is not only so that she can be able to feed herself, it is so that she can also do other things by herself. All in all, she follows and does everything that her mother does. When her mother washes clothes, she washes her doll.

I: What things are there that she learns from you? Since you told us about the things she learns from her mother. How about from your side?

R: From my side, there isn't a lot unless there are some chores that I help with, in the evening when I get home from work. As soon as I get home, I take my shoes off, wash up and sit mostly. Occasionally, if there are things that I must do, for instance if my wife is not feeling well or if she has gone to church, then I do what she *(daughter)* needs. If I need to wash her feet or face, I do that. After that, if we must eat dinner, we wait for her mother to come home and eat together. Apart from this, it is mostly her mother that she follows. My wife mostly takes care of the housework since she stays at home.

I: You have raised the issue on toys so do you buy similar toys for your son, the one with pots?

R: No, when he was young, he has broken a lot of my mobile phones. He is more interested in phones.

I: How old is he now?

R: He is 11 years old now. He has broken 3 or 4 of my phones. Previously the ones that were available were the Nokia flip phones and the Sony Ericson but as the technology progressed to smart phones, he also grew up and he now listens when you tell him to stop. When he was smaller, he used to like guitars and toy guns to play with. This is the society's value, toy guns are for males, not for females. Toy pots and spoons are only given to females, not males. There are sayings like this. But in my case, it is a coincidence that I have bought these toys for her and not for him.

I: What is your view on these sayings?

R: I just take them as sayings. You know why? A female has saved me from an attack previously. On our way back from Jigjiga, I don't know if you know the place, it's called Karamara.

I: I know it.

R: Before we reached Babile, there were robbers/pirates who blocked the roads so that they could steal from us. We were traveling at night. They have lined the road up with stones and blocked it. The spoke in Somali language and forced us out of the car. The woman, she told us to just keep quiet and sit still. We were confused about what she was going to do so we just sat. We were confused. She got out of the car, I don't know what happened but when she came back, she brought the robber, firmly tied up. We then

realized that only one of the robbers/pirates was armed, the others were not. She then ordered them to remove the stones from the road. We were extremely confused. It was the first time that I saw robbers/pirates like this. We later inquired about what happened. As it turns out, she is an Air-Force trainer. Apparently, she used her own methods to handle the situation. Later she loaded them into the car, and we continued our journey to Babile. When we got there, she handed them to the Babile Police and had them registered. It took us 3 days to get out of Harrar because we were so scared of her. It was my first time going on a field trip. It was also my first time with such experience. So, I thought, being a hero or being armed is not only for males, there are also female heroes (heroines). There are heroines who have defeated males. That is when I understood that those things are just sayings. Well, it is nature. Our fathers used to say this, females should always walk in front of or behind their husbands, not walk by his side. There are lots of sayings that state that females should walk behind their husbands. If you ask why, beyond it being a saying, there is nothing more. Why can't she walk in front of her husband is a question I frequently think of. For instance, in my surrounding, this guy, he is married, and he doesn't give any value or respect to his wife. It may be because he only wants her to bear children, wash his clothes and things like that. He always argues with her. I ask him questions like why they always argue or what did she do to you. He sometimes even hits her. We then ask him why he hits her. He says, what else do you expect me to do if she is rude or disrespectful. There are even such beliefs in the 21st century. I always argue with him about this issue. The ideology that you never buy toy pots for males and toy guns for females is very outdated. The reason I didn't buy these things for my son and that I have done so for my daughter is because I am better off now than I was when my son was born. I had a job and I earned some money. It is purely coincidental, not because of preferential treatment. However, in the society, this ideology is still accepted. There are some who say, how could females go to bars and drink alcohol. It always amazes me. Before the conflict, I went to Mekelle. Many Eritreans were there in Mekelle and they were extremely surprised when they saw other females eating raw meat. They were extremely surprised. They were shocked at how a female could eat raw meat like a man. I stepped closer and asked them further. They said that it is a disgrace in their culture. By the way, where I grew up, buying Injera from a shop, was very frowned upon. You only buy bread from the shop.

I: Why was it frowned upon?

R: It just is. They would say, is she really a woman if she goes out to buy the Injera instead of making it at home for her family. They would say, no, she is not really a woman. Previously, if you get into a relationship, and your partner puts his arm around you while you are walking together, then it is extremely frowned up on and heavily disapproved. But now, they even kiss on the streets. Right on the streets. When I was young, we used to make fun of people who chew Khat by saying are they chewing "Gesho[8]". There were only two people in the city who did so *(chew Khat).* But now, from young to old, the Sheikhs, the Priests too also chew Khat.

I: A lot changes with time.

R: Yes, a lot changes with time. Do you have such ideologies or not? I personally take the realistic approach, not the flexible one. I wouldn't let the thought that something was there yesterday but no longer today stop me from doing what I think is right.

---

[8] Gesho: Rhamnus prinoides, the shiny-leaf buckthorn, is an African shrub or small tree in the family Rhamnaceae. Commonly referred to as "gesho.

I: May be when you said this, you reminded me of our previous discussion. We asked if you take role in somethings inside your home, they could be things that you have mutually agreed on without even discussing it. Are there roles and responsibilities that you believe are yours and those that are your wife's? For example, if she went to church and it got late, or if she is unwell, you said that you take some roles, in serving food and others. Adding onto this, are there roles that are mostly yours or mostly hers *(wife)*?

R: No. To your surprise, mostly, especially around the holidays, making "Doro Wot" is considered a female's role. By the way, I am the one that always buys the groceries. For instance, this past Monday, I went to Haile Garment for a job with my friend and we swung by the market there. I know the price of the vegetables around my area, onions are 14 birr per kilo and tomatoes are 17 birr per kilo. When I asked there *(market at Haile Garment),* the price is half that. Onions were 7 birr per kilo. So, without asking my wife, I bought what I thought was needed, tomato and other vegetables. In the evening when I get home, my wife may take my daughter with her, to church or anywhere, my son stays home, I immediately check if there is food ("Wot"). For example, if she *(wife)* has spent the entire day washing clothes and she went to church in the evening, she might not have time to make food. I check, if we have "Wot", then I reheat it, or make Shiro. Apart from that, there is no distinction between my role and hers *(wife)*. For example, if I spend the entire day at home thinking that I shouldn't go out today, if she is making Injera, then I wash the dishes. If she is washing clothes, then I make food and coffee. I am not telling you about the things that I don't do, I am talking about things I actually do. There are people who say no, this is a female role, not a male role. But primarily it is for my own benefit since my wife is my better half. My children are also parts of me.

I: When you said that you buy groceries, you have told us previously, since you know the market prices, you participate in this activity and that you buy things when you get a better price. Usually, in most of the society, grocery shopping is done monthly. Do you always do the monthly grocery shopping or is it your wife? Or when you said that grocery shopping is your role, do you mean to say that you provide the money or that you also do the physical shopping?

R: We don't buy groceries monthly in our household. It depends on our consumption. For example, I am not the one who buys Teff, I just give her the money and she goes to buy it. I do this because I believe that she knows better and she will choose the right one, not because I think it's a female role. I once bought Teff, but it wasn't of good quality, so we returned it, it was not edible. Since then, we found it best that she does it. Females have a relatively higher capacity at identifying quality, especially when it comes to food. We don't buy anything monthly. We just buy what we need when we run out of it. So my role, I observe when we run out of onions or potatoes, so if the circumstances allow me, like I have told you earlier, then I go and buy everything we need, within the limits of my capacity. Be it 5kilos or 1 kilo or 2 kilos. If the circumstances do not allow though, because there are shops within our area, then she *(wife)* might be the one that buys those things. Otherwise, we don't usually buy anything periodically.

I: Okay. We are having a great discussion. The time is also flying so we will hurry it up a bit. This is the next photo. Tell me what you see here.

R: This shows her *(daughter)* playing with her toys. This is our corridor. *(Shy laugh).* There is a house on one side, ours is in the middle, and there is another house on the other side. She is playing with toys. This toy wasn't hers; it was my son's. It is a computer game that reads the alphabets.

I: Okay.

R: She is playing with that.

I: Okay. So as usual, what do you think, in relation to the society, how do you see this photo? How do you describe it in relation with the society's way of living?

R: Directly in front of us, the toys they have at their house are numerous. They have building blocks and others that their kids play with. She *(daughter)* goes there and spends the day playing with them sometimes. But most of the time, they *(neighbors)* work. It is usually only the mother and daughter that stay home. The males usually come in the evenings and I don't have detailed information as to what they do when they come home. Here my daughter is wearing her mother's slippers. If you can see what she is holding in her left hand, she is holding something that you place a pan on, like a stove. She is telling me about the things that she did. The other thing she is holding is the handle of an umbrella. In a movie, on "Kana[9]", I don't know if you know it, there is a character that uses a cane, for support. On Kana TV. She imitates him *(the character)* and makes people laugh. There is also a show about a Captain, maybe Captain Ali, that airs at 8pm. She says Captain Ali, imitates him and makes them laugh. She makes fun. For instance, she surprised me so much yesterday by writing the letter A on the computer game. I usually show her, this is A, this is B, and this is C.

I: Is she enrolled in school?

R: No, she is not enrolled in school. Yesterday, she came to me saying I wrote the letter A. I was so happy watching her write. It is on the keyboard, which ones A, B, and C are.

I: Why hasn't she been enrolled in school?

R: Because she isn't 4 years old yet.

I: You are waiting for her to become 4 years old?

R: When she turns 4, with God's blessing, *(Pause)*

I: She will be enrolled?

R: Yes.

I: Okay. Let's go to the final photo and finalize on our discussion points. This is the last photo. What do you have to say about this?

R: I came home around 5pm or 4:30pm. I occasionally suffer from indigestion when I eat late at night, so we ate early, at that time. Her mother and brother are not in the house. Her *(daughter)* and I, are following them. We are going to a place called "Eyesus Gedam".

I: Is it a church?

R: Yes. She is usually very happy after she comes home from church. She is even happy talking about what she learned and what she saw at church. Let me tell you what she did one time. She went closer to the priest to get blessing, but he didn't see her, so he kept blessing the adults only. She was very angry, so I asked her what was wrong, then she told me that the priest is only blessing the adults, and that she didn't get her turn, so she wanted me to tell him. I then asked him *(the priest),* to please bless her, he was

[9] Kana: Popular TV channel in Ethiopia

apologetic and proceeded to bless her. I was amazed at her. She later asked why they only bless the adults and not her, and I told her it is because she is a child. I told her that since she doesn't eat well, or sleep properly, that he wasn't able to see her. I added, we eat and sleep well so we grew up and that is why he can see us and not her. This sparked her interest in eating well. Sometimes she asks very difficult questions. When she hears people using insult words, it chews her up inside. She thinks about it a lot then she tells me what she heard. She even tells me if she sees her brother sitting with the person, the person who she heard using the insults. I tell her if she uses these insults, that "Amen Amen" will be disappointed. She refers to God as "Amen Amen". Even when she does something else, if I tell her that God will be disappointed, she immediately stops. I am happy that she is God fearing even at this young age. Even the names of my children are unique. My son's name is______.

I: Who?

R: ______ People are constantly amazed. Everyone asks us what it means. Only people who have an in-depth knowledge of the bible know the answer. Her name is _____.

I: Do you often go to church together?

R: Yes, but not continuously. Occasionally. Depending on the amount of time that I have. If I get home early, then we will have our dinner early, go for a walk, and if she gets hungry when we get back, she will be given something to eat, like bread and tea. We then sleep.

I: Where else do you go together?

R: Not that much. They used to go to recreational places, before Corona, not with me but with my sister, the one around 4 Kilo. She used to take them to some places, along with her child. Places like Edna Mall. This was before Corona, since then, we don't go out of the house. In our area, there is a small park built for kids, but it is rarely open. Even when it is open, it is not affordable, it is very expensive. If you want to take a kid to Ghion, for example, then you will spend a minimum of 100birr, for him to play on 3 or 4 things.

I: I just wanted to know if you spend time together in other places, aside from home and church. That is why I asked. Okay.

R: I usually don't take my daughter, but we go out to watch football matches with my son. He likes football. Since we don't have the channels at home, we usually go out to watch the matches. Once a week, on Saturday or Sunday, if there is a good match we go together. For example, last week there was a game between Arsenal and Tottenham, we watched that together. We watched it at a public club while he was drinking a soda and I was having draft beer.

I: This is good. You are giving us detailed information. That is why we are taking a longer time. We are getting good input from you. We have discussed all the above based on the photos. So, may be if you want to add something? If not, we will raise some following discussion points. For the questions that I am going to ask you next, keep in mind the photos we have already discussed, the 6 photos, and we will discuss.

R: Good.

I: I have already asked you a few questions on your perspective from the society. Now I am going to raise a slightly different question, from the questions I asked previously. So, in the area you live in, how would

you describe manhood or masculinity? For example, you can tell me what qualities someone must possess or how should someone act in order to be considered a man. Describe it to me.

R: Manhood, firstly, is what someone is given naturally. Male, female, both are naturally given. I don't believe that physical confrontations or fights aren't what make someone a man. He has to have his own personality.

I: Yes, that is your personal perspective. But in the society, what defines a man? You have told us some things earlier like people saying how could a female go out and eat raw meat like a man and you told us that is not how it should be defined. Tell us the society' definition for masculinity, beyond your own personal perspective.

R: Okay. So, what I have frequently heard people saying are how could a man go home so early, a man must drink, a man must get drunk and so on. I, for instance, fetch water, if we don't have enough at home. During that time, most people think, how could he do that when this is clearly a female's role. They even ask me where she went and why I am there instead of her. When it comes to roles like washing clothes and cooking food, the society has assigned these to females. Being a man means drinking, getting drunk and stumbling to his house, being insolent, and being loud, to some members of the society.

I: On the other hand, how about the positive aspects? For example, you raised a point about being a hero earlier. You stated that heroes are not only males. This means that the society portrays men as heroes. So please add on this? What is meant by a man in the society?

R: A man is the head of the house; he works hard and earns money that he gives to his wife. There are some that believe that females cannot be heads of houses, that only males should have jobs, should earn money, and should support his wife. Accepting money from his wife is considered a sign of weakness. Some members of the society feel that males are heads of households, but females are just seen as household goods. This is an outdated but still rampant way of thought. It was just passed down from the 19th century and should no longer be in existence in the 21st century. There are a few things that are given to men. For example, slaughtering a sheep is a duty given to men. This was given by God. So, it is inappropriate to ask why females cannot slaughter sheep. There are also roles given to females only. Bearing a child is a blessing given to females only.

I: I just want you to elaborate on this a little more. Bearing a child, like you said, is clear. It is a natural blessing since only females have a uterus so they could get pregnant and have children. Men naturally don't have this. However, when you said that slaughtering a sheep is a man's role and females can't do it, it might not be clear to everyone. Are you seeing it from a religious perspective or from the perspective of physical strength? From which standpoint have you seen this?

R: It is not from the perspective of physical strength that I have seen this. Let alone sheep, there are females who have the strength to tip-over an ox. I looked at this from a religious angle. Most of the time, it is males that perform the blessing. It is purely from a religious angle. From a strength angle, I have told you the story about how one female defeated 3 males while we sat scared in the car. Strength doesn't just depend on physical appearance.

I: It is clear now, thank you. Okay. I think you have described manhood very well. If you don't have additional points, you have given us various good examples on this so are there other things that you don't think are acceptable? Personally, for you. Among the sets of values that the society has placed for

males and females, are there values that you think are wrong, or values that you think we should follow? How would you describe this to us?

R: Among the things that I don't accept is the belief that household chores are only for females. Like I told you earlier, I help around the house for my own sake too, not for someone else. Your children and your wife are parts of you, they are not external entities. This means that I do it for myself. So, if I am doing it for myself, it doesn't work if I say that she *(wife)* should be the only one to do the chores. Secondly, I consider it to be coincidental if a male is the one earning the money. There are situations in which the man is staying home, taking care of the kids, washing clothes, and doing everything else while his wife works to make a living. It is not because he is a male or she a female. As a matter of coincidence, if she is educated and has a work opportunity, but he couldn't get a job opportunity with his line of study, the fact that he is staying home and taking care of his kids is not a sign of inferiority. This is one of the things I don't support. I don't accept that it is only a man's role to be the head of the household since there are females who are heads of their households too. Let alone their own homes, I know females who even lead the households of their neighbors. They could be role models for many others. So, I don't accept that being the head of the household defines masculinity. Secondly, there are some people, he may take his wife out for recreation, he orders a beer for himself and orders a soda for her. He doesn't even ask her what she wants. He assumes that because she is a female, she shouldn't drink beer. What is the harm if she drinks beer? How about the other way around, he drinks soda while she has a beer? These things should be according to her preference. If we go out for example, my wife will have whatever she prefers. The only factor is how much money I have. If I have 200 Birr in my pocket, then we will enjoy ourselves within that limit. I ask her what she wants. If I only have 200 Birr in my pocket but my wife wants to have Whisky, then I ask how much it is. I am telling you what I am going to do. Similarly, if we go to a big hotel to have lunch, after we have chosen what we are going to order, I ask the price and think of how much I have in my pocket. For example, if one food item on the menu is 60 Birr and we order 2, that would be 120 Birr. If 1 draft beer is 15 Birr and we have 2 each, that is 60 Birr. The total is 180 Birr. And then I think of buying 1 soda for my kids. This is the calculation I do before I order anything. One cannot criticize me for doing this because I am acting within the limits of my financial capacity. I don't take contract taxis. For instance, I would not take a Ride[10] taxi from here to Abinet with only 100 Birr in my pocket. Because I know how far 100 Birr takes me. So alternatively, I take the train. I reject ideologies that correlate manhood with money, education level, and physique. Manhood is primarily about mentality. There are females who have money and knowledge so we cannot call them men simply because they possess these qualities. This is how I look at things. So, there are things within the society that still require a lot of work.

I: Okay. How do you describe fatherhood? Not you specifically, but how is it described within the society? It might be similar with your personal answer. You have already described manhood and masculinity. What is the view on fatherhood? What qualities does the society think that a father should have?

R: There might be various views within the society. But fatherhood for me, beyond anything, it is a source of happiness. This is because there are many who were not fortunate enough to become fathers, despite their wishes, people I know. Being a father makes me happy, more than anything. I cannot tell you how happy it makes me to walk while holding each of my kids' hands on each side. I truly feel happy within.

[10] Ride: Equivalent of Uber that works in Addis Ababa

But within the society, even though I don't understand their way of thinking, there are fathers who are even scared to list themselves as their kid's father on official papers, at schools or at Kebeles.

I: But how is it described? Is there a set of characteristics for the society's definition of fathers? What are these things?

R: If you take me as an example, I hate to see any child get hurt. Even in the streets, if I see a child with a runny nose, I will find a tissue paper and clean him/her up. My father raised me to be brave. He raised me in a way that I wouldn't be scared of anything. But he also made sure that I wasn't rude or insolent. I was raised to be God fearing. I am the youngest child. He is a priest and he used to take me to church when he used to go early in the morning. I used to sleep by his feet. Then in the morning, I would go home with him after the church processions. So even when I go out at night, I am never afraid. If you raise a child tied up even with a tiny thread, you can still control him using that thread even after he has grown. But if you try to control him once he is grown, even a huge chain will not stop him. It all depends on how someone is raised. That is what I think. I never insult anyone or say anything rude. It angers me when people insult their kids. I think if you are like this for your own child, how are you going to be to others.

I: That is good. I am going to ask you the same question again so that everything will be clear. For example, if someone who is not from this community or who is from a different society asks you how you would describe fatherhood in your society, country or area, how would you describe it? How would you say fatherhood, in my society, from my way of living, according to my neighbors, where I live, is something that fulfills a set of criteria? What are those criteria?

R: Fatherhood primarily means sacrificing for your kids. He should always put his children first.

I: Sacrificing and giving priority? Okay.

R: For instance, I may have only one shoe and I will be just fine. But kids, naturally, even want new shoes if they see the neighbor's kid wearing new shoes. So, one needs to prioritize his kids when it comes to things like this. It requires doing what your kids want in order to make them happy. Your kids' happiness will in turn make you happy. People cannot tell how you feel but when they see how happy your kids are, they immediately say that you must be an amazing father if you can give your kids such joy. On the other hand, if they see rude and unmannered kids, they immediately think, what kind of parent raised them. This is what I understand.

I: So, can you say that fatherhood can be portrayed in his kid's behavior?

R: Yes, it can be portrayed by his children, his society, his social life, and his entire surrounding. Because if one always comes home drunk, people will be disappointed in his/her parents.

I: Okay, let's proceed. We have seen various photos. Can you summarize and describe your role in your young children's nutrition and feeding habits?

R: With whatever amount of resources I have, for instance, if I have 100 Birr, I try my best to take home something for my children. It may be bread or bananas. I only save some money for my transportation for the next day. Otherwise, I take home whatever I can afford. I can only feel satisfied and fall asleep once my kids are happy. To your surprise, I was having money problems recently and I had difficulties paying the school fee for my son. As I told you earlier, once I stopped working, I was just using what I had in my savings. There wasn't a lot of work due to the pandemic. Once he (son) was registered, they skipped the

first two months and asked that we pay for the total of 3 months. I just paid for the third month, without paying for the first two months. When I went to pay for the fourth month, they gave my son a letter from school. He was deeply saddened by the letter. He asked me if I didn't pay for his school because I didn't have any money. He then said, we can just use the money that I (son) wanted to use to buy clothes to pay the school fee. I was amazed. I realized that he is the source of my happiness. I realized that the source of my happiness is my son. Thank goodness, that all passed. Coincidentally, my sister, who lives in Assosa, was incarcerated at the time and she was not able to help me. She was released after 3 months and that is when she sent me some money and I took care of all the fees. This is what I can say.

I: Are there things that you wish you could do? Maybe things that you think you should improve on? As a father, to take care of your kids and improve upon their nutrition.

R: Fulfilling all their desires would make me very happy. But in the current situation, with everything being so expensive, it is impossible to fulfil all of one's desires. Human beings have limitless desires. For example, one who wishes to have a 1 room house and you give him that, then he would want a bigger one on the third day. After 3 months if he gets married, then he would want 3 rooms. When he has children, he will want 4 rooms. This shows how our needs and wants are limitless. My only wish is that my kids remain healthy. I will work diligently and strive to make them happy. Kids are easy to make happy. For example, my daughter would be happy even if I buy her chips. Chips. I can not tell you how happy it makes her when I even buy her biscuits. Making her happy makes me deeply satisfied. For whatever we have until now, I am grateful to God. Even if their desires are endless…

I: Within the limit of your capacity?

R: Within the limit of my capacity, I do whatever I can for them.

I: In the future, what improvements do you think will be best for you to be able to do more for your children?

R: I want to have a permanent job. If I had a permanent job, then I would be able to provide in a better way, for my children. She (daughter) will enroll in school next year. I plan to transfer my son into a government school and enroll her in a private school.

I: Okay. So, you have told us about your role. You have shown us pictures that support what you said. In order to summarize it well, can you tell me your main roles and responsibilities in the raising and feeding of your children?

R: It is raising them in a loving and supportive environment. That is the major thing. It is not about attempting to be rich to give them a big inheritance. It is about giving them love, as a father should. I also want them to be God fearing.

I: How about your responsibilities?

R: My responsibilities are the following. One, teaching them not to be insolent and rude. Taking care of them, explaining things to them, making sure that they are not brash but bold, that they are not brash or shy. The reason why I don't want them to be shy is because I don't want them to tremble and stutter when they are facing other people. I also don't want them to be arrogant. I just want then to be God fearing. As long as I shall live, I just want to see them well behaved and good mannered.

I: You have mainly told me about your roles and responsibilities in shaping your kids behavior, giving them love, support, and making sure they are God fearing. What is your responsibility in your children's feeding practice and nutrition?

R: It depends on my financial capacity. For example, making sure they eat vegetables. For instance, she (daughter) likes eating fruits. My son, because he had repeated tonsillitis attacks when he was a child, he has just recently started liking fruits. To your surprise, she eats black pepper because she believes it will protect her from tonsillitis. Not only this but regarding their overall nutrition, I believe that they need vegetables and other balanced foods. So, I will raise my children to the best of my capacity, with God's blessing.

I: When you say depending on my capacity, are you referring to your ability to provide for them and to buy them what they like to eat?

R: Yes. Within my capacity, I think of the things they like, be it vegetables or fruits. What other foods do they like? Pasta? Macaroni? To your surprise, she (daughter) really likes watermelon. She loves watermelons. I cut it up for her and keep it in the fridge for her to eat whenever she wants. And many other things. I may also buy pineapples, mango, and so on, it all depends on my financial capacity.

I: So, this means that you believe that your main responsibility is providing food?

R: Yes. In addition, following on their feeding habit and nutrition. Things like how is she eating and is she eating well? After work, with the time I have, I do that. Her mother is also good at following these things. I also follow whether she has eaten or not. I ask and observe.

I: We are majorly done with our discussion. We are only left with one question. You know that we are amid a pandemic and as you have repeatedly mentioned, there are various changes that have come about since then on people's lives. In association with that, what changes have come about in your or your family's lives? After the pandemic, what changes are there in your social and economic lives and so on?

R: Economically, it has impacted us a great deal. There are no jobs, you spend most of your days at home. We eat whatever we have at the house. Then progressively you use up your savings. The pandemic has not impacted our household only, it is a national and global issue. However, the time since the pandemic has helped me spend time with my daughter and that has strengthened our bond and connection because we are both at home 24 hours a day. My son too has gained over 10 kilos. Just because he is staying home. When he goes outside, he loves football, so he is always playing. My daughter plays with him too. Since he has started staying at home, he has gained weight. On one hand, we are heavily impacted economically but it is national and global. On the other hand, it has improved my communication with my children. We talk about various things and watch movies together. Sometimes we even stay up after midnight talking and we sleep during the day. We also help each other with house chores, no role is assigned to one person. For instance, if I wake up earlier than the rest, I make breakfast and we eat after they wake up. After that we continue enjoying each other's company, watch movies and so on. Occasionally I go out, taking all the safety measures. I bring movies on a flash-drive and we watch them. But economically, the impact is huge, although it is not something I am facing alone, it is global. Economically it has devastated me, together the political conflict and other issues. By the way, I am Tigre. I was born and raised in Adwa, Tigray. It is where I went to elementary school. I continued high school here in Addis Ababa. It has been 23/24 years

since I came to Addis Ababa. When things like this happen, I know that I am not alone, so it doesn't sadden me that much. If it was something I faced alone, if it was personal, then it would have been heart-breaking.

I: So, the thing that happened to you is not different from what is happening to everyone else?

R: Yes, because it is a national and global issue.

I: Okay. _______, thank you for the discussion we had. Maybe if there is anything you might add or if there is something that we haven't raised, in association with the study, I will give you a chance to add or raise your point. Otherwise we will finalize our discussion here. Is there anything you want to say?

R: The thing I can finally say is that fatherhood is a blessing. It is being lucky. Motherhood is also a blessing. There are many who wished to become a mother or father but have not been fortunate enough. There are multimillionaires who haven't been able to become fathers or mothers. I am not a multimillionaire, but I have become a father, and it is such a blessing. This makes me extremely happy. My children are my assets. They are my luck. They are blessings that I was given from god. Therefore, fatherhood is something worth respecting.

I: Okay. Thank you. We had a very good discussion. Thank you for accepting our appointments, both on the first and second day, and for your willingness to participate in our study. We have now finished our discussion.

Father 3

Interviewer: Thank you for agreeing to take part in this interview. Since you are here with the photos you have taken, we will start from those. This is the first picture that you have chosen so please tell us what catches your attention when you look at this picture.

Respondent: What catches my attention is my child, my youngest child. He is sitting in the compound. I usually like it when kids sit in green gardens, so that is why I took the picture.

I: Do you have a green garden like this in your compound?

R: Yes, I have planted these around the gate. I like greenery since they also give oxygen. I took the picture while he was sitting there.

I: How old is your child?

R: He is 4 and a half years old.

I: Why did you choose this picture in particular? What do you want to show here? In addition to the greenery, what do you want to show?

R: Although it does not show a lot, how this picture was taken just makes me feel joy so that is why I chose it. There is no other specific reason.

I: How do you relate what we see in this picture to your lifestyle or the lifestyle of the society?

R: I was raised in the countryside, so we did not have the opportunity to enjoy clean and green environments like this. In doing this, I believe that I am giving my children a better opportunity than what I was given. I would be happier if I could provide an even better environment for them, but I believe this is good for them too.

I: You thought this was better from the way you were raised?

R: Yes, I believe that it is better.

I: How would you describe your way of living as compared to the society you live in?

R: Our way of living is very challenging. It is in the countryside; we do not have access to electricity. You can not even find a place to have a decent cup of tea. Our family members live in a very difficult situation. It is impossible to imagine a bright future in this circumstance. So, there is a wide gap between the way I was raised and how my children are being raised now.

I: How about your way of living as compared to your neighbors, where you currently live with your children?

R: It is not great, but it is okay. We do not get a chance to meet that frequently and we only meet occasionally, for instance, when our children quarrel. During those instances, I have seen that our ways of

living are different. Back in the countryside, if someone orders you to do something and you do not do it, you will be punished. But here, children are in school during the day and are only home for a short hour. It is totally different from the countryside.

I: Is it only the availability of water and electricity that makes it different from the countryside or is there anything additional that you want to mention?

R: It is a lot of things. Even for your cognitive development, you see a variety of interesting things, be it on television or elsewhere. In the countryside, when I compare it to here, there is nothing interesting to see or partake in.

I: It is different?

R: Yes, it is different.

I: So, what you are saying is that this picture shows how differently my children are being raised as compared to how I was raised myself?

R: Yes, that is what I am saying.

I: Let us proceed to the next photo. What about this one? What stands out to you on this picture?

R: What I see in this picture is, I am doing some household chores. We do not have a maid, so I am helping my wife with some laundry. So, this picture shows that he is trying to help me sweep away the water from the ground. What I learned from this is how much he follows in my footsteps and tries to copy my behavior. For example, cooperation and working together in the household, is what he is learning from me. I work both as a male and as a female within my home. I help in giving the children bathes and washing clothes. My children are copying my behavior. He is helping me sweep away the water here. We used to help our mother when we were raised so this is a good example for them. In line with your work, I took this picture because I thought it would be useful.

I: You have mentioned that there are no gender specific roles with your wife in your household so please tell me how you assist her in the household chores? Please elaborate on that.

R: I do not believe that there are specific male or female roles in the house. The only thing I can not do is make Injera[1]. I can not make Injera. Household chores, this is not to brag, but I do everything. I wash clothes, bath the children, wash my daughters' hair, I have young daughters, changing the children's clothes and shoes, everything. Since she (wife) can not handle everything herself, she is hypertensive, it will be a burden on her. If something happens to her (wife), it is my loss too, so I do most of the household chores.

I: How about in their feeding? Do you participate in that? By feeding your children or it might even be by buying the food that they eat. Please tell me your contributions in that.

---

[1] Injera: Injera is fermented Ethiopian ethnic traditional staple food prepared usually form teff flour.

R: Next to God, I am the provider for the family. I work in the evenings, and I try to get everything that they need for their breakfast and to cover their needs overall. So, I have a vast contribution in this regard. Even when I portion their food, they might fight by comparing the sizes, but I encourage them to share and eat together lovingly, no matter how little what they have is. Eating together will enhance the love they have for each other. So, we never portion their food individually, we encourage them to eat together to avoid competition and to enhance their love and kindness towards each other.

I: Are you in the house when they eat their breakfast, lunch or dinner or is it always your wife that is around?

R: Mostly it is my wife that is around since she is mostly home. I am around occasionally. If we are all home, we eat together on one plate. It is only if we have guests that we will eat separately. When I compare it to how I was raised, eating together with the children on one plate enhances our sense of unity and togetherness. It gives them unity and will increase their care for each other. If they eat separately, you have no way of knowing which one of them has eaten adequately or not but if they eat together, you can be able to identify their needs. Although I am not capable of providing for all their needs, for example, sometimes, we might be thinking about making Firfir[2] but what they want might be eggs, but we try our best to make them enjoy what we have at hand.

I: So, you are involved both in providing and preparing the food that they eat?

R: Yes.

I: It is because you said that you can cook.

R: Yes, I do. Because I must, if their mother is not there, then who will give them food? So, it is one of my duties. Even if all you do is heat up what has already been prepared and give it to them, it is still good. Tea and other simple things. Serving what has already been prepared, feeding them, and kids are happier when you feed them.

I: Let us move to the next photo. How about this photo, what stands out to you? How would you describe fatherhood?

R: Fatherhood for me, I have three children, being a father is difficult. For me, I believe that being a father is a blessing. it is a huge blessing. I think that if I were not married and if I did not have my children, my life would have been ruined. Looking back to my rebellious days and comparing it to what I have now, I am much happier now with my children. Being a father is a blessing. It makes you happy, only if you understand it well. As per my understanding, being a father is great. My children are my wealth. If I can shape a generation well, not in the way I was raised but my providing them something better. I am happy. In the house we currently live in, we moved here recently, we have recurrent problems with flooding and drainage and at one time my children were not home for about one month and we suffered due to the distance. After that, glory to God, we managed to fix the problems with the house. We did not have any

[2] Firfir: Ethiopian food, typically served as breakfast. It is served by preparing sauce and shredding injera into pieces and mixing the two.

chairs previously and comparing that with the current situation, just sitting together is a part of God's blessing since it is beyond my capacity. Being together. For other people too, even though they face challenges and adversity, there will be a way to overcome them. If you persevere. People who persevere and are tested through adversity are good. If you come out the other end after being tested with adversity, good things will happen to you. For example, comparing it to how I was raised, fatherhood is being the best that I can for my kids. Not like me, I have some flaws, for example I am easily irritable sometimes, and after working on these flaws, I need to be the best version of myself for my children. Fathers are expected to be good to their children. So, if you understand it well, it is great. Being a father is a blessing.

I: I believe that you have described fatherhood well. But there is something I want you to elaborate on more. You have said that being a father is a blessing. From what angle have you called it a blessing? Is it because of just having offspring, forming a family, or as you described children are assets? Please explain more on that.

R: The reason I said that my children are my wealth is this. Money is expendable, people also pass. People are only able to live on to the next generations if they have children. Blessings are given from God. The blessings God has given you is your wealth. Money and property are dispensable, but children are your assets. The reason I believe this is, during your early and strong age, you can work hard and strive to provide for them and raise them, so instead of acquiring wealth at this age, raising children, and shaping the next generation is more important.

I: Thank you. We will now pass to the next photo. Please tell me what you see in this picture?

R: In this picture you can see their mother feeding them. She (wife) did not see me take the picture. The oldest one, she is 10, the one following her, she is 8 and the youngest one is 4 and a half years old. The older one wants to feed her mom (give Gursha[3]) too. If she does so (eldest daughter), the others want to do the same to compete with her so, she gives Gursha to all of them. They sometimes refuse when their mother is the one giving them the Gursha but when it is the eldest daughter, they compete and eat well. When children receive the adequate amount of mother's love and affection, they will grow up to become good citizens. So, if mothers understand the needs of their children, be it her son or daughter, and when the children are receptive of the love, that makes me happy.

I: So that is why you took the picture? Okay. How about this? What does it show? What is seen in this photo?

R: This shows the two sisters sitting together. 4 months ago, the youngest one underwent surgery for his eyes and previously he used to get teased and bullied by the other kids around the village so the eldest one repeatedly used to tell me that he (son) needed the surgery. So, we went to Minilik Hospital and there was a long waiting list and when he finally got his turn, the pandemic started. After the pandemic, they gave us another appointment and finally called us, and he had the surgery. So, the eldest daughter, suggested that I take a picture of them together since he (son) was not comfortable being photographed

---

[3] Gursha: Gursha is the practice of feeding another by placing, with one's hand, a bite of sumptuous, spicy food–wrapped in Injera–gently in the mouth of another. It is an intimate act of friendship or of love practiced in Ethiopia.

previously because he felt insecure. Not this picture though. Being together. She usually speaks out about how she feels. He (son) does not recognize it when he is being teased, because he is a child, but she (eldest daughter) notices. I took this picture while they were sitting together. He (son) did not see me, he had hid head bowed down. That is why I took it.

I: Are they eating? Is this food?

R: Yes, that is food. They are eating. He used to touch and feel with his hands previously, but he is okay now, thanks to God.

I: Is his vision okay now?

R: Yes, it is so much better now, compared to before. He used to run into objects before, but it is better now. Thank God.

I: How about this? What do you see here?

R: Here the eldest daughter is feeding her brother, giving him Gursha. This is the one who is 10 years old. You can see in her, the things that I described earlier. She is very clever. She is smart in studies too. She tries to do everything that I do. So work is relatively scarce now and so she does not want to ask for anything and stress me. She is very attentive and does not ask for a lot. So, she is hugging him and feeding him here. If her mother is not there, she tries to fill the gaps and take care of everything. She tries to do everything that I do, she considers me as a role model. If you are an understanding person, then your younger siblings will also be understanding like you. She is very decent, and she is very close with her brother. She even takes care of him after I punish him. You can consider her as his mother too. I thought this would be good for you and for other people as well.

I: So, the reason you brought it is because you think it shows how her feeding her brother shows how thoughtful she is for him?

R: Yes, it shows her love for her brother. The love she has seen from her mother and the love she is showing her brother. The things she wants for herself, she believed, are the things her brother might want as well.

I: How about this? What do you see here?

R: This is at home, in the compound. I believe this is a pile of clothes, the clothes I am going to wash on the weekend. So, in the house, like I told you earlier, if you can not afford to hire help, then you must do things yourself. The youngest one is in the house. Everything that I do is for them. If I could, I would love to take these photos and keep them for myself because I do not want my children to pass through the same things that I had to pass through. But I do not want him to be rude. My mother had to raise me in a difficult situation and so my name has a meaning in Guraghigna[4]. It means 'I will be there, after despite the harsh conditions.' If you persevere and stay strong believing that the difficult days will pass, then

[4] Guraghigna: The Gurage language is a dialect-continuum language, which belong to the Ethiosemitic South Semitic-Semitic branch of the Afroasiatic language family.

surely those days will pass. They (children) might not pass through what I have passed through but they, through hard work, if someone works hard then he will see some change. One should not forget where he came from. Like I told you earlier, my mother raised me by working in someone else's home, back in the countryside. She used to work as a maid. It is now that I understand what she went through. I, currently living in the city, always think of my mother who is in the countryside and think about what a difficult live she had led to raise us. She used to earn 2 or 3 Birr to cut grass and bring us Kocho[5]. Before I could be able to help her, I now have children of my own. From what I see now, I always think about her and how she suffered to raise me. Because I now know what it is like being a parent. Seeing how much I suffer to meet my children's needs; I compare it with my mother's. So, if you persevere, the difficult days will pass. My mom has a better life now, thank God. So, I took this picture when I compared my current life to the life my mother led to raise us.

I: So, do you make sure that your children know the importance of being hard working? Is that what we see in this photo?

R: Yes, very much. For example, if I clean the house, I want them to do it too. Hardworking people will be stronger, not brittle. You will also be able to shoulder responsibility. If you simply give them food and clothes, they would not be able to value your hard work. The reason I can understand my mother's hard work is because I experienced some of her hardships with her, in some ways. It is because I have faced that adversity. So, I took the responsibility to raise them to be good citizens. I want them to perform well in school. I, for example, spend a lot of time helping them study. But helping with household chores will not affect their performance in school, it will make them stronger. If one is strong, able to shoulder responsibilities, lead and manage a family, then he will be great. This is what I believe. Brittle people will easily crumble but a strong person, someone who can shoulder responsibilities and overcome adversity will be even braver. This is what I believe.

I: How about this? What do you see here?

R: This (long pause), was taken in the house. In association with what I told you earlier, my children go into intense stress during the rainy season. Water comes from the Gefersa[6] reservoir, and we have flooding due to that. This happened three years ago and this year too. It ruins a lot of my children's clothing. If there is flood, it ruins a lot of things. Around where we live, there is a nice house and my daughter always says that when she grows up, she is going to have a very nice house like that. Although it is difficult for me, it is not difficult for God. And so, I always tell them that God will provide us with a better house. When it rains, they always ask me if there is going to be a flood in the house. I always ask myself when we are going to be relieved from this stress. This is what I always think. So, when I get a better job, I think of a better house for my children, a better environment. I took this picture thinking about that. It is my wish and desire. It is from that perspective.

---

[5] Kocho: Kocho is a bread-like fermented food made from chopped and grated Ensete pulp. It is used as a staple in Ethiopian cuisine in place of injera.

[6] Gefersa: Gefersa reservoir is 18 km west of Addis Ababa (by road) in West Shewa Zone.

I: The house you live in now, is it the one you improved and renovated?

R: Yes, but one rainy season, the flood took down the rocks and destroyed the house. We fixed it now. We still have not tackled the problem. I aspire to get out of this house, it is due to financial constraints that we have not moved. When I see a clean and well-kept place, I always wish it were mine.

I: I will pass to the next question. In the society you live in, how is manhood or masculinity defined? What characteristics does a person need to have to be considered a man? What list of characters are listed, in your society, that describe manliness?

R: Masculinity, as per my understanding is, currently there is no division between males and females, but the things expected of a man are, primarily, he must be able to shoulder responsibility and persevere through adversity. If one just lives off his family, does not work hard, I do not consider him as a man. A man is one who overcomes adversity. It does not matter if what happens to him is bad or good, he must persevere. Secondly, he should be hardworking, self sufficient and be himself. He should not pretend to be someone that he is not. Looking at people is a trap. If I see someone dressed a certain way, I do not try to dress like them. One who lives on a budget according to his financial capacity is strong. I would say a man is strong if he can manage his life, be himself and lead his own life. One that takes valuable lessons from others and is hardworking. One who is patient.

I: How would you describe fatherhood? Within the society you live in, you have told me how masculinity is described. Now tell me how you would describe fatherhood.

R: Speaking from the countryside perspective, a father is one who expects financial support from his children. Various people who have sent their children to the city or if the child is a merchant, then they expect financial support from their children. A father expects to get everything from his children. I do not support that thought. Even if he has sent them to school, he should not be expectant of anyone’s support, one should always be self sufficient by working hard. In my society, many people strive and work hard so you see two types of people, some who expect support from their children and others who do not expect any support. Some believe, since they worked hard to raise their children, that they should expect support, that they should take advantage of their children’s energy and time and rest themselves. On the other hand, there are others who do not expect any support from their children. I support the ones that strive themselves and work hard. This is fatherhood in my society.

I: Can you give me an overview of your roles in your children’s feeding practice?

R: Like I was telling you earlier, I do not have the capacity to provide and meet all their needs. I deeply want to be able to provide everything for them. Children’s needs are limitless. They want new clothes for the holidays, they want to have fun during the weekends, I can not do these. There is a lot that is expected from you. I even work on Sunday afternoons, after taking them to church in the morning, and they ask me why I go out on the weekend. The eldest one understands and lets me go but the youngest daughter refuses to let me go out. I am not the kind of person that provides for all my children’s needs, there are some gaps, but I strive to fill them. Fatherhood, like I said before, requires a lot. A father does not want comfort for himself, he wants it for his children. If I am not able to meet their needs, then I would think

that I have not done anything to raise them better than how I was raised myself. I was raised under difficult circumstances, and they will face the same. But there is a lot that keeps me from doing this, for example finance and money. While your children want other things, you are busy meeting the basic needs.

I: What is your role in their feeding practice?

R: I have various roles. You will be happy when you can provide everything for them. But mainly, they should use whatever is available, efficiently. For example, when they come home from school, I ask them if they have eaten their lunch and if one of them tells me she has not fully eaten her lunch, and when I ask why, they say that the lunch that was packed for them was too much. So, I tell my wife to pack them the adequate amount and she says that she fears it might not be enough and it is why she packs a lot. If they are forced to eat, they will not eat so it must be the amount that they want. The mother's concern is that it might not be enough, but I say it should be the amount that they can finish eating. In their feeding practice, I do whatever I can to be involved.

I: Are you involved in the provision? Buying and bringing home what they eat? Or how is it?

R: I told you earlier that I buy the things that they need. If you are told that what they need is not found around where you live, you go elsewhere to find it. There are things that you always bring home. If I have money, it might be bread or bananas, I never go home empty handed. Who will buy it for them if not me? I leave at 12am in the morning so if I do not buy the things for them, then no one will. In case there was a power outage that day and I could not buy bread, I must leave money for the morning before I go out. They do not have any other source. If I do not buy them milk, then who will? It is me they expect things from.

I: In each photo you have mentioned your roles as a father. Please list out your roles and responsibilities, as a father, in your children's feeding and caregiving. You have told me about your overall contributions in their feeding and raising but what responsibilities can you list, responsibilities you have as a father.

R: Washing their clothes is my responsibility. Buying them clothes is also my responsibility. It is my responsibility. Within my financial capacity, I provide for them. As I mentioned earlier, if I do not buy them the food they need, no one will. So, this is also my responsibility. It is my obligation as a father. Buying clothes. Making sure they are healthy. Making sure they get medical attention when they are sick. If I see that their hair is unwashed and dirty, then I wash them. I know it is my responsibility. I never ask why their hair is not washed and scream. I think about going and washing them because it is my responsibility. I always think, if I do not show them how it is washed, then how will they know? The same applies when I see their dirty clothes. If I do not ask them to get their dirty clothes and wash it, I will not pick a fight with their mother regarding this. It is also my obligation, and I must carry it out before I can fight with someone else about it. These are my responsibilities. In addition, I must teach my kids the teachings and words of God. To ensure they are performing well in school. I ask if they have studied well. The generation that is ruining the country is one who was loosely controlled previously. It is my responsibility to nurture good behavior.

I: We are now finalizing our interview. We are only left with one question. As you know, we are amid the Covid-19 pandemic, if there have been any changes, be it economical or social, in your life, how would you describe those changes? What changes do you believe have come about after Covid-19?

R: Right when it started, for about one month, we all stayed home. We never ate breakfast. We used to pray until lunch time, we are protestants in religion, and eat whatever we have. It is after that, when the situation became more stable, that we started to go out. Since then, when I come home, the children always remind me to wash my hands before I get into the house. I am scared to bring home anything to my kids. I take off my clothes outside and wash my hands before I enter. Sometimes, the thought of bringing home the virus while I go seeking to bring them food, scares me. I pray to God for his protection. Sometimes it is difficult just being scared. You need to be careful. Fear only has no value. Like I told you, my wife is hypertensive and the fact that she has a comorbidity scares me even more. She always warns me. I wash my hand outside using the water and soap that I have prepared and take off my clothes before I enter the house. The worry is not minimized even after doing this. There are a lot of changes. Previously people used to gather for the holidays or drink coffee together but that is no longer the case. Everyone is dealing with their own problems within their own houses. Only sometimes, some people come over and ask us how we are doing, the children. Some people also occasionally drop by to bring us some supplies. This is when I think that I should also visit others too, since they visit us. There are a lot of social challenges, but we are still here, thanks to God.

I: You have told me about the social challenges, that people used to visit each other, drink coffee, celebrate the holiday together. Now, how would you describe the economical challenges?

R: Around the time that the pandemic started, we did not make any preparations because it was sudden. So, there were severe challenges. We could not comprehend how we would be able to pass this difficult time. When I saw how much supplies people were buying, I had a difficult time contemplating how we would be able to survive. I waited in line to buy things worth 300 Birr while someone in front of me was buying things worth over 10,000 Birr. When I got my turn, I wanted to buy pasta and rice, with the money that I have. There is a wide gap between the lifestyles of different people. It had severe challenges. Especially for someone with a family. There are even people who moved back to the countryside because life seemed very difficult at that point. God has gotten us here. It also has social challenges. Despite how little of everything we had and due to God's protection, we are still here. We are grateful. It is not about just wishing you had the things you do not have but you should be grateful because God has protected you and you have reached to see this day. That is how this time passed. The economical challenges were severe. One used to move around freely to work but could not do so at that time. If one wanted to buy clothes, he could not. He had to wear what he already had. This is how I would describe it.

I: Thank you. We have discussed a lot of things and we had a good interview. Maybe, if there is anything that you failed to mention and would like to add on or elaborate on, please use this chance to do so. If there is anything, thank you very much.

R: One can only speak from his/her experiences, and I do not believe that I have had a lot of experience to talk from. So, what I am saying may or may not be useful to you but it might be a lesson to some or just

similar experience to another. For those who have not passed through a similar experience as I, this might be a useful input. There are several things in life, so the main thing is going forward and not sliding back. Always being hardworking and being a strong believer, in the religion you follow, and moving forward. Sometimes, people expect things from other people, but it is better to expect things from God. Through the adversities I have passed in, I believe that God was with me and helping me in every step of the way. So, sharing this experience is not just for your benefit, it is so that others can learn from them. Several people may have passed through worse adversities than I, but it all passes. We shall cross this day into the brightness of tomorrow. This is all.

I: Thank you again for your time.

Father 4

I: Thank you for agreeing to take part in this study. We will now begin our discussion on the selected photos. We will freely discuss about the photos, and we can also discuss on things you want to talk about that I might not raise. Since we do not have time for all the photos, we will only discuss on the selected ones. Let us start with this picture. Please tell me some things about this photo. What catches your attention in this photo?

R: It is difficult for me to talk about this photo currently. The one thing I can tell you is that it shows how much affection and care mothers show to their children. We, be it in the countryside or in the city, during our young age, if we have children, we should help them, not just have children, and leave behind the responsibilities that come with it. Children should be raised adequately. In addition, once you have a child, carrying for him is a must. I have no words, but I want to raise my child better than others and even better than myself. I believe that. It is why I decided to have a child.

I: How old is your child?

R: 6 years old.

I: She is your only child, right?

R: Yes, but my wife is pregnant with our second child. My nephew is also there.

I: Looking at this picture, how would you relate it to your lifestyle or the lifestyle of other fathers in your society?

R: It is difficult. It gives me joy, taking care of my child more than anyone else. More than anything. Why? Not only my own child but I can not turn a blind eye to any suffering child. Within my capacity that is. Whether I have anything or not, within my capacity. I am telling you seriously. Where we live especially, in our area, things are very difficult.

I: How?

R: When it comes to raising children, it is very challenging. What I want to share with you given this opportunity is, in our Woreda[1], there are people who are worse off. I do not know what the matter with their family is, but some are just ignorant. There is no one who hates his/her child but so long as you have a child. I never want to be parted with my child, not even for one day. I do not want to see her dirty, not even for one day. I am also a role model for others. Anyone could attest to this. I never bargain when it comes to my child. Why? I have already brought her to this world, so she does not deserve to be raised the way I was raised. If all I had when I was being raised was Kita[2], then I have to provide something

---

[1] Woreda: Districts in Ethiopia are commonly known as woreda and are the third level of the administrative division of Ethiopia - after zones and the regional states.
[2] Kita: Kita is a relatively thin unleavened bread typical of Ethiopian cuisine.

different and better for her. In addition, if I had Berebaso[3] and Shera[4] only, I have to provide something better for her. The only thing I cannot change, or it maybe something I can change, God willing, is my livelihood and my current living situation. The biggest challenge in our surrounding is housing. If you have stable house, then you can do anything for a living. If you have stable house, then you can do anything for a living. The main challenge is housing. I do not know what I can say specifically. I really really love my daughter. More than you believe. I am still living in this country because of her. I wanted to go abroad for work. It is because of her that I am still here.

I: What is your livelihood?

R: I am a daily laborer. I do any job that I get.

I: Let us return to this photo, because we will not go back to it later, specifically regarding this photo, as we can see it looks like you are scooping some food with a spoon onto her plate, and she is sitting and ready to eat. Is this something you practice frequently? How is her feeding practice and what is your role/involvement in her feeding?

R: When it comes to her feeding, to your amazement, I am always ready for her in the morning. Why, because she goes to school.

I: What do you mean when you say you are ready? Is it by preparing the food or feeding her?

R: Both, preparing and feeding. It is always I who feeds her, dresses her, and bathes her. It is because you did not see the video, but it shows me giving her a bath.

I: Okay. Are you the one who prepares breakfast?

R: Yes, it is I.

I: Always or mostly?

R: Always. Always.

I: How come it is always you preparing?

R: Her mother (pause) has got a small job and she has started doing that.

I: So, it is because she will be at her job?

R: She goes out early in the morning since it is a cleaning job. So, she can not manage. Because of that, this is my responsibility. Therefore, I believe that I should shoulder all the responsibilities for my daughter in addition to my responsibilities as a father.

I: So, you prepare food, feed her, and bath her?

---

[3] Berebaso: Type of shoe made of recycled car tire
[4] Shera: Name of one type of shoe

R: Yes, I bath her.

I: What other caregiving roles do you have?

R: Since the duration of this activity was short, you can not see it here. But we are in a pandemic and jobs are also rare so I can not take her out for recreation. Otherwise, I take her to a lot of different places for recreation. Not only her, but I also take the neighbors' kids sometimes. It is not because I am better off than them, they are much better off economically even. But when I take my daughter out, the other kids look at me pleadingly so I can not leave them. You understand my point. This requires a large budget unfortunately.

I: As we can see in the picture, food is served, there is also tea and drinks. I am repeating this to ensure that I have not forgotten anything.

R: For instance, here, I made her eggs early in the morning. I have also served bread and tea. She is sitting ready after washing her face. After she has eaten, I am also responsible for doing her hair.

I: You also do her hair?

R: Well, I can not do her hair in all the styles, but I can do about half of them.

I: You do what you can?

R: Yes, I do it in the ways I can.

I: Shall we proceed to the next photo?

R: Okay.

I: Until I take out the other photos, you can add on anything that you think might have been missed on the previous photo.

R: Okay.

I: This is the one we have chosen next. What do you see in this picture? If you were asked to verbally describe this photo, how would you describe it and what kind of feelings do you associate with it?

R: She always waits for me at this hour.

I: What time is it?

R: We go to church.

I: In the afternoon you mean?

R: In the morning.

I: Oh, in the morning? Okay.

R: Early, I usually wonder around looking for work. (Long pause) Despite how badly I feel after not finding a job, I will be happy when I see her. (Long pause) Let us skip this.

I: Is it making you emotional?

R: Yes.

I: Okay. We can take some time and proceed after you calm down if you want.

R: No.

I: Maybe you can tell me how you relate this to the lifestyles of other people, looking at this picture. We can go to the next photo. This shows your lifestyle. How do you think this picture shows the lifestyles of other members of the society? For example, do you think other fathers are like you or do you believe they are different?

R: There are. Fathers are not all bad. There are some. There might not be any that cook and serve but there ae some. No father hates his children. In your life, it is better when you care. Everyone's life is different. For instance, I come here early, her mother leaves early for her job and if I find a job that day, I leave for work as well. But when I come back home (Long pause) I am extremely happy. So, if I have something or nothing that day, I will never be sad. I am happy. (Long pause)

I: Shall we go to the next one?

R: Yes.

I: The third photo we have chosen is this. I am going to ask you similar questions with all the photos. How would you describe this photo? Anything that stands out to you or for anyone who has not seen this picture, please tell us, in your own words, why you wanted to take this picture?

R: By the way, her mother does not like taking pictures. Here she is just coming in from work. She is now left with one month before her annual leave. Always, when there is no school, this is where we wait for her.

I: Where?

R: Inside the church. She gets off the taxi at Tekle Haimanot Church[5]. She comes from Tikur Abessa[6]. She gets off at Tekle Haimanot and we go in. My daughter and I get Holy Water, drink, and wait for her there. It is also fasting season and we always sit here to listen to the church sermon. During the fasting season, we are in the fasting season now, Wudade[7], we always want to come to this place. It is comfortable since it is directly in the front for the sermon. It reminds me of last year and the year before last.

[5] Tekle Haimanot Church: An Orthodox church in Addis Ababa.
[6] Tikur Anbessa: Amharic name for Black Lion Hospital.
[7] Wudade: A 2 month fasting period in the Orthodox religion.

I: It is a gathering place for your family? You are in the habit of doing this.

R: Yes, we have this habit.

I: When you look at the photo of the three of you, in the place you usually go to, what do you feel?

R: Well, as a human being, there is a lot that we want. So, I pray. I want something better. As you can see here, her mother is pregnant. To your amazement, I did not want this pregnancy.

I: Why?

R: It is due to the persistence of _______(daughter) that she is pregnant. She said others have brothers, but she does not. One should not just have children and not raise them adequately; one must look at his living situation. You understand. The notion 'just have children and they will grow anyways' does not work. The family's effort is necessary. Just like you need to cultivate plants for them to grow, you have seen her water the plants, right? When I say to her, you always take care of these plants, she says dad, you raised me by always feeding me right. I will never forget this. So, she needs a companion, she should not be lonely. But it is difficult, the timing. I had other plans, I even thought about going abroad for work. When I come back was my plan. I was waiting for her to finish kindergarten. So, this happened.

I: How about her, your wife, does she also feel the same way about having another child?

R: Yes. Her and I, we were married at City Hall, with our family's blessing. We have a deep connection and understanding to the point where we can tell what the other is feeling simply looking into each other's eyes. She warns me with her eyes, and I do the same. Why, because I have two experiences when I was shocked because of my daughter. One time, when I spoke to my wife a bit loudly, my daughter was shocked. Since then, I never want to do such a thing.

I: Is it your daughter that was shocked?

R: Yes. This will influence her because she thought we were going to have a fight. I diffused the situation immediately by telling her this is the same as when we play. So, we solve everything this way.

I: So, your thoughts are the same regarding the pregnancy and adding a new member of the family?

R: Yes, it is the same.

I: You are now used to the second question. In relation to the society, how do you describe this photo?

R: Once in a while, it is difficult. It is not something you can say simply. My wife, she started work when my daughter was just 9 months old. She (wife) used to work on some business. I raised her since she was 9 months old. I changed diapers, cooked for her, bathed her and everything right up until now. It is when my daughter turned four or five years old that her mother left her job and started staying home. I even took her to work when she was one or two years old. What made me emotional earlier is that my daughter has paid a price, along with me. When I go for work, she used to go with me. If I were to buy some things for work, she would sit in the car and wait for me. When I came back after finishing what I had to do, I

would buy her anything she wants like juice. She would sit in the car until I finished bringing all the things. She used to go to work with me. She has suffered this much. (Sobbing) It is better if someone from your surrounding attests to this, it might not mean as much when you talk about it yourself.

I: So, you believe that a majority of people are not like this?

R: There are a lot of people who have a lot of fatherly love to give. There are others. I am not saying that there is not anyone. However, in my age range, people tend to think about enjoying and taking care of themselves. Everyone of us does this. But I think more about my daughter than my own personal enjoyment. This is because if I do not make it home by 7pm at night, she will not eat dinner. She wants me to feed her and put her to bed. So even when I am with my friends, (Sobbing) I tell them I have to leave when I get a call. I will not waste one more minute there after the call. That day, if I stay out until 7 or 8pm, I am disrespecting my family and therefore losing their love. That enjoyment, you can find it anytime, but you can never have enough of the family love.

I: Okay. You are describing it very well. Earlier during our discussion, if you remember, you were telling me that life is difficult in your surrounding and that there are people who are in a worse situation. Relating to that, I want to make the question clear. This study is being conducted in your sub-city, Lideta sub-city. Paying a price to raise a child, as a father, when saying paying a price, it might be giving love. I want to understand what you mean by paying a price/sacrificing. Please elaborate more. In what ways do fathers make sacrifices for their children and for you, when you say you made sacrifices, what does it mean?

R: When I say sacrifice, I do not use it lightly. You will likely make a sacrifice, not only to someone who is related to you, but to a stranger also. I, God forbid, would donate both of my kidneys to my child if I have to. Why, because she is still young, and so she will hold me in her heart forever. Forever. I give you my word, my word. I would do anything, anything for_________ (daughter).

I: How about other fathers in your society, what type of sacrifices do you think they make for their children, from your expectation or from what you have seen?

R: You can not simply describe sacrifice. Sacrifice, sacrifice, for instance, you make a sacrifice when you are able to raise your child from his earliest days. That is when you feel excited and loving. Otherwise, if you leave in the morning and come at night, then you will not have any love for each other. It is when you have love that you are able to make a sacrifice. Those who have a taste of what child's love is know this. When I go somewhere, my mind always wonders if she has eaten her lunch, if she is safe since there are a lot of cars that pass by there. I just remembered something, I went to buy her cake and I was hit by a car, with her in my arms. The car stepped on my leg, and my leg bended. Through my shock, I pulled my leg out from under the car. What was going on in my head was my daughter's safety. This was around Mierab Hotel. The driver was a woman. Through the shock, I jumped from under the car. Through a coincidence, a neighbor was there, and he knew who I was. My mind was on my daughter. I was holding her, but I could not believe she was okay. Even while I was holding her in my arms. It was like a sprain; my leg was bent.

I: Is this when she was a baby?

R: She was three years old at the time. Two and a half or three years old. She (driver) took me to get the necessary medical attention. She only had 100 Birr in her pocket. I could not say anything. What I said to her was to thank God because had my daughter fallen down and gotten even a scratch, I would have killed her. This is the level of sacrifice I am willing to make. Not only your child but you will be scared and shocked if something even happened to a pet dog you raised. It is very difficult. How do you feel when something you wanted, loved, and kept safely goes missing? This is worse.

I: We are having a great discussion. To use our time effectively, let us go to the next photo and discuss on that. This is the photo we have selected next. Let us now collect our attention to this photo and tell me what things stand out to you in this photo? What is your reflection.

R: In this picture (Long pause), I usually tell her how happy I would be if she were to become an athlete. She asks what an athlete is. She says, is it not better if I become a doctor and help you? I tell her being an athlete is good and she says that I am saying that because they earn good money. I then tell her that I am joking and that she can become anything that she wants. I just tell her to be strong to become what you want to become. In the mornings, she likes to wrestle with me, even when we are eating, drinking, or playing. Like when you argue, you argue with the person you want to argue with right? She has such type of behavior. Even for a second, if I am not there, she will be miserable. I can tell you one thing, if something happens to me, she will never recover, never. In the morning we do a training exercise, over by the street. When you walk out, there is a street, you saw it earlier, where the dogs were standing. We did not want to disturb others since some people were washing their carpet, so we played on the street. We are playing in our surrounding area.

I: So, this shows you doing sports?

R: Yes, we do this on Sundays.

I: Sunday?

R: Yes Sunday. This is not just for this. We used to exercise even when she was three years old. She is always skinny thought. No matter what she eats, she is still skinny. (Laugh)

I: You are exercising. Okay. You have told me that your wife is busy with work, and she leaves the house early in the morning, but does she participate in activities like this, when she has time?

R: My wife waits for us at the church while we do our exercise and go to her afterwards. She waits for us at the church. We then meet her there, drink Holy Water, and come home together.

I: On Sundays?

R: Yes, on Sundays. After we come home, we go to the shower.

I: Now, like you told me, she (wife) is pregnant. I meant during the other times, like going to church, is there anything the three of you do together? Something you frequently do.

R: Yes, we go out together. For recreation.

I: How about at home?

R: At home, the three of us are inseparable. Granted, the house is not that comfortable because it is small. It is a small house that I built next to my family's house. We even communicate non-verbally. My wife and I, we can understand each other just by looking at each other. She gets me what I want, and I get her what she wants.

I: How do you think she (wife) feels about you as a father and your caregiving roles for your daughter? If she has expressed her feelings to you or like you said, since you know each other very well, if she were to be asked about your fatherly role, what do you think she would say?

R: I do not know. It is difficult. She would probably say it is difficult without me. It would be better if you hear it from her.

I: From her perspective, how do you see yourself? As a father?

R: Edilawit, I told you she would be miserable. I have told you this from the start. Edilawit is a part of my brain. There is nothing I call mine; everything is hers. As we are sitting her, she is calling me. She is probably asking where I went. They go to school in shifts. The time for lunch, dinner is known right? Aside from that, she looks for me, just like a child. Who looks for me? My daughter. She acts like a parent and looks for me. She understands my work situation, how I work and what time. Outside that, even during the day, she looks for me. She has no one besides me. I am the one that treats her and takes care of her. It is God's blessing and so, when I have my second child, I am going to treat them equally, but the first child is always challenging. It is challenging. I have been through a lot because of her.

I: We have two photos left so let us go on to the next one. This is the fifth photo that we have chosen. Briefly tell us what you see in this photo.

R: It is clear in this photo that I am very exhausted. I came in from work. I was exhausted. She was eagerly waiting for me. You can see that she is hugging me tightly and kissing me. (Pause) After putting all the things in place, (Long pause), there was something there, you skipped it, she took the broom from me and was sweeping for me.

I: She was helping you?

R: Yes, she was helping me. Because I was very exhausted, and since her mother was also occupied with making lunch, she (daughter) was sweeping instead of me to help me.

I: Okay. Shall we go on or is there anything you want to add?

R: No. This is it.

I: We are on the last photo now. This is the last photo.

R: There was one photo that you took of me and my wife, right?

I: This is the photo you selected last but if there is anything you want to say about the other photo, we can go to that when we finish.

R: Okay.

I: Please describe this photo in words. What do you see?

R: I call her my model. She is interested in that. When I call her a model, she acts out more. She has a lot of pictures on my phone. She takes pictures like a model. The house feels too small for her when she acts out. (Laugh) I regret not keeping a copy of this photo for myself.

I: You can take a copy.

R: I can, right? I would be happy if you can send me all of them.

I: We will.

R: You will save it on my memory card. She is perfect.

I: You have said that she is your model, so you see beauty?

R: Yes, she is a model. More than anyone. You can see us arguing. Jealousy. If my wife does not marry a second husband, the jealousy my daughter feels is insane. We have a time for playing house. We also have a specific time for studying. I tell her to play earlier and to go to her studies in the afternoon. Because even though I am not able to give her a big inheritance, it is my duty to give her love and education. I have nothing. Nothing. I have to provide her an education, feed her, and give her love, that is my obligation. Even if I have riches, I will not just simply hand her an inheritance. Even if I have it, I mean. I want her to work herself and reach to a level of success by herself. Naturally, I faced a lot of challenges to get to where I am now. I did it all by myself, without anyone's help. It is amazing. I have a mother, she raised me until I was nine or ten years old. Since then, I strived to get to where I am. If I were to go off the rails, or become a vagabond, but thanks to God, I am here. I have also created two people. So, I want to make her someone too.

I: Earlier you said that you encourage her to become anything that she wants, and you also said that you tell her to become an athlete but now you are telling me that you call her your model. But deep inside, is there something specific that you want her to become?

R: I, (Pause) would like it if she became a doctor. There are a lot of people in hospitals, I have stayed in hospitals to help people, voluntarily. There was a time when I spent my time in hospitals helping people instead of wasting my time elsewhere. Doing a good deed is always good. There is someone I know, he is younger than me, he suffered a major accident. What you see in the hospitals and what other people complain about is very different. Outside, you complain about not having money, but in hospitals, you will be grateful to God. Once I get a comfortable shelter, I can do anything, with God given health. When you see hospitals, you even lose your appetite to eat. You see people losing body parts, some people have difficulty breathing, I have witnessed all this. There is a lot out there. We are human, we always desire

more. She is the reason I came here. My friend told me, and I told her that I also have a daughter. I put my trust in her (friend) and came here. It is good to be trustworthy, I had no idea when I came here. But in the future, will this be beneficial to my daughter or not was my question. After your explanation, this is a research, not for media. There is one thing, I am aging, and I do not know what tomorrow holds, for me or for her (daughter) but seeing your child reach a goal makes you very happy. It is for herself, not for me. For herself. That is it. There is also one picture that shows her studying.

I: Tell me.

R: I took this photo on the sofa. (Long pause)

I: Tell me about it until we find it.

R: This is what raising a child is like. It is stressful. You do not know what tomorrow might bring. In addition, it is more difficult raising a daughter than a son in our area. Girls are challenging.

I: How?

R: If parents are not strict when raising a daughter, it will not be okay. Not only for girls but the area is difficult for boys too. You need to make them pay attention to school, otherwise. Once in a while though, there are some who are smart. Some have even gotten into NASA, but it needs immense effort from the family and parents.

I: What makes it difficult especially for girls? If anybody, who does not know where you live, were to listen to your interview, for someone who does not know the Teklehaimanot[8] area, tell us clearly what makes it specifically difficult for girls?

R: For instance, you live in poverty, in that area. Because I am poor, I can live without any worry. But you can not give your children the same freedom.

I: How?

R: Think of the area. It is the Merkato[9] area, it is Teklehaimanot area. Every vagabond that comes from any area comes there. Because there is business there. Daily business. Not consistent and reliable business but daily business. If you give ample freedom to your daughter in an area like that, she will rebel.

I: How?

R: I am glad you asked. At an early age, I will personally pick and drop my daughter to and from school and when she is old enough to know for herself, I will give her advise and give her the freedom that she needs. I will tell her to look after herself and not to be involved in any malicious activities. I will not say do not go out with any boys or any such thing. I will tell her to protect and look after herself, myself or along

---

[8] Teklehaimanot: An Orthodox church marked as a transitional area between the active market center Merkato and the rest of the city.

[9] Merkato: The central marketplace in Addis Ababa. It is also known as the biggest open market in Africa.

with her mother. By the way, I take her to the cinema. Sometimes she closes her eyes on some scenes and I tell her not to look, but that she has to know. She has to see what is good and what is bad. Because she has to understand. It is when you hide things that they can not understand you. If she knows and understands, she can judge for herself. She could say 'how could he do this to her, is he dumb!' Do you understand? But if you just tell her without showing her, she will be damaged. Without knowing anything. This is why I like doing things in the open. I do not pressure her a lot, but I do try to control her a bit. Other than that, I do not take away her freedom. I, when she reaches a certain age, do not mind if she brings home a boyfriend. I will just ask if he will be a good fit for her. Is he someone you want until the end is what I will ask. If he just wants to use her and leave, then that is a different case. That will spark a fight between her and I and with him and I too. So, when she reaches that age for marriage, it will be an honor for me.

I: We are losing track of time. There are one or two questions left. Not just specific to one photo but regarding all the photos, to hear your overall opinion. In the area that you live in, how is manliness or manhood described? What qualities should someone have in order to be considered a man?

R: In our area, when you say man, if he is not educated, then he has to go abroad for work. He goes abroad to get money. I have told you this.

I: Let me make sure that I understand you clearly. Are you saying that if a man has no money or education, then he is only considered a man when he goes abroad for work? Or is this something he has to do? Please elaborate.

R: For example, if I am not educated, not trained in some profession, or have no money, what I need for survival is money right? So, what do I need to do? Here, in our country, you will not earn enough money, despite how much work you do. But elsewhere, in another country, even if you are a daily laborer, you will be paid adequately. So, here I was working extremely hard and exhausting myself, like you saw in the photo, I was extremely exhausted, and I was angry because my effort and the amount I was paid were not compatible. I was very angry. Then when I saw her, I felt better. When she hugged me and kissed me, I forgot all about it. It is because of her that I forgot about it. I would have gone abroad to work had it not been because of her. Even if you go to the Arab countries, not only Europe, you will earn enough money and come back. It is not like this here. I do not know. We humans, go around covered and disguised in different clothing, no one knows what anyone actually thinks inside. Especially in our area. Majority of the people are unemployed. Majority of them. Majority are unemployed.

I: So, manhood is when someone tries, to the best of his ability, to go work hard and earn money? Is femininity different from this? If it were a female?

R: Women are also not spared from going abroad for work, in our area. She will go abroad. In our area, many go abroad. It is all to find something better. When you see someone going abroad and coming back successful, you will also want that for yourself and your family. You will think of the life you can provide for them. If you work one or two years abroad and even manage to buy a Damas[10], then you can make

[10] Damas: Type of vehicle with storage space in the back, usually used for business.

money through that. The question is, how will you find that money. You understand my point? You would be disgusted if you knew the type of jobs that I do. You might have a different opinion about me just by looking at me. But one will do what he/she has to do if they have to. I believe in that, starting from the bottom. I have done many nothing jobs. Everything. I have worked on brick manufacturing. There is nothing I have not done. I did everything. But I still have nothing. You will be riddled with disease when you do too much of labour work.

I: You have described manhood well. Now how would you define fatherhood? Are the two similar or different? How?

R: Fatherhood, from what perspective?

I: How would you describe being a father? What are the defining characteristics of a father?

R: Characteristics of a father? Characteristics of a father. There are various characteristics that define a father.

I: For example, a man who is a father and another man who is not, are different right?

R: Yes.

I: So, tell me based on that.

R: One characteristic of a father that I possess is if I see other children on the street, I will not turn a blind eye and just leave them there. Because I am a father. If I see a hungry child, I will not turn a blind eye as well. I will split what I have with that child and my daughter. Because I do not know what tomorrow might bring and what will happen to my daughter in the future. If you do not help people, then people will not help you. You do not need to do this just to impress people, even if there is no one to see your good deed, you should be God fearing and do it. If you are not kind and you do not help people, then people will not help you, never doubt this. You will not get the reward back from that person, you will get it indirectly. This is what I believe because it has happened to me. (Long pause) I have nothing further to add.

I: Okay. We have discussed on your various caregiving, child raising, and feeding roles, with the photos. When it comes to caregiving and raising a child, what components do you think will be good or are there any other things you wish you could do more on, as a father? You can suggest on what things could others do/external/ to help parents in their child raising practice. Do you have suggestions like if you had any materials, or knowledge that would make parenting easier?

R: Do you mean finding a livelihood to provide for my daughter and my unborn child?

I: Yes, it can be a form of livelihood. But my question is, when one raises children, what are essential components that he/she need to have?

R: The major thing is housing. I can do without anything else; housing is essential. If you have your own house, then you can live freely. The housing situation is very challenging. Very challenging. The question my daughter always asks me is, how long we will be living in such a small and narrow house. Imagine it.

We live in the service quarters. It is 2x2 meters. It is very small. I can show you here on this picture. This is it. It is fully covered in carpet, and we have a TV stand here, nothing else. We have a small balcony where we keep our cooking utensils, that is it. How do you live in a situation like this? Would you wish to bring another child? But we are human. There is also the biological clock for women so I have to be understanding of my wife's needs because she may not be able to have more children in the future. You might also be able to have everything you want but not have any children. It is not good to push everything for tomorrow. I believe, what if you can not find that thing you pushed for tomorrow when you could have had it now? Everything happens for a reason. What if I do not have a chance to get it in the future? How am I going to raise them? I want to raise them properly. I do not want my children to struggle. I do not want my child to grow up the way I grew up. You understand. I have never received my father's love; I never knew my father. I only saw him once when I was about five years old. We went to Asmara with my mother in the military camps, that is when I saw him. I do not know what a father's love is like. But I love my daughter. It is not something I received from my father, but I do give it to her (daughter). She needs to receive what I lacked growing up. (Long pause) Overall, it is very difficult.

I: Please tell me your responsibilities, as a father, in your child's caregiving and feeding practice. List out your roles and responsibilities.

R: I do everything for her. It is not even something you can count. When you raise a daughter, or a son, your responsibility starts from their underwear, their nails. Your fatherly roles. It starts from their nails. Even in schools, if the nails of the students are long and dirty, they ask if they have parents or not, right? So, it starts with their nails. I have various responsibilities, various. I always think of how I will shoulder these responsibilities and undertake them effectively. I am human so I always worry. I think about the future a lot. You will be criticized if you do not do it adequately. I hate that. I especially hate being criticized as a father. It is shameful. I would rather die. You can be clean. Even in poverty, you can lead a clean life. You can, right? It is simple.

I: You are right. We have finished our main discussion. Lastly, we a re amid a pandemic, it is something new and different than anything we have seen before, the Covid-19 virus is spreading fast. Since the pandemic, are there any changes that have been brought about in your family, society, and overall life? Any stress brought on or good changes that came due to the Covid-19 pandemic? It might be work related or other things.

R: This is a sensitive topic. I can not even describe it. The pandemic has brought on various things. To your amazement, Covid has derailed everything for me. Due to it, I have even used up all my savings. In addition, you can not go around and work freely.

I: Why?

R: Because you need to go find things to buy and look at them well before buying if you are to sell them and make a profit. In addition, there is also an ongoing conflict. Everyone is struggling with their lives, and Covid, and with each other. It is a trying time. I have my own prediction, a priest has also said this, that it will be worse until the coming year of 2014. I believe in that prediction. There is a lot of blood to be shed. When you think about this, the thought of having children is disturbing. I say this seriously. Having seen

the situation in Rwanda, it is disturbing to have children in this current state. It is better not to have them. I mean it. Seriously. I follow the media. It is sad what is happening to adults but what is happening to the children is even more depressing. It is excruciating. Disease and hunger, can you imagine it? In such a cursed time with this disease, people are also slaughtering each other on the streets. Think about it. You might be Tigre, Amhara, Oromo, or anything but at the end of the day, you are an Ethiopian. If you and I were to go abroad, we would help each other because we are Ethiopians, not because I am of one ethnic group. Not because of that. This is my usual prayer when I go to church.

I: In addition to Covid, the situation we are in now, the conflict, has had an impact on you?

R: Yes, more than I can say. More than anything. Because I can not go out to work, I stay home and look after my daughter. Imagine it. My wife's salary is negligible. It is less than two thousand Birr. So, I need to work. This is what makes you bitter and pushes you to consider going abroad for work. You understand. If things were comfortable here and you could do your job safely, you will live happily and gratefully. I mean it. I do not know, it is hard. It is especially hard when you have children. Not only for your children, but you also have no way to guarantee your safety to come back home. I always confront the schools when I go there. No one should be allowed to go into the school. I have raised this earlier. There is only one strong teacher there controlling this. She has made sure that no one enters. Everyone waits outside holding their ID and they pick their children from the gate.

I: Because of Corona?

R: Yes. It is said that children do not catch the virus, but I do not believe that. God protect us all.

I: We have raised good points and discussed on them very well. Thank you for the time we have had. I have completed my questions. If there is anything that you think is missing, something that was not mentioned during our discussion, I would like to give you a chance to raise them. If not, I have finished my questions.

R: Given the chance, what I would like to say is, is this interview going to be in the media?

I: This is not for the media or for a promotion, this is a research and a study, like I told you earlier.

R: Yes, you told me. I have understood.

I: The aim is to improve the overall feeding and caregiving practice; your daughter might not be a direct beneficiary, but this will be an input to identify the working gaps and how best to intervene to improve them. Children's nutrition, feeding, caregiving practices and the father's roles and involvement and how best to support them is the concern of the study. This is purely a research work; it is not for the media.

R: For people living in a lower socio-economic status, will you provide different education and training opportunities?

I: You are not asking me directly about this study but overall, about research work?

R: Yes.

I: The research study is continuous. The next phase will be working on the intervention, which is on support. After that, there is work on input. This is conducted continuously and in phases. Like I have explained, your participation with me, is voluntary and is aimed to capture how your views of the world are, from your perspective as a father. This is the aim of the study. This is your work with me. Our selection criteria are any father living in the selected area with a child in the targeted age range. There is no preference of economic status, educational level, religion, or ethnicity. In addition, the selection criterion is your willingness and voluntarism to participate. These are the three things. After that, we conduct our study on a few selected fathers and gather their input. Once that is done, this will be used as an input to the concerned stakeholders. It might be for policy change or anything else like an intervention area for the projects of NGO's. This is the aim of this study.

R: I hope God provides us with a solution for our current situation, including the pandemic. It is a very trying time. Think about how everything is. Due to corona, the taxi tariff has also increased. To your amazement, everything is expensive in this country. Abroad, things are cheap, even the things sent from here. Here, it is difficult, I do not know. It is not something you can talk about easily. It is difficult. It is even hard to say we are living. I do not care if anything is said in the media a thousand times. The things you hear are disturbing. If I am struggling like this with only two kids, then in the future, I am going to bargain with God not to give me any additional kids. It is true that when you have a stable marriage, you can pass the difficult times. Especially if you are a woman, you might not have the opportunity in the future. I told you this earlier. This will also spark a fight in your marriage. It might even lead to separation and divorce. You might delay having children until you have a stable income or other reasons, but it might lead to your separation thinking that one of you do not want this. That is what will happen. After that, you will struggle with life and probably go back to your family. Because life is getting more and more expensive each day. Even one exercise book is expensive. Thank God that the government provided the exercise books and school materials for students at the beginning of the year, it has helped many families. They are also feeding them in school. We are also heading to that direction this year, towards government schools. God be with us.

I: Okay. Thank you for our time together. Thank you for volunteering and giving us the information.

Father 5

I: Thank you for agreeing to have this interview and for coming here.

R: Thank you.

I: You have brought the photos you have taken, and we have chosen this as the first photo. So, what stands out and grabs your attention in this photo? Are all three of them your children?

R: No, two of them are my children. The girl and the little boy in the middle; the other one is the neighbor's kid. This is a photo that shows them playing in the house. This is how they spend their time.

I: How old is the girl?

R: She is 7 years old. My son is 3 years old. She goes to school, but he does not go to school yet.

I: So here you see them playing. Is there any other information you want to add? Maybe about their interaction with the neighborhood children or any other information?

R: Like all children, they play with the neighboring kids, in order not to lose the social life. That is it.

I: Shall we proceed to the next photo?

R: Okay.

I: How about here, what stands out to you? Is this your second child?

R: What stands out to me here is the joy my son is feeling. I was walking in from work, and I had told their mother to take photos like this, so she took this picture as I was walking in. It was a coincidence, how the photo was taken.

I: Okay. How about here, what stands out to you?

R: Here I am reading spiritual books to them. These are spiritual books written specifically for children. I was reading those for them. There are stories and historical contents. It is specially written for children. I read this to them when I get home from work.

I: You have told me that your daughter is a student so after she gets home from school and you get home from work, do you help her with her studies? Or who is it that helps her?

R: Yes, I help her study and do her homework. Her mother helps me in picking and dropping her off at school. It is I who helps her study.

I: What grade is she in?

R: She is in second grade.

I: I think this is also similar.

R: Yes.

I: How about this? What is it and what stands out to you?

R: This shows him (son) laughing while his sister is dancing. She is not visible in the photo. This shows how close we are as a family.

I: Can you describe fatherhood here? How would you describe it here? If you want, we can go back to the previous photo that shows you reading to them. In this photo, or in any of the other photos, how would you describe fatherhood?

R: Being a father is difficult. You make a lot of sacrifices. Especially in our society, fatherhood is difficult. In our society. You have to make time for your children. That is what I see here. I do not have a social life outside of my family. At this time, they are my friends.

I: You have said that being a father is difficult especially in our society so what are the things that make it difficult within our society?

R: Our living area is an area where people of the lower economic class live. In addition, given that it is a poor socioeconomic setup, it is not good for our health as well. For instance, I did not take a photo from the outside but, as soon as you step out of our house, there is a toilet. There is not even a one-meter gap between our house and the toilet. We live within an area like this, so it is very challenging. Our living area, in terms of health and work, it is very challenging. It is not ideal for living, especially for children.

I: How would this photo describe the lifestyle of the society? How would this photo describe your lifestyle? Not just this but on all the photos. How is it related to your lifestyle as well as the society's?

R: If we are just focusing on visuals, what we see does not describe our lifestyle. Because at a glance, my home might look good, but it is us that knows the actual situation. My children do not play outside, I forbid them from going outside for the reasons that I told you, their health. So, when they stay at home, the house should be as clean as possible for them. That is what I did. Otherwise, we have a very low socio-economic status, and our area is very challenging. Our main focus is on going abroad for work. I have lived for many years in Libya and Sudan, and I still often travel there. Not now because of Covid. We go illegally and that is our source of livelihood and the money we use to provide for our children. Otherwise, I go to Merkato and buy some things to resell them at a profit. That is my livelihood.

I: So, you live in an area where the socioeconomic status is low?

R: Yes, very very low.

I: We are only left with one photo. How about this one, what stands out to you here?

R: The children have a special affection for me. I call it being lucky. They love me very much. Even more than their mother. They spend their days with her (mother), but they have a special love for me. It might be God's gift. I do not know. He (son) comes and sits on me, and other things too. It looks like he is about to kiss me, but he is actually trying to bite me.

I: You have mentioned that the children are closer to you than to their mother, why do you think that happened?

R: It is from the love and affection I give them. With whatever time I have, I told you earlier that they (children) are my friends, I spend most of my time with them, if I do not have to work, I stay home. I play with them just like I am a kid myself. I think that is the reason.

I: Because you give them time?

R: Yes. Because I give them time.

I: Thank you. I will now pass to the next question. In the society or area, what are the defining characteristics of manhood or what is it like being a man? How is manhood described in your surrounding area?

R: It is a bit difficult in our area. In our area, manhood, you have to be chatty. You also have to be serious, that is demanded of you when living in an area like ours. People are advantage takers. You see lots of fighting and altercation. To survive, there are plenty of people who would commit crimes. In our surrounding area. So, you have to be very serious. Especially for outsiders. In your home, you give the deserved respect to your wife and children.

I: You have mentioned that being a man in your society requires you to be serious or aggressive and the ability to defend yourself. How about in your own life, within your family, what is being a man?

R: I, luckily, am a conservative person. I have spent majority of my life abroad, as an illegal migrant. I do not even have friends here, there are some I grew up with, but none of them are close friends of mine. I met my wife when we were both migrants. So, I go and come back then in turn, she (wife) goes and comes back. We take turns in doing this, so we have a sense of equality at home.

I: You have talked about equality. I assume that you believe in the equality of men and women. Please elaborate more on how you would describe being a man, specifically in your life?

R: I define it as being a provider. I am the major provider for my family. I migrate for work, if necessary and if I do not need to migrate, then I work here actively by running around to find different jobs. If she (wife) is in this country, then she stays home, she does not have any job, I do not give her the chance to do so. This is because as a woman, if she is going out for work, that will be added pressure on her. But there are instances when she went to Arab countries for work and stayed there for one to two years. I am the main provider for our family.

I: You have described manhood for me. How about fatherhood, within your society or area, how would you describe fatherhood?

R: Fatherhood, it is different in our area. For instance, more than 90% of fathers in our area, have the name only, none of the responsibilities. You have children, it is the women who go out and work, be it as small venders at Merkato, since our living area is close to Merkato. It is women who provide for their

families and raise their kids. Living together within a marriage is not common. Even if they have children, they live separately in their own homes and the pressure and responsibility to raise the children is shouldered by the woman. Women are the ones who raise children in the majority of cases. But we (with wife) live together, married. I am the provider for the family.

I: Why do you think this is? Is there any influence that the area imposes or what is the reason?

R: It is the lifestyle within the area. The area has a special name, it is called "Tureta Sefer"[1]. I believe it was during Emperor Haile Silassie's Reign, the area was where retirees settled. This went on during the Dergue Regime, the area was used as settlement for retirees and the poor. That is why it is called "Tureta Sefer". Most of us were born there since our grandparents lived there. Majority of the people have no source of income and people's houses are 3m x 3m at most, and they have children on top of this. They may even have grandchildren within that house. This is the factor that influences people in our area.

I: You have described fatherhood from the perspective of the society. How about in your life, within your household, how would you define fatherhood?

R: Being a father is great. It is something you dedicate your entire life to. Life before and after you have children is not the same. Many things change after you have children. It is a source of anxiety and excitement. You believe that your presence is purely for your children. They are my reason for living currently.

I: What are your roles and responsibilities when it comes to your children's nutrition, feeding habit and caregiving?

R: I will not lie to you; their mother mainly plays the caretaker role. I lean towards outside work to earn money and provide since we can not both spend our time at home taking care of the children. I find ways to earn money and provide all the things that they need. She feeds them, takes care of them, bathes them, and dresses them. Their mother.

I: So, you earn money, and she takes care of the children?

R: Yes.

I: Who buys the groceries and other necessities for your home, is it you or her?

R: It is I.

I: It is you?

R: Yes.

I: So, within your financial capacity, what type of foods do you buy for your children and why?

---

[1] Tureta Sefer:

R: As taught by health professionals, for the first 6 months, breastmilk is sufficient for children. After that I provide supplementary foods, to some extent but I can not maintain that because of financial constraints. After that, they eat whatever is available at home. I occasionally bring them fruits when I get home from work.

I: What do you mean by supplementary foods?

R: I mean foods like NIDO[2], Cerefam[3], and some milk products.

I: Doesn't it mean that you are contributing to your children's feeding and nutrition when you provide these things that you mentioned?

R: Yes, that could count as a contribution, but I just meant it is not as much as what their mother does for them.

I: So, what do you think can be done? You said you do not have time, that you need to spend majority of your time working, so what can be done or corrected in order for you to have enough time to take care of them? What do you think you will be able to do in the future to ensure you have sufficient time for them?

R: I, luckily, have been given an opportunity for work, as a returnee from migration, in a small enterprise. Around two and a half years ago, I was provided this work opportunity. We were provided loans. I suffered a loss. Again, about one year ago, after Corona, I was working in the hospitality industry, and it was also shut down due to Corona. So, I suffered a loss again. I had to let go of my employees too. Now I am self-employed. I still have the workspace, but I am still in debt, and I am not in a position to pay it off. I provide for my family, pay rent for my workspace, and I have employees. If I get support on these things, then I can better provide for my children.

I: What do these photos, all together, say about you as a father?

R: The photos show my love, fatherhood, connection with my children. I believe that we should be even closer to our children. They need us. They need us more.

I: You have said that we should be closer to them and that they need us. How should one be closer to his children? How do you think the children need their fathers?

R: Fathers should give time to their kids, not just feed them, children need time. Kids are like blank paper, so this is the time to shape them. Especially in our society, at age five and above, children start to form their own behavior and start to imitate their surrounding, so we have to make sure that they do not imitate their surroundings. If possible, it is better to remove yourselves from that surrounding, after getting things in order. If it is not possible to remove yourself from that surrounding, you have to find a way to team them and lovingly guide them in order to prevent them from forming a behaviour that mimics their surrounding, because our surrounding area is a bit challenging. After the age of 5 years, kids could

[2] NIDO:
[3] Cerefam:

become spoiled. If you came to our area in person, you could witness this. Especially girls. Girls are more victimized in our area.

I: When you say victimized, what could possibly happen to the girls in that surrounding?

R: Unplanned pregnancies. Addiction. You can see these in children as young as 12 years old, in our area. You can see these in boys too, but girls often face unplanned pregnancies.

I: Is that why you do not allow your kids to get out of the house? You have said it is for health reasons. Is there another reason?

R: The first reason is their health. There is a toilet outside our door, not even 1 meter away. Secondly, what they see when they go out is people abusing different substances, and that will be carved in their brains, and they might want to do that in the future. In addition, girls can face gender-based violence. Nowadays, you hear boys facing those too. All these worry me, so I do not allow them to get out of the house.

I: So, they stay home and play?

R: Yes. Other than that, when we have time, we go to recreational places, and they play there.

I: I will move to the next question. You have told me that you had a business and that you suffered a loss due to Corona. We are not amid the Covid pandemic and what kind of changes have been brought about due to this, in your life?

R: Life is more difficult now, more stressful. There were things that we provided for them previously that we can not do anymore so they ask questions. They ask why the things that they are used to are missing. They ask questions like why we do not buy or why we do not do things for them. There is that. In addition, the things we regularly use, including our groceries, have reduced. We can not get anything as we wish.

I: Are there any economical changes?

R: Yes, we have suffered economically. I meant economical change previously. Our earnings have reduced so I do not even spend my days within the small enterprise I work at, and I move around to find other jobs. In addition, I have a driver’s license, so people call me to drive them on field visits outside the city. When I get the opportunity. This is why I could not make it last time.

I: Okay. So, it has brought about economical impact?

R: Yes.

I: How about social impacts, what changes have been brought about?

R: Due to Covid, social life was what we used as a stress relieved in our society. Cooking and eating together. Living together. When those things were discontinued, it felt like trying to clap with one hand. You take something from one house and borrow something from the other. That was how we survived this far. When that stopped, we faced challenges in our social life as well.

I: So, meeting with each other, eating together, drinking coffee together, like previously, is not practiced now?

R: No, it is no longer practiced.

I: I have finished my questions. I now leave the floor to you if you want to add on the points we already raised, if you want to add anything on fatherhood within your living area, or if you want to raise any other ideas. We can discuss on anything you want.

R: Coincidentally, it was my wife who filled out the form when the health extensions came to our home. When you called me, I was happy, what you wanted was also fathers, I came happily. My question is, your facility, be it private or government owned, I did not check, what is your plan for these children regarding their living area, their education, their health? What are your plans?

I: I might not be able to answer your questions fully, but I will tell you what I know. As part of Lideta Sub-city, when we tried to study the roles of fathers, it is not just so it can go to waste. After identifying their roles, we will identify what we or the government can do. We will provide this as an input to the government. We will not provide the photos or your interview recording, we will only submit the final result we find as a report, to the government or relevant stakeholders. We, as an institution, can only ensure that your voices are heard. There might not be anybody that asks each and every one of you but we can put the data together and be able to reflect the fathers' efforts and that there are still problems in some areas. That is how we can help you. If you have another question or if you want to add anything, you can use this opportunity again.

R: I have no more questions. The videos, even if they are shared and viewed, it is not concerning, because it shows the reality. It does not concern us since there are worse situations. This is why I asked you to see it in person. I have told you a few things but seeing it will give you more information. So, it is okay if it is shared and viewed. No problem.

I: Some of the photos are personal and can not be shared without consent. If what you are saying is that you are consenting to us using your photos as a report, then we can use it.

R: Yes, no problem.

I: Okay. Again, thank you for your time. We have finished our interview.

R: Thank you.

Father 6

Interviewer: Ok I would like to say thank you for your willingness to have an interview we us.

Respondent: I want to thank you too!

Interviewer: Based on the selected photos we will start our interview so; we have selected 5 photos so the first photo will be this one so can you please tell us what did you see on this picture?

Respondent: On this picture you can see that I have 3 kids, 2 boys and 1 girl so the first child is a boy and he will be 7 yrs old now and the second one is girl and she is now at the KG 1 and the 3rd child is a boy is now 2yrs and 6 month and we are eating Genfo for dinner. I would like to give my time when I get bac from work and this is all about this picture.

Interviewer: Thank you for your explanation and I just want to ask you one thing for more explanation and previously you have mentioned that you are eating dinner together so is this you every day experience did you eat dinner together with your Childers?

Respondent: Yes, usually we will eat together but will be feeding the last child since he is little and I don't feel that he is eating well.

Interviewer: So, you are saying that on most of the times you will eat together as a family?

Respondent: Yes

Interviewer: Ok

Interviewer: so, we will pass to the next photos so what did you see on this?

Respondent: From this picture you can see that my first son and the next child is washing their hands.

Interviewer: Did they wash their hand always or they do it at the night time?

Respondent: No, they wash their hands before they eat, after getting out from toluate and when they go to sleep. Since they both are going to school, they are getting with many peoples b/c of the current situation (COVID) they will use sanitizer even when they do not get water. Even they will wear a different close when they get back from school if they want to play.

Interviewer: You mentioned that early you are working all day so is your wife taka caring the children's?

Respondent: Yes, she is the one who is taking caring the children's now but previously she used to work but now she has quit her work b/c she wants to give more time for them since the first and the second child is going to school – Frist one Is on the 1st grade and the second child is on KG2 she wants to follow them closely. So, this picture shows that they are keeping their hygiene

Interviewer: Ok we will pass to the 3rd picture what did you see from this?

Respondent: We will see on this picture I am with my last son and it is night time and I am helping him to change his close before he sleeps.

Interviewer: You have mentioned earlier that they have different closes for different purpose like school uniform, close for playing and the like so which close is this one?

Respondent: I am changing his close after playing and eating his dinner so this is his pajama.

Interviewer: We have reached now on the 4th picture so what did you see from this?

Respondent: This is my daughter the second child. She used to play at the salon with her brothers and also watching TV and she will fall to sleep on the sofa so this is me taking he to her bad.

Interviewer: Are you the one who is taking your children to sleep at night?

Respondent: Yes, I am I want to help my wife by doing this since I will be at work, he whole day and she is spending her day with them so I want to support her.

Interviewer: Now we have reached at the last picture so again what did you see from this?

Respondent: So this was our dinner Genfo made from barley and it is good for its nutrition content so we are using it for preparing it for Genfo.

Interviewer: Since you have mentioned that barley has good nutrition content so what is the content of this Genfo or what did you use to prepare it other than barley and what are there uses?

Respondent: This is prepared from different cereals since we want to get from each of them and my wife is preparing it. And the content is more than 17 but I will mention it for you the ones I know for sure, the main ingredient is Barley and the other one is taff and third main ingredient is Rye they will make our bone strong and help for the child growth since they have different vitamins for growth.

Interviewer: Is there any floor prepared other than Genfo that you specifically prepared for your children's?

Respondent: No, we are using this preparation for different purpose like ''atmit'' and we will give them other foods like egg meat milk.

Interviewer: So, we have finished our discuses based on photos so I will pass to the other questions. From your society how did you define being men?

Interviewer: In you living area or community how did you describe binge man or what are the characteristics or what kind of behavior a man shows?

Respondent: Let me tell you my past life history when I was a kid in, I was helping my mom in every activity I can plus to this I come to Addis Ababa when I was grade 9 so since then I was doing all the cooking and other works by myself b/c I was living alone so this helps me to be familiar with it. Then I get mired I am helping my wife in all aspect like cooking washing closes and the like. In my living area the community understand like this now they are working and supporting the family.

Interviewer: You have told me that you are supporting your family and also you community also doing that.

Respondent R: Yes

Interviewer: Ok thank you so now let me ask you this, how did you and your community explain being father?

Respondent: Being a father is a very huge responsibility it has a responsibility to rase a children's who is going to lead the county so we need to shape them now in anyway like there behavior, knowledge and the like.

Interviewer: you have mentioned that it is the responsibility of a father to rase his children's so in what way and what are the activities on father needs to do?

Respondent: The responsibility of a father starts at the early stage that is when his wife is pregnant, he needs to go with her at her visit to hospital to lessen the massage the health worker going in that way he will support he in nutation and after the delivery he needs to support her to breast feed a child up to 6 months of age b/c it is very good for the health of the child and mother then after that he needs to support the mother by providing food both of them then when his children's go to school he needs to follow then closely and supporting them. When his children's get the stage of mirage he need to support then and give advice in how to support there family so over all I am describing the role of a father.

Interviewer: So, you are saying that we need to support our children at early age in nutrition, there behavior attitude in this way we will get a fruitful generation that will receive our county.

Interviewer: Ok that you I will pass to the 3rd question. What is your role in children's nutrition parenting? you have mention it earlier but can you please list your role?

Respondent: I am taking my children to school every day and my wife will take them back to home and when I get back to home, I will help them in their school work and I will play with them then finally we will eat our dinner together as a family then we will go to sleep. I am a provider for my home my wife did work now she is tacking care our children's so I am the source of money.

Interviewer: You can be done to improve your role in parenting? You have mentioned that you do not have much time to give to your family.

Respondent: I am working in government office and now I can not do much to change this but I wish if I have my own wok and give much time for my family.

Interviewer: If you want to explain more form our early discussions or if you want anything to add on let me give you a chance if not, we will pass to the next question.

Respondent: Let us pass I don not have anything to add on.

Interviewer: As you that we are now have covid in our world as well in our county so can you please tell me how did it affect you overall like economically or in any way?

Respondent: Since I am working in a government office it did not affect me in economic way but our social life decreased a lot previously we used to get together with our Nabors at the time of occasions but now we are not doing that even our children's did not go outside to place with their friends so it has a lot of impact in this way.

Interviewer: I have finished my questions you have given my many explanations for my questions but let me give you time if you want any time to add on.

Respondent: What I need to add s we need to give much time for our children's b/c they are going to receive our county

Interviewer: Thank you so much for you added home take massage and you participation on the interview and have a very good day.

Respondent: Thank you too and have a good day.

Father 7

I: Thank you for coming here and agreeing to be a part of this interview.

R: Thank you.

I: We will go to the first question. We have chosen 3/4 photos so this will be the first one. What stands out to you in this photo? What do you think this photo describes?

R: This photo shows me playing with my children when I am at home. When I am on day off.

I: Do you have day offs often?

R: On Sundays. And after I get home from work from 5pm to 6pm especially. I usually play with them during this time.

I: So, this shows that you, as a family, play together?

R: Yes.

I: Is there anything you want to add on this photo? Something that stands out to you.

R: No, this is it.

I: How many kids are there in this photo?

R: They are four. Five if you count me.

I: The eldest is your daughter? Please tell me their ages.

R: Yes. Ten, nine, four, and one.

I: Have any of them started going to school?

R: Three of them have started going to school.

I: What grades are they in?

R: The eldest is in fourth grade, following her he is in third grade, and the other one is in KG-2.

I: Okay. I will pass to the next photo. How about this, what do you see in this photo?

R: This also shows me playing with them in our surrounding area. I am watching my youngest daughter try to crawl. They were playing and I suggested that we take a photo together. I was teaching my youngest daughter how to crawl.

I: Okay. I will pass to the next photo. How about this, what does it show?

R: This photo shows me taking them to school. I woke up in the morning, they ate their breakfast, washed their faces and I am taking them to school.

I: Is their school close?

R: No, it is a bit far. They have to cross two streets everyday.

I: Are you the one that always takes them to school in the morning?

R: Yes, in the mornings.

I: How about when they return?

R: When they return, they come by themselves until they reach the streets, and their mother helps them cross. Their mother helps them cross the streets.

I: So, you take them in the mornings, and they come by themselves when they return?

R: When they return, they will not cross the streets by themselves. They stand and wait for her, she (their mother) knows what time they get out of school, so she meets them and helps them cross the street. The youngest one goes to a different school, so it is the mother who always picks him up from school. The two of them return by themselves until they reach the streets. The third child, she (mother) goes to his school and picks him up.

I: Are you the one that drops him (third child) at school in the morning?

R: Yes.

I: These are the photos. We will continue our discussion. What do the photos we saw tell us about your lifestyle and way of living?

R: (Long pause)

I: I mean your way of living, the area you live in, your interaction with your neighbors, and so on. Even though we can not see it in the photos, it is my understanding that you have a lot of neighbors.

R: We do not have a lot of neighbors, but we do not have a very smooth interaction with our neighbors. You live together with your neighbors, be it in peace or otherwise. It is a must.

I: How does it describe your lifestyle and what your way of living is? Is your home owned by the Kebele[1]?

R: Yes, we live in a Kebele owned house. I go to work and my monthly earning is not enough for my family, so I undertake additional work within our surrounding area.

I: Are you privately employed?

[1] Kebele: Kebele is the smallest administrative unit of Ethiopia.

R: Yes, I am privately employed. The salary is very low, so I do other jobs after I come home. I do jobs that are available within my surrounding like buying things and selling them at a profit,

I: Do you have a shared toilet?

R: Yes, it is shared.

I: What else can you tell us about your way of living? It might be useful to us.

R: This is what I can say about our way of living.

I: Okay. I will now pass to the next question. In the area you live in, how is manhood or manliness described? In your surrounding, if I ask someone or if I ask you, are there specific characteristics someone needs to have in order to be considered a man, what would those be?

R: I would describe manliness by hard work. Leading and providing for your family adequately, that is how I would describe it. There is no other way.

I: How about according to the society? How would the society describe it? What do men in your society usually do?

R: There are those who work and others that just spend their days in the area. People have different ways.

I: Please elaborate on that? You have said that you describe manliness through work and by providing for his family. When you say that a man should lead and provide for his family, how do you mean?

R: He has to work hard. If he has his own family, he has to work hard. He must show how manly he is in that way. As long as he has a family, he has to work, and he has to provide well for them. So, for me, a man works hard to educate, provide for, and lead his family. That is how I describe it. I do not describe manliness through fighting or dressing well, this is how I describe it. Manliness is described through work. As long as he has a family, if he needs to get an education, he should. Otherwise, he has to work.

I: So, a man has to provide for his family and be a source of income for the family?

R: Yes. If he is married, women should not be pressured to work; they should look after their children. The man has to provide for his family. It is his responsibility. Once he is married, she (wife) should take care of and look after her children while he works and strives to earn money.

I: You have described manhood very well. How about fatherhood? You have described manhood in a way, you have said he has to provide for his family. How about fatherhood, how would you describe it?

R: I would describe fatherhood through the love you have for your children and your wife. The relationship you have with your wife. Everything will not be fulfilled, you will make mistakes, there are a lot of things so solving them by discussion. Spending whatever time you have with your children.

I: As a father, how would you describe your relationship with your children?

R: I love my kids very much. As soon as I get home, I spend my time with them. Unfortunately, we do not have a lot of time together since I am at work when they get home, and they will be in their Holy Quran study when I get home, so I see them later in the evening. After that, we spend the time we have together.

I: You spend time together?

R: Yes.

I: Most of your children have already started school so do you help them in their studies?

R: Yes, but I am uneducated, so it is a bit difficult for me. But I help with whatever I can.

I: Please summarize how you would describe fatherhood. How is fatherhood described?

R: Being a father makes you happy. I am very happy that I am a father. I love my children and my wife very much. Other than that, I lead a very happy life with my children. This is it.

I: Okay. I will pass to the third question. What is your role in your children's feeding and caregiving practices? You have children so what is your role in their feeding and caregiving?

R: Like I told you, I do not want my kids to go hungry. I do other jobs because my monthly salary is not sufficient. Children demand a lot from you. Even small things that may cost 3 Birr. I do not want to say no to the small things they ask. I do not want my children to go hungry so within my ability, after I get off work, I do different jobs, even as a daily laborer, and try to meet most of their demands.

I: So, your role in their feeding is…

R: It is their mother mainly. She tells me what to buy for each child and I do that. Within my financial capability, I do what I can. If I do not have any money, then I will not do anything.

I: So, your role is by providing them the food?

R: Yes, my role is to provide. Their feeding is mainly managed by their mother.

I: Overall, what is your view/belief on children's feeding practices? What we believe and what we actually practice might be different but tell me your belief about it and what you were not able to do because of financial constraints?

R: Raising children is very difficult. It is very difficult. You can not meet all their demands. I somehow try to fulfill most of their needs, not all. I do not hold back. To some extent, I fulfill their needs.

I: What should their feeding practice look like? For instance, it is said that children need balanced diets to grow properly, this is what the health professionals teach. So, what should one father believe his child's feeding practice should be like?

R: If it was possible to afford an extensive set of things, that would be great. If you can fulfill all their demands, that would be good. But it should be within one's financial capacity. I do things within my financial capability. If I have the resources, then I will do whatever they want. When I do not have enough resources, I use whatever I have.

I: What do you mean by whatever they want?

R: Everything they want. When I do not have enough resources, I use whatever I have.

I: So, what you are saying is that you know the things they need but you can only fulfill them within your financial capacity?

R: Yes.

I: That is what I wanted to hear. For instance, a child has to eat protein rich foods and so on. So, what kind of foods are important for children? What is meant by a balanced diet?

R: For example, there is milk. There is breastmilk and cow's milk. It is great if they can have both. Besides that, there are various foods after they reach the age of six months. It is good if you can buy them balanced foods. But that is not affordable.

I: It is not affordable, that is why you do not do those?

R: Yes.

I: What can you do to correct this in the future? Or what can be done for you to rectify this situation? What do you believe can correct the things that you can not do now?

R: If I could, it would be great if I had a business of my own. I can do better for my kids. I can have a tutor for the children, to help them study so that they can do well in school. Not just their feeding, I want them to do well in school. I always think about having my own business. Something of my own because what I earn when hired is negligible. It will not enable you to lead a well fulfilled life. So, I always think about having my own business.

I: You believe that it will be solved in this way?

R: Yes, if you have your own business and if it is profitable, then you can better provide for your children. You can hire a tutor to support them in their studies.

I: Okay. Great. What do the photos show about your role? Is there something the photos show regarding your paternal responsibilities in their feeding and caregiving? If there isn't, tell me how you carry out your responsibilities as a father. Let us discuss about that.

R: Fatherhood, I already told you that I love my children, and I would love to do everything for them. I do whatever I can, within my financial limits. I never want my kids to be sad or to cry. I am striving.

I: So, this is how you describe fatherhood?

R: Yes.

I: As you know, we are amid the Covid-19 pandemic, and it had various impacts on the country and within each family. In your family, after the pandemic, what changes have you seen, socially or economically?

R: Yes, there are changes but I am still grateful to God. I, personally, do not follow the precautionary measures. It might be because of poverty, but I do not practice any caution when it comes to the children. But thank God, nothing has ever happened to my children. However, I get scared when they catch a cold thinking that it might be Corona.

I: What do you mean when you say that you don't practice precautionary measures for your children?

R: For example, it is said to take off your work clothes before you enter the house, but I do not do that, they all come and hug me when I walk in. I use public transportation, bus, and taxi, so I have contact with various people. They come and hug me before I have a chance to stop them. I have never been too careful with them, but God protects them.

I: How about your social interactions with neighbors, uncles and aunts, grandparents, siblings and so on? Compared to the interaction you had previously, are there any changes after the pandemic? What are those changes?

R: My mother lives just in front of us so we meet a lot. My sisters and their children live further away so we do not meet often. Due to corona, they are scared to come to us, but they come once in a while to visit our mother. Other than that, we do not meet often.

I: You used to meet more frequently before?

R: Yes, they used to come more often but now, due to the fear of Corona, they are afraid to use public transportation too. So, we do not meet, we talk over the phone.

I: How about within the neighborhood, for weddings or funerals, is it the same as it was before the pandemic?

R: Immediately when the pandemic hit, everyone used to keep their distance, greetings were made from a distance. But now, things are going back to normal with weddings, funerals, and other social events. No one even sits separately, everyone sits together, all mixed up. It is even hard to believe that we are amid a pandemic. Nobody wears masks and things have gone back to the way they were previously. Earlier in the pandemic though, everyone kept their distance. But now things have gone back to normal, be it at funerals or any gathering.

I: So earlier when the pandemic hit is when people were relatively more careful?

R: Yes, initially everyone was fearful, and no one went to funerals even. But now, things are reverting.

I: What do you think is the reason for things going back to the way they were?

R: I do not know. I am not sure if people are being ignorant. Even when being told about the spread and how many people are dying, people are not scared at all. I do not know how.

I: So, your social life has almost returned to the way it was previously?

R: Yes, it is going back to how it was.

I: How about economically, in your family? How about regarding your work, how was it before and how is it now after Corona? Where do you work? Is there anything that has changed economically? How about with respect to your income?

R: It has decreased extremely. Extremely. More than I can tell you. You can consider it to be zero. I currently in a private employment. We haven't had a raise for two years, even if he generates income or not. We have not had a raise since the pandemic. We work with the same salary. You can consider our salary to be zero. Two years is a long time. Especially for me, having to provide for my family, I am facing a very challenging time. Beside that, with regard to the additional jobs, it is not like it used to be previously, but I get work here and there.

I: You have mentioned that you do additional jobs when you get off from your regular job and are there any changes brought about due to corona regarding those jobs?

R: Yes, it has decreased. If people are not moving around, then business will be slow. It is when people move around that you can find work.

I: What is the job?

R: I work as a junk dealer. I buy scrap metals and jerrycans and take them to Merkato[2] and sell them. That is what I do.

I: So, due to Corona, there is limited movement?

R: Yes, movement is limited so work is slow.

I: So, your income has diminished?

R: Yes, very much. Even regarding our salary, we usually got raises every year but now there is no raise.

I: You have not even gotten a raise?

R: We have not gotten a raise for two years.

I: So, you have been using the same amount of salary you had for the past two years to provide for your family?

R: Yes, I use the same amount to provide for my family.

---

[2] Merkato: Merkato is a sprawling market in Addis Ababa, Ethiopia, considered to be Africa's largest.

I: I have finished my questions. Lastly, if there is anything you want to add, or anything you wish to discuss on, or if you have any questions, go ahead. I have finished.

R: I have a question. What is the purpose behind us taking these photos?

I: Okay. I thought I had explained it you initially. The whole study is conducted within Lideta sub-city. We asked health extension workers to provide us information about fathers living in Lideta sub-city who had children below the age of 5 years within their respective Woredas. That is how we found you. For instance, when I met you, I have explained the rationale behind you taking the photos, but I will repeat it again. The reason you took the photos is so that we can have pictorial evidence. Like I said, we, the research staff, are 3/4 people and the information will not go outside of the 3/4 of us. As soon as we are done converting the audio files into written reports, both the audio file and the photos will be deleted to ensure privacy. The main reason behind this research is to see what fatherhood is like, what the challenges are, what the gaps are, and what fathers are doing for their families. Instead of acquiring all this data from one person, we are conducting this research in one sub-city to present information. When presenting this information, we will not provide specific details of each participant, we will generally say what jobs fathers have, how many children they provide for, that they live in Kebele owned houses, that they do not have stable jobs, and what their income is like. We will not provide specific information. This is not the research ethics. The reason we take the photos, just like how it is said that seeing is believing, it is easier to see the way you live on the photos instead of just hearing it from you. It is so that we have a comprehensive set of data, there is no way it can impact you negatively.

R: Okay.

I: But like I told you, instead of just one person providing all this data, our responsibility is to do the study on a sub-city level and present it to the responsible stakeholder. Showing the gaps, showing the challenges, and to prompt on what should be done. This way, the acceptability of the information will be higher than any data collected from one or two or three people. I think you have understood me.

R: Yes.

I: If you don't have further questions, we will conclude our interview here. Thank you very much for taking time to come here. Thank you.

R: Okay.

Father 8

I: Thank you for agreeing to take part in this interview and coming here.

R: Thank you.

I: Just like I told you, we will be recording this interview. We will be discussing about family, fatherhood and what fatherhood looks like in your environment for the next few minutes. You have already shown us the pictures you took. Let us begin with introduction since we have not done a proper introduction. Tell me about yourself and your family.

R: I do unskilled work for a living. I am not permanently employed, and neither is my wife. She washes clothes for people. That is how we raise our kids. Even though we do not have much, we love each other. We care for each other. We have a good relationship with our neighbours and the community around us.

I: How many kids do you have?

R: I have two kids. There is five of us in the household including the woman I showed you in the picture. My two kids, my wife, myself, and my wife's sister. She (wife's sister) was a little sick, but it was not a big issue because she cares for herself. So, there is five of us living together for the time being.

I: Five of you live together?

R: Yes, in these difficult times (inaudible). Though it is not advisable to live in this condition due to the current disease, God protects us, and we live like this because we do not have other options.

I: Thank you for answering my questions. Moving forward to the pictures, we can see that you are dressing your child in the first photo.

R: Yes.

I: Tell me more about it. Why did you believe the photo shows fatherhood?

R: I say it shows my role as a father because when we were thinking about having a baby in the first place, it was to share whatever type of responsibility comes with it. I would dress him (the child), change his clothes, give him potty when he needs to defecate. I do not see how fatherly love is shown without doing any of those things. That is why I dress him in the picture.

I: So, this shows how much love you show to your child?

R: Yes.

I: What else? What else does this show?

R: What other thing should I say? What else is there that is more than fatherly love? Motherly love is known by everyone, but this is fatherly love.

I: What do you mean when you say everyone knows motherly love?

R: A mother shows love to her child because she carries her child for 9 months. There are some fathers who do not care for their children after birth. There are also couples who become close at first and later when the baby comes, they disagree on many things. It should not be like that, and God does not like that. They should have a discussion even before they have a baby. It should not be like that. That is how I think.

I: (Long Pause) People tell me that some fathers do not help like you do. How is a father seen in society? What is his role?

R: Fatherhood, according to society, is what is next to motherhood. Nothing stops a father from providing to his child if he is able to. If he (the father) he is not able to, there is nothing he (the father) can do. If the child asks for something and the father cannot afford it, he would not be able to get it for his child. But when a father can provide to his child, he will even to the point of giving his life.

I: I asked you the previous question because you said not all fathers care for their children. Tell me what type of fathers.

R: For instance, we see some movies. I think movies are made to teach people. We see many couples who are in love in the movies at first and break up after they have a baby. Even though these are not real stores, I think they teach people.

I: Let us talk about the next photo.

R: Okay.

I: What does this photo show?

R: This is a photo of him (the son) hugging me when I came home from outside. He would not have run towards me after seeing me if he did not love me.

I: You told me that you have two children. I can see that it is the same child that is in this photo and the previous one.

R: Yes.

I: How close are you with her?

R: Is it with my wife?

I: No, I meant with your daughter. I did not see her in the photos and the two you showed me are with your son.

R: She is older than my son. That is why she is not in the pictures you have seen but we did take pictures together. My kids are equal in my eyes. My son is much younger. Therefore, he gets the affection young children should get. Apart from that, I love my children the same.

I: How about this one?

R: I was eating lunch and I asked them to eat with me. They told me they already ate, and they were playing. They play on the bed because we do not have any space for them to play. We also eat on the bed. If there are two or three people over, two people have to eat while sitting on the bed and the door has to be closed or else there would not be much room to move. So, they are playing in this picture. My daughter is old enough to know the picture is being taken. That is why she was facing the camera.

I: How about this one?

R: This is their mother when she was feeding them.

I: What does the picture tell you?

R: I feel happy when I see it. She is their mother. When she feeds them and to see them eat makes me very happy. It is very nice when they eat whatever is made happily. This is a picture of my son and I when I was trying get him to drink water and he was attempting to drink by himself without my assistance. He was on the bed, and I was telling him I must help him, so the water does not spill but he wanted to drink by himself.

I: You are playing in this picture?

R: We are playing in this one and on this picture, we are eating breakfast. This is my wife; this is my daughter who is older, and this is my son. We are eating together.

I: What can you tell from the picture?

R: It shows love. It shows how much we love each other. I feel happy when I see a picture like this because I realize how much we love each other. My daughter knows how to count, and she is teaching him (her brother) by saying 'ha' (ሀ - the first letter in Ge'ez script). If there is something she cannot read, she asks me, and I tell her. The young one (son) is very young by the way. He just sees and I do not think he is old enough to learn.

I: How old is your daughter?

R: She is eight. She will be nine soon.

I: Does she go to school?

R: Yes.

I: You told me the photos you took show my role as a father and how much we love each other. How do you describe men in your community?

R: I would describe them as......I have not met any father who does not love and raise his child. If I do meet one, I will try to give him advice when I can. There is nothing I can do if I can't. If I tell a father what

to do and he should love his child and he does not take my advice very well to the point a fight might break out, I will not say more. I would consider reporting to an entity that deals with children's issues.

I: I do not mean in with regards to raising children. I meant how do you describe being a man is seen according to society?

R: I do not understand the question.

I: Have you ever thought about what society expects men to be and what roles they are expected to play?

R: It should be the same in social aspects and such. I do not know what to tell you. Whether a person is rich or not, everybody in society should see people equally.

I: For instance, what types of roles do husband and wife have in the household? What is the role of the mother and what is the role of the father?

R: Do you mean in the society?

I: Yes, from what you observe. You told me about how it is in your house. Do you think it is the same society wide?

R: The roles are similar in the compound we live in. If the woman is the first to arrive home, the man will do whatever needs to be done in their home. She also does the same. The roles are the same in the compound.

I: Therefore….

R: There is a good social life.

I: There is no such thing as a person should do specific tasks because she is a woman or a man?

R: No such thing exists in my community.

I: How about in other communities? Have you noticed that type of thing?

R: I have not experienced such a thing.

I: How about as a father? What changed for you when you became a father?

R: A lot of things changed about me after I became a father. I go home early. I worry because I lead the family. I work hard and I do not choose what I do. I do whatever is available. So, these things changed.

I: What do you think caused you to change?

R: It is my kids that brought the change. Since I am married, I think marriage is being able to lead a family. If I am somewhere else when I have wife and kids, it is not marriage. I have benefited in a way. I used to eat out when I was living alone, and I usually did not feel well after eating because the food could be a

day old and so on. Now that I am married, thank God, food is made at home, and we eat what is available at home. That is good. Those things have changed.

I: You have shown me when you were feeding your children. What is your role that is related to childcare?

R: I like to care for children as much as I can. The do not necessarily have to be mine though. It could be a neighbour's child or someone else's who lives close by. I treat kids the same way. I do not treat a child differently because it is somebody's. Because kids are young, the do not know anything. So, it is necessary to explain it to them.

I: What is your role in how your children eat?

R: When it comes to diet, my kids eat the same thing that I eat. My son is three and there is nothing special I buy for him just because he is young. That is because we cannot afford it. If we eat shiro, he will eat the same with us like an adult. That is because I do not have the money. I would provide better if I had money. We are not permanently employed as I told you earlier. Therefore, we are living from hand to mouth.

I: Are there any people you look up to and try to learn from based on the information they provide when it comes to your children's nutrition?

R: If anybody tells me to do specific things with regards to their diet, I will not hesitate to follow that advice. It benefits mine and my children's health. I will be happy to do the things they tell me to do.

I: Are there any people who know a lot about children's diet?

R: Yes, there are people who tell me how they should eat and what to buy. I am not permanently employed, and I work only when there is work available. It is summer now and there is not a lot of work as a result. My wife washes clothes for people and she is in a better position now. Therefore, I will not be able to provide what people suggest I buy for my kids. But I try my best to provide for my child.

I: If a person asks you what fatherhood is, what would you answer?

R: Fatherhood gave me grace. I see it as I was born again, like I got a brother and a sister. I do not know how to describe it. It is a big thing. It gave me grace.

I: The COVID pandemic created a lot of disruptions before everything started to go back to normal. The disruptions were in work, social life, and many things. How did that affect you?

R: It has affected me in many ways. I was working in construction and that stopped because of COVID. That is how I was affected but I thank God that I am healthy. We also hear the ministry of health recommending to physically separate ourselves. However, that is hard to implement in our house. My wife even decided to no longer wash clothes for people. We did not have anything to eat because of that. It has disrupted a lot of things.

I: How about in your social life?

R: Social life was good, but my wife washes peoples' clothes and people thought they could get infected through clothes. Therefore, people were reluctant to have my wife wash clothes for them. My social life has been affected in ways like that.

I: Are there any additional impacts on your and your family's life due to COVID?

R: There are not any impacts in our lives apart from a decline in our income. Although it is unskilled labour, it used to be better before COVID. A lot of things are closed nowadays. For instance, the construction company I used to work at laid of its workers and it was hard to find a job was not easy to find a job somewhere else. Our health is not affected though.

I: No, I was asking if you had attempted to get a job at a different company and not health related impacts.

R: I do not have skills and I do unskilled labour work. So, nobody hires me. Maybe they hire those with experience, but I do not have that.

I: We have discussed about various issues. Do you think there is anything we forgot to talk about? Anything that makes you think I did not ask you it when I was asking you about fatherhood?

R: There is nothing you did not ask me, or we forgot about.

I: You told me you are not working right now. Can you briefly tell me what you do after you wake up in the morning and what you do with your children?

R: I wake up early in the morning. Because there is a girl who helps me, I help my kids wash their faces. The older one goes to school every other day. If she is there, I help them wash their faces. There is a bakery next to where we live. I buy bread, make some tea and I feed my children.

I: So, you make them food?

R: Yes, I do.

I: Do you always do that?

R: I make them food if I am home.

I: (Inaudible)

R: I meant tea and things like that.

I: No, I was asking if you make them food.

R: I make Shiro and so on.

I: How many men know how to make it?

R: What?

I: Men usually do not know how to make food.

R: I know how to cook. I do not have an issue, but the onion burns me a little. I cook without an issue. There is nothing I find to be hard. I cook and serve it for them.

I: The reason I ask is because I want to know if anyone of your friends helps around the house like you do.

R: In our house?

I: Think about your friends and tell me how they are.

R: What do you mean when you say, 'your friends?'

I: You have male friends, right? Do they help with tasks at home like you do?

R: Yes, they do. I have one friend who washes clothes when his wife goes to work. He cleans appliances and so on. I learned actually from him. I went to his place when I was not working, then I saw him doing the dishes. So, he is who I learned from. I do the dishes after we finish eating if my wife is not home. If she is home, I try to help her with what I can.

I: You help each other in your home?

R: Yes, we do. We do not say this task is only yours and that is mine. Our kids learn from us. We do everything equally and there is no division of labour in our home. Both of us do any task that needs to be done.

I: Since you told me you are the ones who teach your kids, do you show them how to do chores?

R: I did not mean teach them in that sense, but they see us doing work and they will be like us when they grow up. We do not tell them to do chores.

I: I did not mean if you ask them to do chores. You have a son and a daughter. To make sure they grow up seeing one thing……

R: Yes. I do the dishes, so the boy does not grow up thinking he does not have to wash dishes because he is male. If he sees me doing the dishes, he will at least wash the plate he eats from.

I: When I asked you if there are people you look up to, that is what I meant. Continue where you left off. Tell me what you do after you feed them their breakfast.

R: We live near Lideta church. We are Orthodox Christians. I take them to the church after breakfast and we stay there for a bit. We play for a while, and we go back home. Her sister is with us. Sometimes she goes with us and sometimes she does not. We spend time there and go home afterwards.

I: And in the afternoon?

R: I go out in the afternoon and try to find any job. By the way, I do not have preferences when it comes to jobs. I do anything. If it is carrying things, I do that. There is not a type of work I do not want to do as long as I get paid. I am not in a position to choose work because of how my life is.

I: So, do you show your kids you love them by spending a lot of time and playing with them?

R: If I am at home, yes, that is the case.

I: (Long Pause) Thank you. These were the questions I had for you. I give you the chance to add more if you think there is something I forgot or did not talk about.

R: There is nothing I would like to add but you did not raise about the house or is it because it does not go with the topic our discussion?

I: What about the house?

R: The house is very small. You asked me questions about health. I mean firstly, even if the food is made properly, it will not be good if the space that is next to you or environment is not good. There is a pipe here like I told you earlier. We do not sleep if it rains at night. If there is heavy rain at night, we do not sleep. It overfills. It overfilled one time and it got into the house. We do not sleep if it rains because it gets in the house. I will be happy if you see that. There is nothing you forgot apart from that.

I: Like you said, we will take that as something. If our (inaudible) explained it to you, our focus is on children and their nutrition. That is the reason why I did not ask about the house. We will do everything we can to relay the information to body that the issue concerns.

R: Okay.

I: The only person you did not take a picture of is your wife. What is her role? In matters related to childcare.

R: She is better than me. She comes and at times when I am working, she feeds her kids even if she is tired or hungry. She could be hungry, but she feeds the kids first. She eats only after the kids eat. She does that even when she is hungry. She is better at caring for the kids than I am.

I: In what aspects? Is it because the makes food for them?

R: Yes. Because she makes food for them. She also loves them as a mother. That is good.

I: How would you describe the love she has for them as a mother? You said that it is known repeatedly earlier.

R: I say that because she carries a baby in her womb for 9 months. I am seeing it from that angle.

I: Is a father’s is less?

R: The thing that is less is the social part of it. A father’s is not the same as a mother’s though.

I: How is it different?

R: Doesn't a mother do a lot? She carries for nine months. The food she eats may give her upset stomach. Aren't there many things? A mother goes through a lot.

I: Is that what differentiates it?

R: Yes, that is what differentiates it but after babies are born, it is the same.

I: After they are born, is it (inaudible)?

R: Yes, after they are born, it is the same. It is equal. She is different in that she breastfeeds. Breastfeeding is where the difference is.

I: Did your wife breastfeed both of your children?

R: Yes, she did breastfeed both of them.

I: For how long did she breastfeed them?

R: The older one was breastfed for two years and something. With the younger one however, it stopped recently. It ahs been three or four months. He was breastfed till he was three years old.

I: Oh he was breastfed for a long time.

R: Yes, it is a long time. There is a gap in their ages. The older one is nine years old and the young one is three. There is a big difference between their ages.

I: It looks like you planned it well.

R: Yes, but our life is……

I: Where do you get the information related to (inaudible) health and food?

R: We get it from the media, and they also talk about it in advertisement. We hear about it like that.

I: How about health centers or do you meet with health extension workers?

R: Yes, if it is a health-related issue, we go to the health centers because it is close to us. We also ask for information when we go there.

I: Is there not anyone wo teaches you about the current information?

R: No, there are not many people like that.

I: Do you ask when you go?

R: Yes, we ask for the information when we go ourselves. A person does not usually go to the health centers unless that person is sick. It is because we do not have much (inaudible).

I: I think you go there a lot since you have a lot of information. I say that because that is where you get your information from.

R: I hear about it when I go and when I see it in advertisements and the media.

I: What are the reasons you go there for?

R: Do you mean the health centers?

I: Yes.

R: I do not go there if I am not sick. I get into accidents a lot and we get pricked by nails when we work. I go there for those reasons.

I: Do you not go there just for the sake of getting information?

R: We do not go there for that. If we do not go and we do not get it form the media, there is nobody that comes to us to teach us.

I: What types of information do you get from the media?

R: The Ministry of Health makes announcements regularly after COVID came. We hear about how we should protect ourselves from the media, how many times we should wash our hands and so on.

I: Have you not gotten any information related to childcare and nutrition?

R: We go there because the boy has to vaccinated and we learn about that when we go there.

I: What type of information do you get?

R: They tell us what he needs. They tell us he needs a balanced diet. We accept what they tell us, and we go home. He eats what we eat so we give him what we have because we cannot get what we do not have.

I: Thank you very much for taking your time to have this interview with me. If there is not anything you would like to add, we will end the interview now.

R: Thank you so much. I feel my problems are smaller after talking about them. May God repay you.

Father 9

Interviewer= **A**

Interviewee= **B**

A: Good morning, First I would like to thank you for being willing to be interviewed. As I have mentioned the last time we met, the information you provide us is kept confidential and secured. After we are done with the study, we will erase the information you and other people provide to us. Since we couldn't find the photos, I will ask you questions without including the photos. So, from your perspective How do you describe fatherhood? What is fatherhood for you?

B: (Phone ringing) Uhm... Being a father… Is happiness.

A: How do you describe fatherhood?

B: How…how can I tell you?!um (deep breathing) being a father is challenging. Quite challenging to be a father. The responsibilities are many including you have to compromise your job but it is enjoyable and has its own taste.

A: How do you describe its hardship?

B: Its very hard with a child especially when you are young and have a child whilst you don't have understanding and maturity. When you are young and are supposed to work but rather having a child is quite a hardship.

A: What are the things a father can do for his child or even you as a father if you can tell me what things do you do for your child?

B: Uhm...Most of the time you spend time with your child (you spend time with her), they will ask you questions... many questions sometimes they ask you questions beyond your mindset. They need mother they will ask you. You manage to tell her based on what's suits her and her level of understanding.

A: Uhm…What is your role related with your child growth and nutrition? What kind of things do you do?

B: Uhm...i am the one who cook and feed her. Well, we do eat what we have, but for her I serve her the better and different foods in the house that are useful for her. Most of the time, we eat vegetables and fish.

A: Aha Aha…what kind of different foods? You told me, you give her different kinds of foods like you eat vegetables most of the time, If there are any other additional foods specifically you give for your child?

B: well, we go out on Sundays to city to enjoy, we spend the day relaxing, we go to "wemezeker" and we read and the like. She is clever. Very clever child. Her mind is... (interrupted by the interviewer)

A: How old is she?

B: She is 4 years old.

A: She is 4 years old, okay.

B: Yes…yes

A: Okay, therefore, on her nutrition you told me you give her vegetables to eat?

B: Yes, vegetables often

A: What else?

B: Fish…Fish and we eat others like what you call green things

A: What benefit does it have to give such kind of foods for children?

B: Because I give her this first, even her mind becomes very fast to me. Her mind becomes fast to me, she has no illness. Thanks to God she has no illness. She has no illness.

A: Uhm...uhm...Okay. So, it prevents from illness?

B: Yes, its ability to prevent is best.

A: What else? What kind of contribution do you think it does? You told me the types of food you provide her often are fish and vegetables, by eating fish and vegetables often, what benefits do you think she gets?

B: By eating fish and vegetables, first, her mind becomes fast for me. Second, she is healthy, she has no illness. Her wellbeing as you can see, is good. Her looks. she is very pleasing.

A: Okay. On child care and nutrition, what kind of responsibility does a father have?

B: He have to follow thoroughly. A father must follow thoroughly.

A: How? When you say he has to follow, try to describe it?

B: From morning, from morning when she rises up to brush her teeth, you have to shape her by what you are doing. If you do not shape your child at this time, she will be escape, she will get used to improper things. With me she will do what I do in the morning. She even ties my shoes when I go out in the morning.

A: What else? Well about her growth you told me you follow her from the root

B: Yes, church... she goes to church

A: Okay. She goes to church.

B: Yes, I take her to church.

A: Does she starts school?

B: Yes, she is in KG 3.

A: She is in KG 3.

B: Yes, she is now passed to KG 3. She got the first in her results. Yes, she is in this picture, when she got rewarded.

A: Ow...Aha...Aha. Okay, so do you think her nutrition has helped her for her current condition?

B: Yes, yes nutrition is crucial. Nutrition is very crucial even for adults or anyone, food is crucial.

A: Uhm...You told me you shaped her growing, you go to, sometimes you go out on Sundays and church. Is there any other thing you do with your child?

B: Other. Uhm... this is it we go to church, since it is fasting time" some subae" we are fasting. She does not fast but I fast then we will go to learn in the evening. On Sundays we will go out, we go to stadium we do things like cycle and other things. We do anything that I can afford and create something that makes her happy and because I will be happy when I am with her.

A: Okay, in the area that you are living now, how is being a man described? Now you told me about fatherhood its goods and challenges and its many responsibilities. How is being a man described in your area of living?

B: I was raised as a man and woman since I lost my parents 13 years before in my childhood. I start to live my story when I was 16 years old so I don't know the taste or feeling. I told you before the hardship of fatherhood. For the question about description of men in my village most of the time…often since I started to work on my childhood I don't know about the life in the village. I go out and go to work... (Phone ringing) I go out and go to work. After work there is my sister, she helps me out to look after her. Even now three to four days back i send her to my sister who lives in Akaki and who have children.

About the question you asked on men, men are hardworking, all men in my village are clever. Maybe it's the condition of the area, everyone tries to survive, to come out in a different way since there is competition in the area which is good.

A: uhm... so, lets generalize it and how do you describe being a man? Is it by doing work? what is it for you and the society?

B: Being a man, you do your work. What makes it different is nowadays in the 21st century, is the time were men and women are doing the same kind of work so we are working and living equally. The only thing you differentiate as male and female is via our sex, there is no other thing that makes it different.

A: Is there any work specifically for a men and women?

B: There is no such thing. We do all the work.

A: Okay (Phone ring) For example in the family, does a man serve food or does he provide money, is he the only one who go to work, what does the society think regarding this?

B: In our area a man goes out for work and come back, he works and get back. But now women and men all work. If you come at day time you don't find anyone. There are only youth live in our area, no elderly but area of the young. Most of them are not alive, they passed away. Some are moved due to development and condominium and its difficult to find anyone. But we may find each other during church travels, social gatherings, for buhe and another holiday. Otherwise, all men and women go out to work.

A: Okay. First, I asked you about your perspective on fatherhood. Now, what is the perspective of the society in your area on fatherhood? How do they describe fatherhood?

B: Uhm… Fatherhood... (paused 1 second) they will describe it the same as I do. I don't think they will describe it much better than I did.

A: Fatherhood includes spending time with children…

B: (interrupt the interviewer) Of course yes. If you go and see on Sundays, everyone brings their kids on Sundays although you didn't find one, almost all have children who are 3 to 4 years of age. You may even find 6 months to 1 year of age since most of them are newlyweds and had their kids while they are young when i say young, I mean 23, 24 to 27 years this includes both men and women. So, I think they will describe it like I did.

A: Okay, umm...what is your role on your child care and growth you mentioned that you are the one who cook and give it to her. Do you do cook and feed her always?

B: Yes, I am the one who cooks. I am a very good cook by the way.

A: Umm...that's good, so it starts from cooking?

B: (interrupt the interviewer) of course, sometimes it seems like, life is all about cooking, since you start cooking in the morning and also you cook in the evening so you always spend time in the kitchen.

A: So, you are the one who cooks and feed her always?

B: Yes, I am the one who cooks her.

A: So, your contribution starts from first to the end? you buy also.

B: Yes, starts from in the morning and of course I buy and work. I work my own business. First, I rise up in the morning, I will cook and we eat and the leftover, if she has class or when class starts i will send it to her for her lunch. During lunch I may have lunch outside due to work but we always eat our food at home.

A: Overall, you told me about your child's growth, what does your role looks like in your family generally?

B: My role in general is managing the house like providing what's needed, doing what's needed to be done. I mean all the time including working, cooking and taking out my daughter I am the one who do all the work in the house.

A: So, your overall responsibility looks like this?

B: Yes.

A: As it is known this is the time of Corona, so during this time our country and the world are facing many changes. In your family what types of changes do you encounter?

B: We were hurt psychologically, very much hurt at first. Since we didn't have much understanding about it at first, we were shocked. So, the first thing we did was to buy and collect foods to eat and then we stayed at home. Did not connect or meet with people me, my daughter and my elder sister. Me and my elder share on room we were very afraid to even connect with each other since i was the only one who goes out from home. So, it did affect as psychologically. Secondly, we were highly economically turmoiled but thanks to God it has passed already.

A: How do you describe the economic turmoil? What types of work did you do at first or is that work closed?

B: There were no one who could make me work or there were no one who come to my work place, no customers were around then there comes a time where everyone stayed calm and in a serene spirit. That were the time were everyone stayed together to help each other out and in committee even we were able to see the old people. So as I told you there were no work like it stopped completely stopped like a watch. This has continued for few months and you know we have to eat, so we tried to go out, call and connect with people that we know who can help us work and search

for a job though everyone told us they are also in the same situation but there were also some good people who advice you and help you to work. Then I started to go out for work.

A: What type of work did you work?

B: I work Generators, maintenance job and electric lines, pump and mixers and most of the time machines in general but even these jobs were not available at that time. Hotels as well as the companies that I know were not working at the time because there were lock down no one can transfer or use their money so you don't expect money to get at any time.

A: What types of influences does corona bring in your social life?

B: We used to gather for holidays, to buy things, to meet up anyway it was so scary. It was the life that we all went through but it was a bit scary. We were like this even with my neighbor to prevent each other. We used many prevention methods like we used plastic covers on doors handles and pipe handles. We use public toilet which makes it difficult, used sanitizers and soaps and the like. Now we are not like before we become little carelessness and forgetting those things we did or we are getting used to it I don't know.

A: If you start it already, you told me that you showed carelessness to those things you did you mentioned, what types of things were they?

B: Mask, sanitizers, I used to have sanitizer and carry it in my pocket where ever I go. Now I don't have one until I used it now for this interview. Even, I used to have it in my house. I use to wash my hands frequently, used to search water whenever I touch something I am not doing this nowadays. But better than me, my daughter is very cautious, she even warns people but no one listens to her. May be the school has taught them very well, until now it's all shaped in her head.

A: So, you are also reluctant?

B: Yes, I am very reluctant towards it.

A: What about the society?

B: Of course, everyone forgets about it. I don't know. Maybe we are getting used to it. I don't know what kind of spirit it is that we all forget about it this quick. If you go and see everyone, they forget about everything. At first, you don't even shake hands for that matter and now we greet and kiss each other.

A: What kind of harm do you think it caused?

B: These days, Corona is increasing from time to time. I used to think that If we obey the time set or the time frame that is declared by the government and listen the advice of health professionals, we might control it from the beginning but we couldn't the reason is people are reluctant and we

have lost many people due to our reluctance. The social gatherings like going to funerals by saying “no problem” has sacrificed as so much.

A: Previously, you have touched a bit on children’s nutrition and you told me that you often eat vegetables and fish, can you mention me more other food types that are useful for children? What is their benefit?

B: I don’t know why I like fish very much

A: what others foods?

B: I cook some of the foods that are easy to make call its lentils and we eat beans and other cereals at nights when we watch movies. I like vegetables and ‘Shiro’ very much but most of the time we eat vegetables.

A: You told me that it prevents her from illness and it increases her mind maturity and growth. What other benefits do you think she gets?

B: These are the benefits I mentioned and I want it to grow on her that she eats it in the future as well. We all know from education or else that eating vegetables prevents from illness. In addition, eating fish its inner substance is useful for the mind and cognitive thinking. I prefer my daughter to eat foods that are useful for her than those that doesn’t benefit her.

A: Okay. Thank you. May be if you want to add something that you think is not covered or if you want to explain more from previous points that we have talked or if you want to add something I will give the chance to you.

B: Thank you. Uhm…the thing that I want to add from what you mentioned that makes me see my self is about the prevention. I was shocked when you asked me about it. The spirit that is in me and hers is not the same. I think about this thing that if I am becoming reluctant and try to disintegrate it, I think she will do the same so to not transmit this habit to her. So, right after I go out from this studio, I will be cautious and I will be happy if people try to be cautious as well.

A: Okay. Thank you very much as I mentioned before, thank you for coming here and give as your time.

B: Thank you, let you be in peace.

Father 10

Interviewer: Frist let me introduce myself I am _________and I am a member of this study and we will be interviewing you with ______ she is also a member of the study. I would like to thank you for your participation in this study. As per our discussion I have tried to tell you the aim of this study but let me remind you again. So, the aim is to see the role of fathers in the child parenting and nutrition shortly. And all the information you will be given as will be kept securely and will not share the information with their other person outside of our study group. Thank you have also taken pictures for our discussion and the pictures are more or less similar so we will tart our discussion based on them first then we will ask you other questions as well. The interview may take approximately 30 min and will be using a recorder. As I have mentioned it before we will not share the information you will be giving us today with anyone outside of the study goop and even for us, we will delete it after completing our report and I would like to reassure you this point.

Respondent: Ok

Interviewer: So, we will start our interview by this picture what did you see on this picture. As we can see you with your child tell us about this picture. Please relate your living conditions as well.

Respondent: Thank you for your invitation to participate on this study. My name is _____ and I am living in Ledeta sub city. This is my son and his name is ____. And he is 1yr and 8-month-old. This morning his has eaten his breakfast early and I was trying to feed him buy he was full so I and not so I give him milk and he is drinking that so ____will spend some time in a day with me. He is very close with me even more than his mom b/c he is working mom and she is now working at Agency. So, I will spend time with him until I go to work. He is close with me like I am his mom if you can see other pictures, he is playing with me but I cannot take picture the moment we have.

Interviewer: Ok what did you see the most on this picture?

Respondent: On this picture I am feeding my son and he is happy. And we are at our home seating on the chare and he is seating at me. He is drinking a cup of milk.

Interviewer: OK how this picture is related to your society living condition? Or how this picture explains your live? As we can see this picture also show us your home so, please relate this and you're your society i.e. wereda 09 and tell us about it.

Respondent: Yes, I am living in a very difficult room and you cannot even work on it. I have spent much of my time in this room. And I have lived here for 11 years and it is a rent home. For multiple time I have requested our wereda for home but they have given me the response that they will give a priority for peoples living on the streets. My first son has an accident on his leg because of fire from stove so I have even mentioned this event for the responsible persons on the wereda but they did not give me a response. I have started my school from grade hear and I want to thank GOD I have finished my collage by accounting last year. Most of the time I will not give a place for problem in my life I was eating bread with tea when I was learning I have meet my Goals but I am steel left with some I am working to full fill them I am working in a Daily labor and I have mentioned on our discussion earlier that my wife she is working at the Ethiopian commercial bank so we are dependent on her income and plus to that I will support her if I get a work on the daily basses since I don't have a stable work since last year I don't have

any work this is how we are living. Our home as you can see it is very small and do not have space we are from poor family and my brother is currently living we us and he will start his school hear from grade 9 and he is working like me so we are 6 in total.

Interviewer: Is this the same for your society or it's different?

Respondent: In our village as I told you it is all depend on the time you come to Addis for some of them, they are living in the home given by the Kebele and little are living on the rent home they may have a problem on the size of their home but I am not sure.

Interviewer: You have told us that 6 peoples are living in this room and you also told us that your brother is also living with you who else is living?

Respondent: My uncle daughter she is 13 by age and she did not speak Amharic and he is helping us with our child.

Interviewer: You have told me that society living condition and I would like to ask you about your toilet, are you sharing that with your Nabors? Is it comfortable for the children? Please tell us a little bit?

Respondent: Yes, we are sharing the toilette for 20 peoples and it was constructed by the organization and it is very difficult to use but now we have modified it and we are using it now.

Interviewer: Ok, thank you I will pass to the next question in your society how did they describe being men?

Respondent: Please repeat the question

Interviewer: Sure, I will repeat. In your wereda 09 how did describe being men?

Respondent: The social life in our area is very nice they are not discrimination on the work and they are helping their wife and even me I am helping my wife in home activities. If I come to home early, I will cook a meal for my children's and I will feed them and I was living alone when I was learning so I was cooking a meal for me so this is not new for me. The other peoples in our area are also giving care for their children's and even they will not go to their work if they don't have any one in their home that will help them to give care for their children's so this is what I see.

Interviewer: So, what is the role of the men in child parenting and feeding?

Respondent: In our village the peoples are living poor but child caring is not related with economy they will work every work they get and they will give much attention towards their children's. They will do everything for their children's and they have a thinking that they will live a better tomorrow if they are rising their children's well.

Interviewer: What did they do for their children?

Respondent: Fathers?

Interviewer: Yes, for example you will have a work to do and also attend the social life so at what time did you give for your children's and what activities you do?

Respondent: Yes, it is different on our economy status for example some fathers will buy a bicycle and they will teach them on how to use it some will take their children to the park for refreshment and for those will less capacity they will buy something and spent their time at their home, so it is different what I do for my son may not be done by others. I really love my children's a lot and I do not have anyone I love better than them. I was young when I have my first son so I love them a lot.

Interviewer: Both of them are boys?

Respondent: Yes, they are

Interviewer: You told us that the thing you do for your children's is different and some may not do it so what did you do for your children's.

Respondent: For example, I will cook for my son by purchasing something that he will like for the shop so I think some peoples will just participate in providing the money but not in cooking.

Interviewer: How did you see the role of the father in for society is it the same as you or it is different?

Respondent: When I come to Addis for the first time I see a lot of children's do things not expected by their age like they my use cigarette and others so when I see that I thought the norm was the same us our region and I go to the children's and told them not to do that and even I have punished them and the next day there family come to me and kick me and told me punishing them is not my business so I have stopped that but in our region society will participate in children's parenting my mom and dad is not only punishing me the society will also.

Interviewer: So, you're saying the children parenting is different from the regions?

Respondent: I am not saying that all the children's are like this and as I told you before I have lived in this village for 11 years since I come to my region and some of the children's have this unwanted behavior they may not be even living in our Nabors but I have noticed this so this is the fault to their parents if they follow their daily activity and force them to go to school so the children's will not do this kind of activity by this age, My first son is 11 yrs. now if he has time I will take him to church so in this way I will protect him.

Interviewer: I am not sure which regions you come parenting is not the responsibility of mom and dad the society will also participate is it the same hear?

Respondent: Let me tell you my experience I come to Addis when I was 16 yrs. old and when I was living in our village in my region. I was going to school one day and since I have time I was planning by jumping and I did not the person before but he is living in our area so he come to me and punish me by using stick b/c I have Rougned my cloth so I go back to my home crying so I told my dad about it and he said it is good and he also punish me again this is also true for other children's even they will come to school to tell to our teachers what we have done so I come to understanding that peoples are also involved in child parenting. I take this lesson and when I come to Addis, I saw children's playing on the street and when old peoples passing, they kick them by the ball so I go to the child and I have punished him on his hand and he go to his father and they come with the family members they wear 3 and they hit me and after that I will do this. So, I come to understanding that the culture is different from our region.

Interviewer: So, it is different?

Respondent: Yes, from my experience it is very different. My expense in my region and hear is totally different for example we have respect for peoples but hear they will judge you by your close this is how my understanding it may be different for others.

Interviewer: Ok thank you for your explanation and I will pass to the next question. Previously you have mentioned a little bit that when you come home if your son is hungry and did not eat you will go to shop and something that he loves and cook for him and feed him so can you please tell us about child nutrition and what is your role?

Respondent: On child nutrition and feeding I will do on my economic capacity. My son will drink cow milk contract every day I was raised like this. In addition to this he will east packed foods like ''Indomin'' and other non-packed foods. When he gets older now, he has started to eat Injera so I give an attention on his foods. I have remembered one situation he gets sick one day and I was so worried and I took him to nearby health center he was nauseous and we stated overnight there and I was thinking I was at work and he was with my uncle daughter she was taking care of him that time so I was thinking he puts something on his mouth so I go to the doctors and told them to check on his stomach as if I know better than them so they told me to stay calm and they give him Glucose by his noise and then after midnight around 8 local time we go to home by using contact taxi. So, after that I will tell he to give much attention she is 13 by age and she will obey what I told her.

Interviewer: Ok earlier you have mentioned that your son is drinking milk every day so what are things did the children's need to eat?

Respondent: I am familiar with child nutrition from education. They need to eat carbohydrate, protein but I cannot incorporate all in his diet b/c of finical constraints. We are using my wife income for all purposes like house rent my son school fee and our monthly meal expenses so we are trying to feet on the money we have. So based on what we have I will try to feed him eggs, beans like Shiro, bulla like ''Genfo'', peans, rye in combination with other cereals he was eating most of his time after 6 month of his age and the like.

Interviewer: you told us that you wife will prepare his foods so on your child nutrition who plays major role?

Respondent: She plays major role in food preparation but something I will give the foods ideas like if he eats something today, I will tell he to prepare other thing for tomorrow in even I will participate in feeding him. Even I have cow milk contract or rent for a month so he will drink every day. So, we are working together.

Interviewer: As you know that now we are on the COVID era and at this time as a country we are on different changes and as your family what kind of changes you faced?

Respondent: If I am not mistaken now, it has been almost 2 Yrs. Previously, we used to pray collectively with family and friends and we have also prepared a place we can wash our hands but peoples are neglecting this activity now but steal I am doing it but I cannot change my close when I come to my home from work b/c our home did not have a space for that and if my son see me coming, he will run towards me so I cannot do it. So, what I do is wash my hands and change the mask I use outside my wife is also doing that. She has a better attitude more than me b/c she is spending much of her time with peoples.

Interviewer: Is there any change on your working condition?

Respondent: Your asking about my wife working condition or mine?

Interviewer: Both

Respondent: Regarding my working condition after COVID is that now I am working as a chemical spraying for the client's office, company, home and the like. We will spray chemicals. They give us instructions or prequestionnaire measures so we are following that when we work. But before covid I used to work in construction sites and related areas.

Interviewer: Yor are saying that the working opportunities and more now b/c of COVID spray activities or is it less?

Respondent: B/c of COVID In my understanding it gives me 25 % advantage and 75% disadvantage b/c previously we have multiple opportunities to work but now we have only this so this is not a promotion.

Interviewer: How did you see the effect of COVID in working condition of your society?

Respondent: Now I see a lot of peoples staying at their home b/c of COVID so over all it has a lot of impact.

Interviewer: Can you give us the impact on your home?

Respondent: Previously I used to work and support our monthly expense like purchasing Taff and the like so now we have decreased it by half. For example, we used to eat one bread now we are eating half. As I told you before I was learning my accounting at collage so I do not have any savings so it has a lot of effects.

Interviewer: I would like to say thank you for your explanation so from our discussion we have if you need anything to add on let me give you a chance.

Respondent: I want to put stress on the home issue I am living for the past 11 yrs on this house and I was asking for multiple times to give me the kebele home but no one lessens me they told me as a reason that old peoples are living on the street and they are the priority if this problem fixed for me, I will help myself in getting work on what I have trained on and help my family. My wife has also completed 10th grade and she will continue to. Living in Addis having a home is very necessary.

Interviewer: I think you have mentioned it earlier on the changes in working condition b/c of COVID so what other changes did you noticed?

Respondent: Previously peoples will shake their hand, give kiss to say hi. So, what we did is that we have put a water by using a plastic container like ''Rotto'' on the street and peoples will wash their hands when they come to our site or ''Ketena'' and peoples was not doing that so we were fighting for them to wash their hands and plus to that we are using face mask.

Interviewer: I think we did jump one point on fatherhood and parenting from whom you get information's on this area? Or is there anyone who fives you consult?

Respondent: The health professional at the facility will not give you much information as the level of your need b/c they have a work load so I give information from the society or from my Nabors I will give

much more attentions on how they are parenting their children's on how did they support their children's in school related works and follow his day so his children will be effective and reach on better place so I will take a lesson. I am spending time with my older son like his friend he will tell me his day and what happens in his school so we have an open relationship. Parenting is not just giving or supporting my money they need open discussion and spent much more time. I have saved some money each day and I have punched a used bicycle and he is so happy.

Interviewer: You have Sayed that discussion on the family so with whom they need to discuss.

Respondent: My son needs to tell me everything if he tells to someone else it is not good. So, he will ask me what he needs and If I can I will buy it immediately if not I will tell him to give me some time for that so we have a very good interaction even with my wife. For example, we will discuss on the holiday expenses as I told you we are living in a planned way we already distributed our money for all expenses so we will have to discussion on how to pass this holiday too.

Interviewer: Who will give you consult outside of your home?

Respondent: My boss his name is _____ and he is very humble and thoughtful and currently I am working with him. If I have anything to discussion, I will tell him even if I need money, he will give me like 400-500 birr. So, he is very good person and I want to thank him.

Interviewer: So, you are saying you have a friend you can discuss on if you face this kind of challenges.

Respondent: Yes, he is more than that he is like a family. But plus, to that my relatives are living In Addis too and in addition I am Orthodox so we have a gather once a month at the day of 27 so we will discuss and share plus we will visit a member who is sick and the like this gathering ''Mehaber'' is now 17 yrs my brother my Anti and uncle is also a member so I have joined them we are around 25. Usually if day 27 did not on Sunday we will post pond it to the next week Sunday and we have even a very good discussion yesterday.

Interviewer: This is all what we have for discussion but if you have anything to add we will give you a chance.

Respondent: What I want to add is my youngest brother is living with me now so I wasn't to send him to school and finish his learnings. I will support him I do not want him to work anything also my uncle doughty will start night school my child will be turning to grade 5 this is all I want to add.

Interviewer: I would like to thank you for your time on behalf of the study groups.

Respondent: Ok thank you.

REMARK:

Upon completing the interview, we asked him what he thought about the interview. And he mentioned that raising his second child was not difficult. He told us that when he had his first child… it was unplanned and it was immediately after moving to addis and he had no idea what to do. He said they

tried living together with his partner then (the mother of the child) but it was difficult. So he told us they split up. The mother took the child and left him with her parent in the rural areas.

After a while, once he got accustomed to the city life and got a small house to live in. He said he went and brought his child… since then he had been a single dad until he got re-married to his current wife. He mentioned that being a single dad was difficult, but now with a partner he said things are smoother.
